# Isometric Scaling in Developing Long Bones Is Achieved by an Optimal Epiphyseal Growth Balance

**DOI:** 10.1371/journal.pbio.1002212

**Published:** 2015-08-04

**Authors:** Tomer Stern, Rona Aviram, Chagai Rot, Tal Galili, Amnon Sharir, Noga Kalish Achrai, Yosi Keller, Ron Shahar, Elazar Zelzer

**Affiliations:** 1 Department of Molecular Genetics, Weizmann Institute of Science, Rehovot, Israel; 2 Department of Statistics and Operations Research, Tel-Aviv University, Tel-Aviv, Israel; 3 Laboratory of Bone Biomechanics, Koret School of Veterinary Medicine, The Robert H. Smith Faculty of Agriculture, Food & Environment, The Hebrew University of Jerusalem, Rehovot, Israel; 4 Faculty of Engineering, Bar Ilan University, Ramat Gan, Israel; University of California Irvine, UNITED STATES

## Abstract

One of the major challenges that developing organs face is scaling, that is, the adjustment of physical proportions during the massive increase in size. Although organ scaling is fundamental for development and function, little is known about the mechanisms that regulate it. Bone superstructures are projections that typically serve for tendon and ligament insertion or articulation and, therefore, their position along the bone is crucial for musculoskeletal functionality. As bones are rigid structures that elongate only from their ends, it is unclear how superstructure positions are regulated during growth to end up in the right locations. Here, we document the process of longitudinal scaling in developing mouse long bones and uncover the mechanism that regulates it. To that end, we performed a computational analysis of hundreds of three-dimensional micro-CT images, using a newly developed method for recovering the morphogenetic sequence of developing bones. Strikingly, analysis revealed that the relative position of all superstructures along the bone is highly preserved during more than a 5-fold increase in length, indicating isometric scaling. It has been suggested that during development, bone superstructures are continuously reconstructed and relocated along the shaft, a process known as drift. Surprisingly, our results showed that most superstructures did not drift at all. Instead, we identified a novel mechanism for bone scaling, whereby each bone exhibits a specific and unique balance between proximal and distal growth rates, which accurately maintains the relative position of its superstructures. Moreover, we show mathematically that this mechanism minimizes the cumulative drift of all superstructures, thereby optimizing the scaling process. Our study reveals a general mechanism for the scaling of developing bones. More broadly, these findings suggest an evolutionary mechanism that facilitates variability in bone morphology by controlling the activity of individual epiphyseal plates.

## Abstract 2

A novel computational approach for studying bone morphogenesis reveals that the longitudinal proportions of developing long bones are accurately maintained throughout elongation by the balance between proximal and distal growth rates.

## Author Summary

One of the major challenges that developing organs face is scaling, that is, the adjustment of physical proportions during the massive increase in size. Bone superstructures are projections that typically serve for tendon and ligament insertion or articulation. Therefore, superstructure position along the bone is crucial for musculoskeletal functionality. As bones are rigid structures that elongate only from their ends, it is unclear how superstructure positions are regulated during growth to end up in the right locations.

Here, by analyzing a massive database of micro-CT images of developing mouse long bones, we show that all superstructures maintain their relative positions throughout development. It has been suggested that during development, superstructures are continuously reconstructed and relocated along the shaft, a process known as drift. However, our analysis reveals that most superstructures did not drift at all, implying the involvement of another mechanism. Indeed, we identify a novel mechanism for bone scaling, whereby each bone exhibits a specific and unique balance between the growth rates from its two ends, which accurately maintains the relative position of its superstructures. Moreover, we show mathematically that this mechanism minimizes the cumulative drift of all superstructures, thereby optimizing the scaling process.

## Introduction

The three-dimensional (3D) morphology of bones is fundamental to the ability of an organism to move, feed, and protect itself. Yet, we know little about the mechanisms that regulate bone shaping during development. Most bones develop by endochondral ossification, a process whereby an anlage of cartilage roughly in the shape of the future bone is formed and then gradually replaced by mineralized tissue [1–5]. The replacement of cartilage by ossified tissue is regulated by the growth plates, which are composed of chondrocytes that maintain a tightly controlled dynamic balance between proliferation and differentiation. The process begins by the formation of a primary ossification center approximately in the middle of the cartilaginous anlagen, which is composed of two growth plates facing in opposite directions. During development, the two growth plates continuously replace cartilage with ossified tissue while moving away from each other. This causes a gradual increase in the length of the bone [6] until maturity is reached and elongation ceases.

In recent years, a vast amount of effort has been invested in deciphering the molecular mechanisms that control the growth plate. These efforts have resulted in the identification of molecular pathways that regulate chondrocyte proliferation and differentiation [2,5,7]. Interestingly, although these molecular mechanisms seem to be generic for all growth plates, variations in elongation rates between growth plates have been reported [8–14]. To date, the mechanism that controls the specific growth rate of each growth plate is still missing.

Although bidirectional elongation is a universal mechanism for bone growth, it nevertheless introduces a major challenge to bone morphogenesis. A fundamental characteristic of the unique morphology of each long bone is a set of protrusions of varying shapes and sizes, which are scattered along the exterior of the bone and thus break its morphological symmetry. These superstructures, known as bone ridges, tuberosities, condyles, etc., are necessary for the attachment of tendons and ligament as well as for articulation. To perform these functions they are located at specific positions along the bone [15–17]. Bone superstructures emerge during early skeletogenesis [18, 19]. During growth, bones elongate extensively by advancement of the two growth plates away from the superstructures. It is therefore expected that during elongation, superstructures would remain at their original position near the center of the bone. Nevertheless, the end result is proper spreading of superstructures along the mature bone, which clearly implies the existence of a morphogenetic mechanism that corrects their locations.

There are two possible morphogenetic routes that can lead to the proper positioning of superstructures. The first is that superstructures emerge at their final relative position along the bone and then maintain this position throughout the entire growth period. This strategy is known as isometric scaling. Another possibility, known as allometric scaling, is that superstructures gradually converge from their initial to their final relative positions during growth. The question of isometric versus allometric scaling is particularly interesting in bones. An ossified bone is a rigid object and so are the superstructures protruding from it, implying that they cannot be relocated by means of cell migration or proliferation. Therefore, any scaling mechanism must be adapted to overcome these physical restrictions.

Although this aspect of bone morphogenesis has been largely neglected, Bateman [20] has suggested a mechanism for modifying the position of superstructures along the bone. This mechanism is based on gradual drift along the shaft achieved either by mineral deposition at either the proximal or the distal surface of the superstructure, or by synchronous deposition and absorption of mineral at opposite surfaces, collectively known as bone modeling. This results in drifting of the superstructure towards the deposition side or away from the absorption side. Although this hypothesis offers a reasonable strategy for the scaling of developing bones, its contribution has not been examined yet. In this study, we show that long bones are scaled isometrically from early embryonic stages to adulthood. Moreover, we identify a new morphogenetic mechanism that, combined with bone modeling, maintains the relative position of superstructures by controlling the balance between epiphyseal growth rates. Finally, we show that the growth balance of each bone is optimized for minimizing the drifting activity of its elements, thus facilitating isometric scaling in long bones.

## Results

### Longitudinal Proportions in Long Bones Are Maintained throughout Development

During skeletogenesis, bone superstructures emerge at an early stage [18,19], after which the long bones increase considerably in length. To gain understanding on how the relative position of superstructures is regulated during elongation, it is first necessary to document the distribution of superstructures along the bone throughout the growth period. For that, we established a database of 3D micro-CT images of the six long bones of the limbs, namely humerus, radius, ulna, femur, tibia and fibula, between embryonic day (E) 16.5 and postnatal day (P) 40.

**Fig 1:**
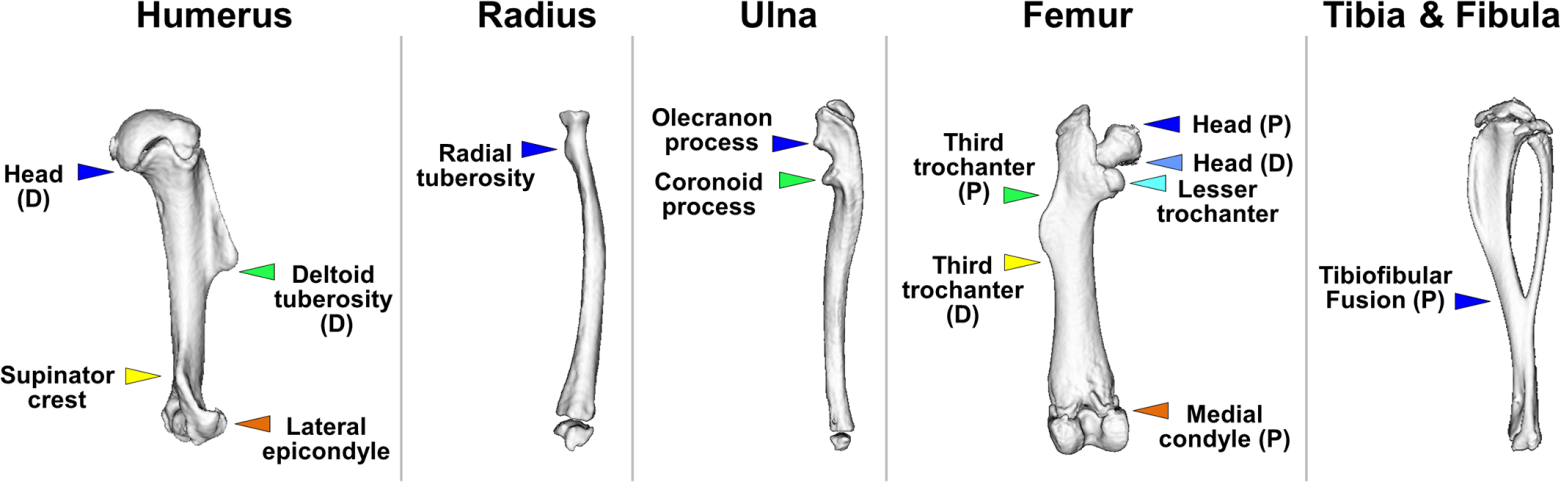
Symmetry-breaking elements define longitudinal proportions in long bones. Three-dimensional reconstruction from micro-CT scans of the six limb long bones at P40. The longitudinal position of each element is indicated either by a single colored mark on the tip of the superstructure or by two colored marks at its proximal (P) and distal (D) margins, depending on the size and the morphology of the superstructure. Margins that overlap with the end of the bone or with a growth plate were not marked.

**Fig 2:**
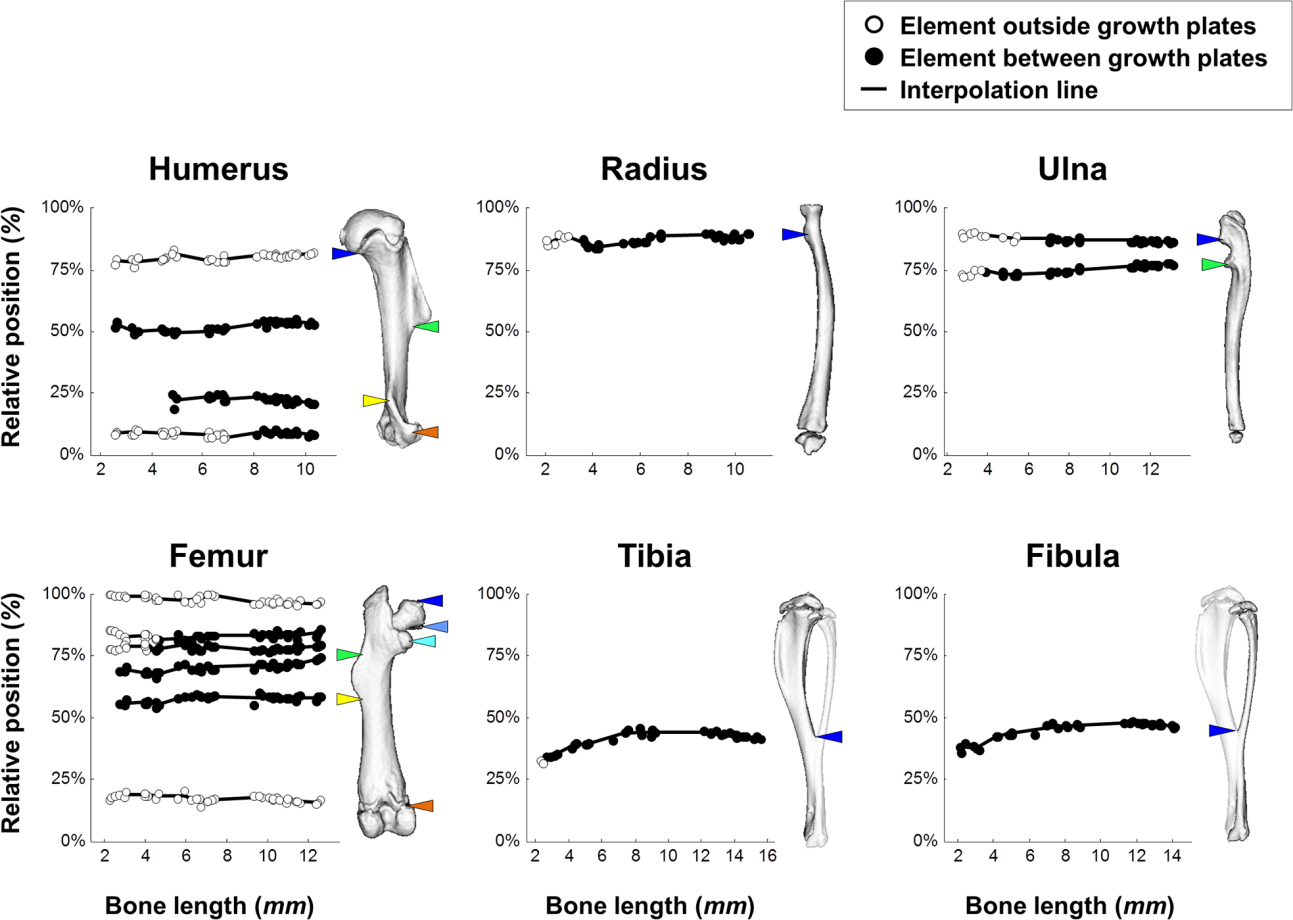
Longitudinal proportions in long bones are maintained throughout development. Graphs showing the relative position of each element throughout development. On the right of each graph is a 3D representation of an adult bone with colored marks of element locations. The horizontal curve lines indicate that the relative position of all elements is highly preserved throughout the entire growth period (average range of deviation, 4.4%). Data for this figure are provided in S1 Data.

In each bone, anatomical points that mark the longitudinal position of superstructures, collectively referred to in the following as symmetry-breaking elements, were identified. These included the tip; the proximal and the distal margins of tuberosities, trochanters, crests, condyles, and processes; and the fusion zone of the tibiofibular complex (Fig 1). Then, the relative position of each element between the proximal and the distal ends of the bone was measured (see Materials and Methods and S1 Fig). Because the focus of this work was on longitudinal scaling, in all the analyses described in the following element positions were calculated as a function of the total bone length. As evident in Fig 2, the relative position of all elements in all bones was preserved throughout the entire growth, as the average range of deviation was 4.4%, with the exception of the tibiofibular fusion point (14.3%). These results show that longitudinal scaling in growing long bones is highly isometric.

### The Cortical Core Is a Reliable Salient Feature for Anatomical Matching between Developing Long Bones

Drift by bone modeling was previously suggested to preserve the relative position of superstructures in mineralized bones [20]. As, to our knowledge, this hypothesis has not been tested thoroughly during development, we proceeded to analyze the drifting patterns of different bone elements. To extract drifting patterns from a temporal sequence of images, it is first necessary to standardize the spatial positions of all imaged bones of the same type by applying an image registration algorithm [21–23]. This would allow defining a standard longitudinal axis along which drifting can be quantified. Since bones are rigid structures that cannot undergo deformation and change only by means of mineral deposition and absorption, an anatomically correct matching between two bone images should be estimated by rigid registration, namely by pure translation and rotation of the bone. Registration based on matching salient and anatomically similar geometrical features such as points, surfaces, lines, or volume segments is a common approach for the registration of biological images [22]. As the bone images depicting the morphogenetic sequence are acquired at different times during development, a valid salient feature must be temporally preserved. Moreover, for population-based analysis, it is also required that the morphology of the salient feature be similar in different animals.

**Fig 3:**
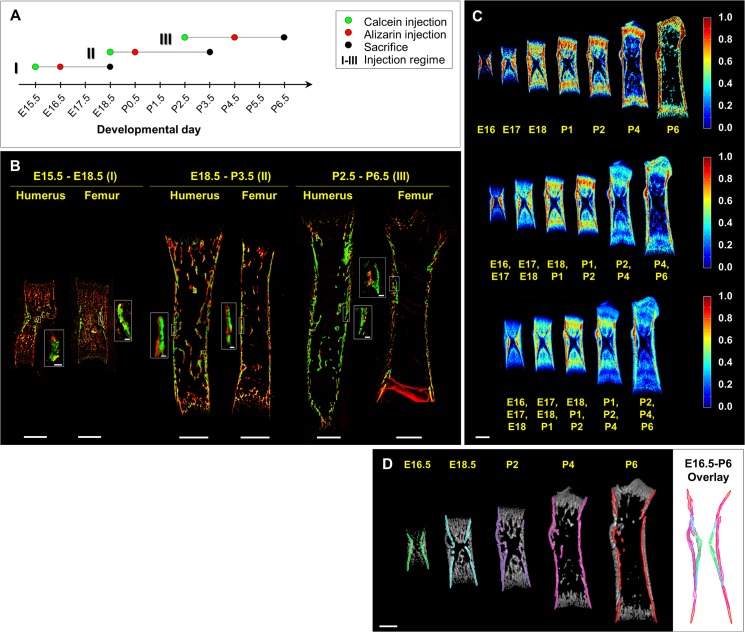
Accurate registration of bones from different developmental stages is achieved by matching the cortical cores. (A) A time chart of calcein and alizarin injection regimes. To cover the entire period effectively, three marginally overlapping regimes were applied. (B) Histological sections from each of the three injection regimes. At E18.5, green-labeled regions are seen around the middle part of the cortical core in both humerus and femur sections, indicating temporally preserved mineral structures. Similar temporal preservation of the cortical core is exhibited during perinatal and early postnatal development. (C) Color-coded statistical maps depicting the fraction of bones that are mineralized at each point in the bone, based on frontal slices of the femur. Clusters are composed of all bones from a single developmental day (top row), pairs (middle row), and triplets (bottom row) of consecutive developmental days. Regions that are highly preserved in population over several days (color-coded red; middle and bottom rows) are located mostly around the middle part of the cortical core. (D) A time series of frontal slices of the femur with the cortical core highlighted in each image with a different color. On the right is an overlay of all cortical core highlights following anatomical matching. The high overlap between segments demonstrates the potential of the cortical core to serve as a reliable salient structure for accurate anatomical matching of embryonic and early postnatal bones. Scale bars: 500 μm in B–D, 25 μm in B insets I and II, and 50 μm in B inset III.

To identify such features, we first scrutinized fluorochrome-labeled developing bones for conspicuous mineralized structures that are temporally preserved in individual mice (Fig 3A and 3B). Mice were labeled twice during development, first with calcein (green), and then one or two days later with alizarin complexone (red). Hence, regions labeled only with calcein would indicate mineralized structures that had been deposited before the alizarin injection and maintained until the day of examination. To cover the entire period of embryonic and early postnatal development effectively, we applied three marginally overlapping injection regimes. Analysis showed that during the entire developmental period (E16.5-P6.5) in both humerus and femur, a thin and continuous layer of dense mineral exhibited high temporal preservation. This layer, referred herein as the cortical core, stretched along the inner side of the bone cortex around the mid-length of the shaft.

Next, we assessed the morphological preservation of the cortical core between different mice of the same age. For that, we manually registered all bone images to each other by matching the cortical cores and generated statistical maps that indicate the fraction of bones that are mineralized at each anatomical point (see Materials and Methods). High fraction values (≥0.7) would indicate that the morphology of the mineralized structure is preserved in bones of mice of that age group. As seen in Fig 3C (top row), the cortical core is highly preserved across the population in all age groups.

To further validate our results, we jointly and directly examined the temporal and the morphological preservation of the cortical core. To that end, we generated statistical maps that included all bones from either pairs or triplets of consecutive days (Fig 3C, middle and bottom rows, respectively). In all the maps, extensive regions belonging to the bone collar exhibited high levels of preservation.

Taken together, these results demonstrate the morphological preservation of the cortical core, both over time within individual bones and between bones of different mice of the same age group (Fig 3D). This preservation establishes the cortical core as a reliable salient structure for cross-stage registration of both prenatal and early postnatal bones of different mice that differ by up to 3–4 developmental days.

### An Algorithm for Rigid Registration of Multiple Bone Images

**Fig 4:**
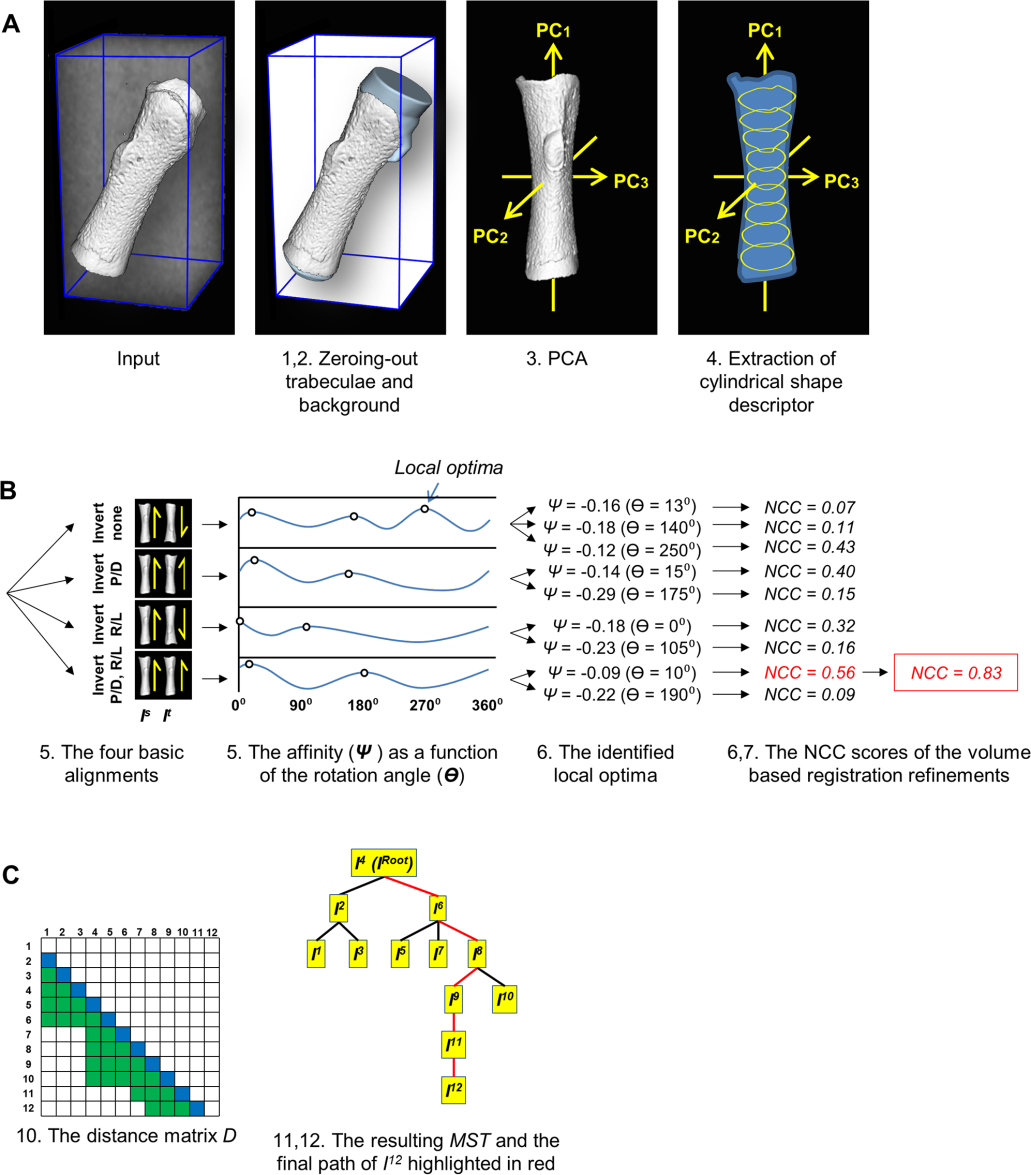
A flowchart of the algorithm for rigid registration of multiple bone images. The input of the algorithm is *N* 3D micro-CT images {*I* ^*N*^} and the index of the root image (*I* ^*Root*^; 1 ≤ *Root* ≤ *N*) to which all other images will be registered. **(A)** Preprocessing. For each input image *I* ^*N*^: 1. Zero-out all trabecular regions. 2. Zero-out all background regions. 3. Align bone to axes by applying PCA. 4. Extract cylindrical shape descriptor. **(B)** Pairwise Registration. For a pair of a source (*I* ^*s*^) and a target (*I* ^*t*^) image: 5. For each of the four basic alignments between the bones (with or without proximal-distal inversion × with or without right-left side inversion), calculate the affinity (***Ψ***) between the extracted shape descriptors of *I* ^*s*^ and *I* ^*t*^ as a function of the rotation angle (***Θ***) of *I* ^*t*^ about *PC1*. 6. From each local optimum in the calculated affinity function, perform several volume-based registration steps using NCC as a similarity measure and downhill descent as the optimization method. 7. Identify the path that reached the highest NCC score and optimize it using additional volume-based registration steps until convergence is reached. 8. If NCC <0.7, perform manual validation of the registration. **(C)** Agglomeration of pairwise to multiple image registration and interpolation. 9. Sort and re-index all bones based on their lengths, from shortest (“1”) to longest (“*N*”). 10. Register all pairs of bones *I* ^*s*^, *I* ^*t*^ (*s* < *t*) adhering to either one of the following criteria: *I* ^*s*^ and *I* ^*t*^ are lengthwise consecutive: *t* − *s* = 1 (subdiagonal entries in *D*; in blue), or *t* – *s* > 1 ∧ *Length*(*I* ^*t*^)/*Length*(*I* ^*s*^) ≤ 1.2 (non-subdiagonal entries in *D*; in green). Assign the resulting NCC score in the corresponding cell in *D*. 11. Calculate the MST of the graph inspired by *D*, with *I* ^*Root*^ being the root of the tree. 12. Infer all final transformations (*A* ^*N*^) based on the MST. 13. Transform/interpolate each image *I* ^*N*^ according to its inferred final transformation. The output is *N* 4 × 4 homogenous transformation matrices $\left\{A_{final}^{N}\right\}$, each aligns the corresponding input image *I* ^*N*^ to the root image *I* ^*Root*^, and the *N* transformed images. For more details, see Materials and Methods.

To recover the morphogenetic sequence of developing long bones, we derived an automated algorithm for rigid registration of multiple bone images (Fig 4). To compute an accurate transformation between the extracted cortical cores of two bone images, we start by coarsely estimating the registration by extracting a cylindrical shape descriptor from each image and matching the descriptors by custom-designed affinity function, based on the *L* ^*2*^ *-norm*. The registration is refined by applying normalized cross correlation (NCC) as a similarity measure, and downhill descent as an optimization scheme [24–27].

In order to align multiple images from different developmental stages, we apply a graph theoretic approach, in which we first compute the pairwise transformation between pairs of bones that differ in length by 20% at most, and retain the resulting NCC score of each pair in a distance matrix *D*_*N*×*N*_. We then compute the maximum spanning tree (MST) of the graph encoded by *D* with a preselected image serving as the root, and infer the final transformation of each image based on the path from the corresponding vertex in the MST to the root.

**Fig 5:**
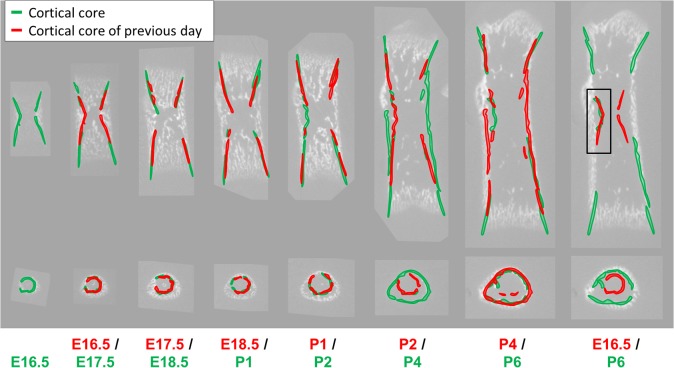
Visual evaluation of the algorithm. A time series of micro-CT images of the femur during development. To visualize the anatomical match, the cortical core is highlighted in green and the same region at the previous time point (in red) is overlaid on the image. The cortical cores are well matched in all pairs of ages, demonstrating the high fidelity of the algorithm to the true biological process. Top: frontal slices; bottom: transverse slices located about the longitudinal midpoint of the primary center of ossification.

To evaluate the results of the algorithm, we conducted both visual and quantitative analyses (Fig 5 and S1 File, respectively). The results demonstrate the high accuracy and reliability of the registration algorithm as a method for recovering the morphogenetic sequence of long bone development in general and, in particular, for the extraction of drifting patterns of bone superstructures during development. The application of the algorithm on our database of ex vivo images was complemented by manual registration of all in vivo postnatal bone images, performed by matching irregular and conspicuous mineralization patterns in the cortical and trabecular regions.

### Element Drift Has a Restricted Role in Isometric Scaling of Long Bones

**Fig 6:**
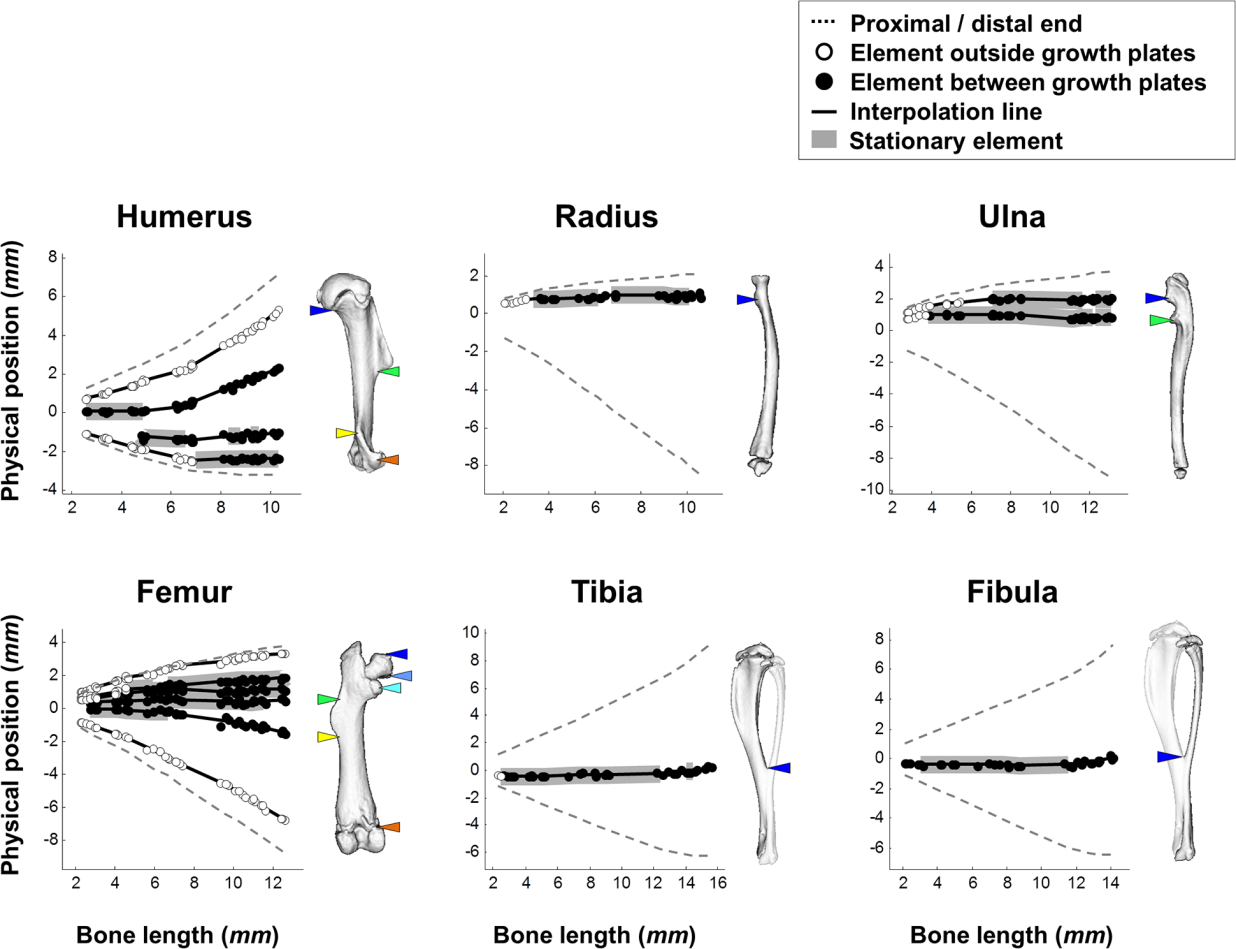
Element drift plays a restricted role in long bone isometric scaling. Graphs showing the physical position of each element throughout development. On the right of each graph is a 3D representation of an adult bone with colored marks of element locations. As indicated by thick gray background, elements remain stationary for periods ranging from several days to most of the developmental process. Data for this figure are provided in S2 Data.

To study the drifting patterns of various anatomical elements, we defined a variable termed “physical position” as the distance (in mm) of an element along the longitudinal axis of the bone from a predefined point of origin (S2 Fig). Changes in the physical position of an ossified element directly reflect its drift along the bone. As seen in Fig 6, the results showed both elements that drifted proximally, such as the distal margin of the humeral deltoid tuberosity (green marker), and distally, as the distal margin of the femoral third trochanter (yellow marker) during later developmental stages. In addition, variation in drifting rates between elements on the same bone were observed, such as the tip of the supinator crest in the humerus (yellow marker) compared with the distal margin of the deltoid tuberosity during late developmental stages.

Interestingly, our analysis also revealed that other elements remained stationary for periods ranging from several days to most of the developmental process (for definition of stationary elements, see Materials and Methods). These stationary elements were observed in different relative positions along the bone. For example, in the radius and ulna they were found near the proximal end, in the tibia and fibula at the center of the bone and, during late developmental stages of the humerus, such an element was found near the distal end. Moreover, during development some elements shifted modes between drifting and stationary.

The observed drifting of elements provides strong support for the contribution of this mechanism to long bone scaling. However, the identification of stationary elements in all bones suggests the existence of an additional mechanism that preserves the relative position of elements along the shaft, which is active throughout the development of long bones.

### The Fixed Plane Model for Longitudinal Isometric Scaling of Long Bones

**Fig 7:**
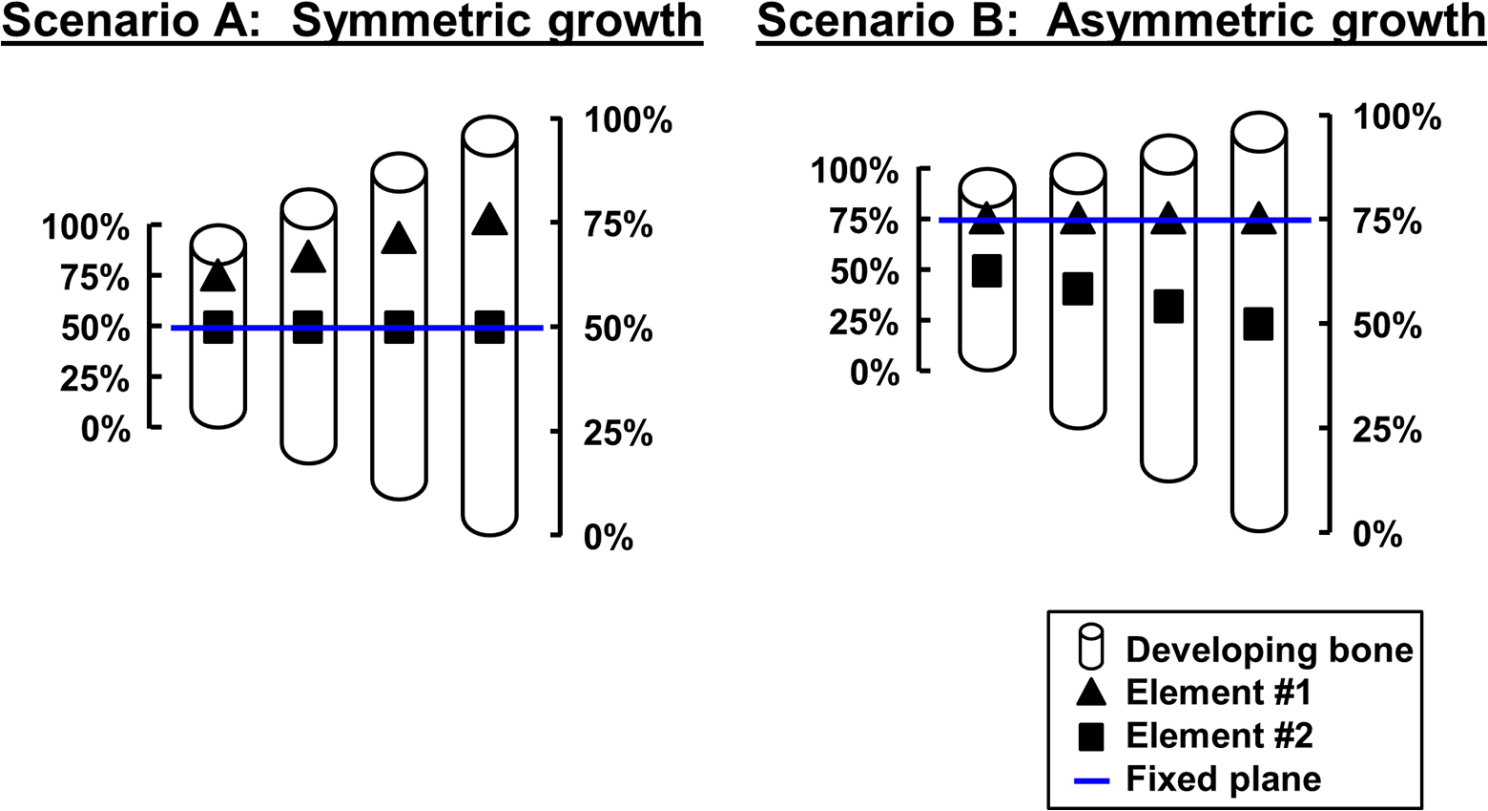
The fixed plane model for isometric scaling of long bones. Illustration of fixed planes formed at the transverse plane where the ratio between the distances from the distal and the proximal ends of the bone is equal to the ratio between the distal and the proximal growth rates. **(A)** When growth is symmetric, the relative position of an element located at 50% length (rectangle) is maintained, whereas an element located at 75% length (triangle) drifts proximally to maintain its relative position. **(B)** When distal growth rate is three times higher than proximal growth rate, the location of the FP is at the 75% length. As a result, the relative position of the triangle element is maintained, whereas the rectangle element drifts distally to maintain its relative position.

During longitudinal growth, the distance of elements from both ends of the bone increases, which is likely to change their relative positions. However, at any given time during the process, there is a single transverse plane along the shaft, referred to in the following as the fixed plane (FP), whose relative position remains unchanged. This plane is found where the ratio between its distances to the proximal and distal ends of the bone equals the ratio of growth rates at the two ends (Fig 7), as in the equation:

$$\frac{Distance\left(FP,ProximalEnd\right)}{Distance\left(FP,DistalEnd\right)}=\frac{ProximalGrowthRate}{DistalGrowthRate}$$

(see Materials and Methods for the mathematical formulation of the FP model).

Interestingly, according to this model, the relative position of elements located in close proximity to the FP will be preserved as well, thereby rendering any drifting activity unnecessary. Conversely, elements located far from the FP would have to drift away from the FP in order to compensate for the change in relative position. Moreover, the farther from the FP an element is located, the faster it will have to drift. Based on this notion, we hypothesized that preservation of the relative position of stationary elements is achieved by regulation of the balance between proximal and distal growth rates.

**Fig 8:**
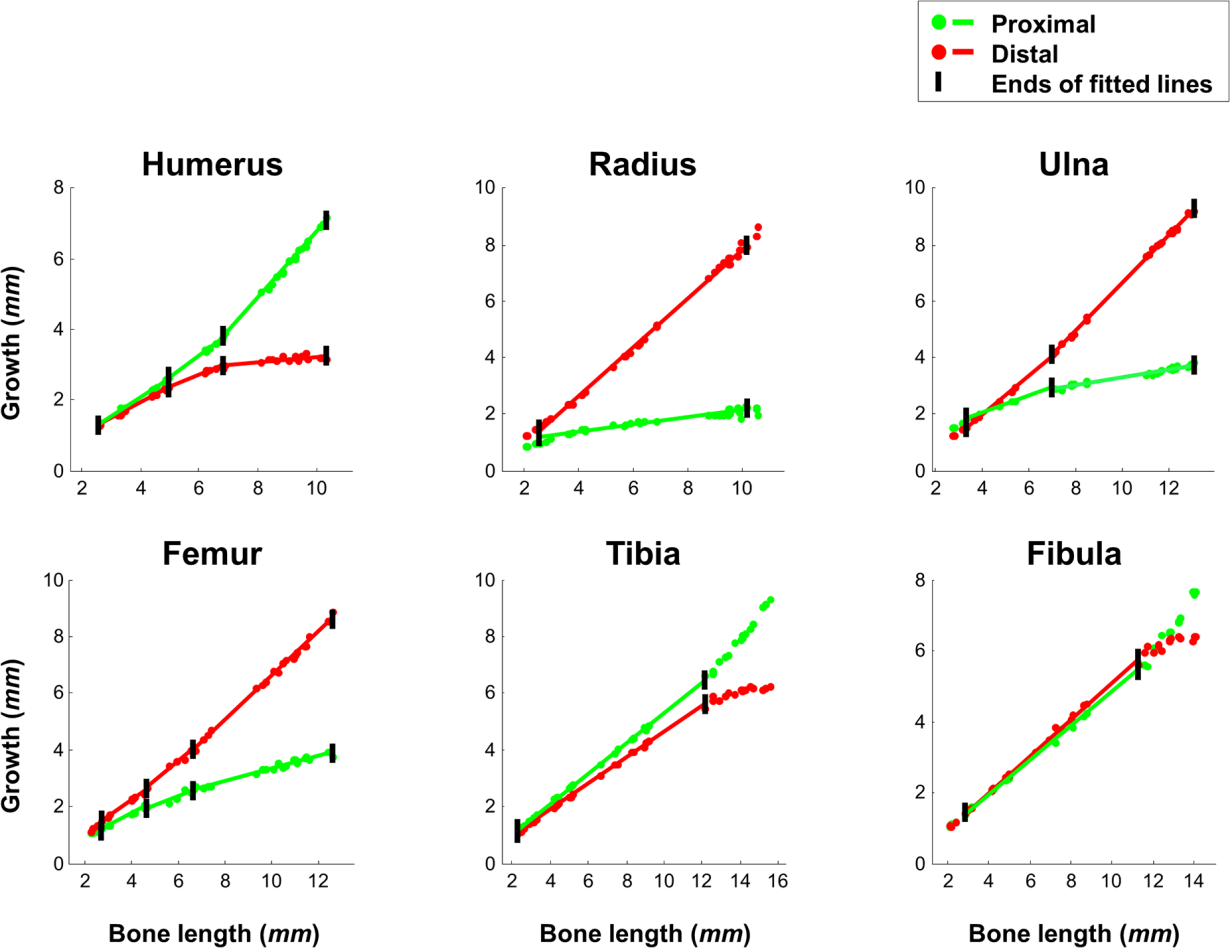
Growth balance remains invariant when stationary elements are present. Graphs showing growth at the proximal and distal ends of each bone as a function of the total length of the bone. Black vertical tags mark the ends of fitted lines, indicating time points at which an element either becomes stationary or begins to drift (the stationary elements are indicated by gray thick background in Fig 6). Regression analysis shows that at all intervals during which a stationary element is detected, the proximal/distal growth rate balance remains invariable (0.87 ≤ *R* ^*2*^ ≤ 0.99, *p*-value < 10e-05). Data for this figure are provided in S3 Data.

An obvious implication of this hypothesis is that passive preservation of the relative position of a stationary element over time necessitates that the specific balance between proximal and distal growth rates remains constant during that period. To date, a comprehensive analysis of proximal and distal growth patterns in each bone throughout the elongation process has not been performed. Therefore, to validate this implication, we extracted this information from the registered images of all long bones. When growth data is represented as a function of the total length of the bone, a constant balance between the growth rates is indicated by alignment of all data points along a straight line (Fig 8). Strikingly, statistical analysis of each bone showed that during all periods in which a stationary element was identified, a straight line provided a good fit to the data points (*R* ^*2*^ ranging between 0.87 and 0.99 for different bones and periods, *p*-value < 10e-05).

Another implication of this hypothesis is that for two different bone types, the closer the relative positions of their stationary elements are, the more similar their growth balance will be. Indeed, in both radius and ulna, where stationary elements are located adjacently and close to the proximal end of the bone, the relative contributions of distal growth were similar and significantly higher than those of proximal growth. In contrast, during late development of the humerus, where the stationary element is located near the distal end of the bone, the relative contribution of distal growth is significantly lower than that of proximal growth. Taken together, these results demonstrate the high agreement of the specific growth patterns of each bone with our model and, thus, support a potential role for differential longitudinal growth in regulating long bone isometric scaling.

### Growth Balance Maintains the Relative Position of Stationary Elements

In order to provide direct evidence for the role of the mechanism that regulates growth balance in preservation of the relative position of stationary elements, it is necessary to demonstrate high spatial proximity between the FP and stationary elements. We therefore proceeded to determine the physical position of the FP in each bone during elongation based on the balance between proximal and distal growth rates (for the calculations, see Materials and Methods).

**Fig 9:**
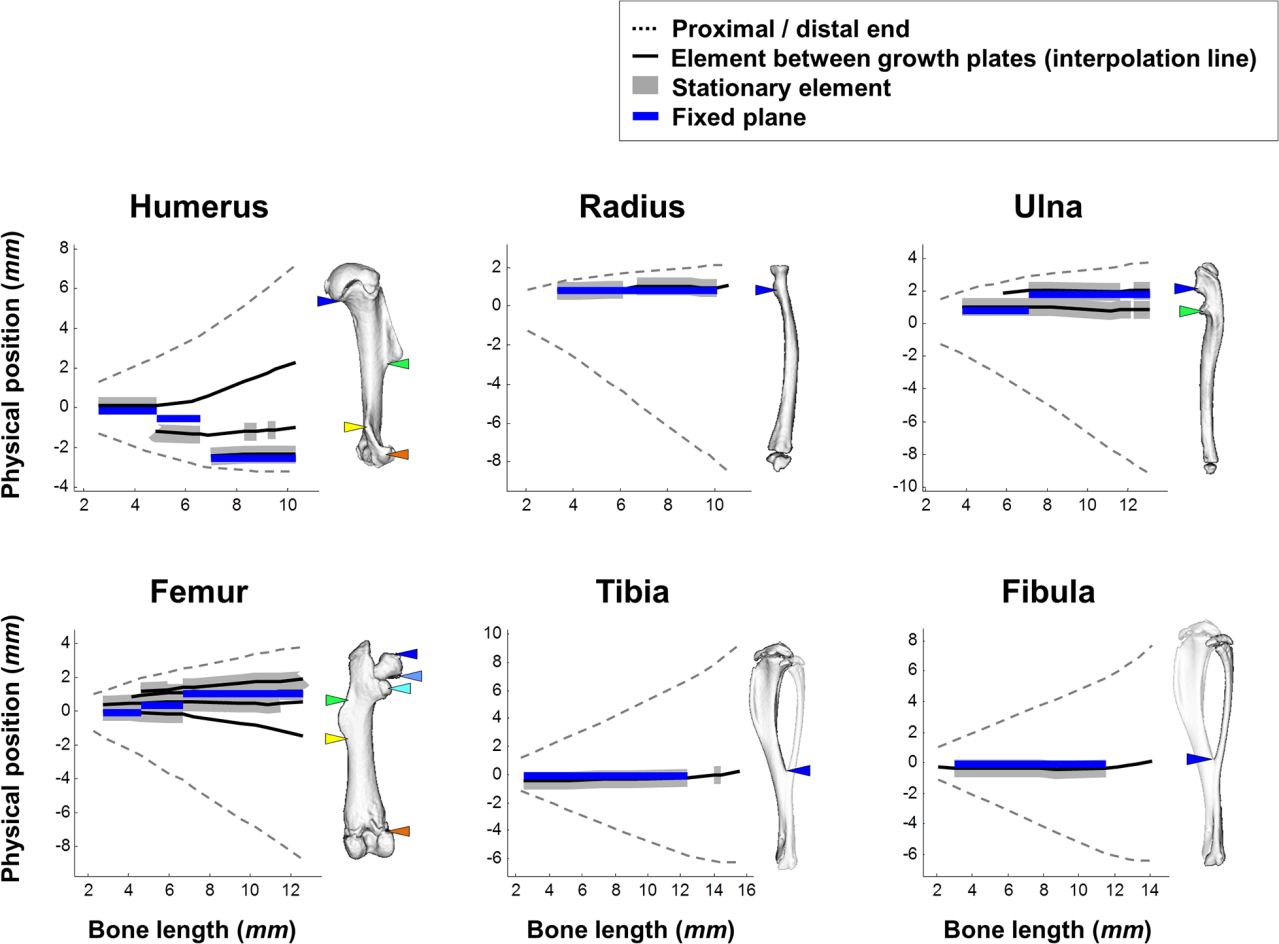
Isometric scaling is achieved by synchronization between element drifting patterns and growth balance. Graphs showing the physical positions of all elements residing between the growth plates and of the fixed plane in each bone as a function of total bone length. Stationary elements are highlighted with thick gray background and the physical position of the FP is marked by a blue line. On the right of each graph is a 3D representation of an adult bone with colored marks of element locations. As predicted by our model, elements located in high proximity to the FP remain stationary. Conversely, elements located distantly from the FP drift away from the FP, as the longer the distance, the farther the drift will be. Data for this figure are provided in S4 Data.

As seen in Fig 9, during most stages at which a stationary element has been identified, the FP was found in extremely high proximity to it. For example, throughout the period of E18.5–P32, the radial tuberosity (blue marker) is highly stationary (range of relative position, 84%–89%) and is also in close proximity to the FP (relative position, 87%). Conversely, all elements located at a distance from the FP drifted in a rate and direction that corresponded with their position relative to the FP, as predicted by the FP model. For example, during the last stages of humerus development, the distal margin of the deltoid tuberosity (green marker) drifted proximally at a higher average rate (48.3 μm/day) than the drift exhibited by the tip of the supinator crest (8.2 μm/day; yellow marker), which was closer to the FP.

Taken together, these results provide direct evidence that the relative position of stationary elements in developing long bones is protected by a mechanism that maintains a unique and dynamic balance between proximal and distal growth rates of each bone. Moreover, they show that element drift serves as a complementary mechanism for preservation of the position of elements residing away from the FP, as both direction and rate of the drift correspond closely to the position of the element relative to the FP.

### Growth Balance Is Optimized for Minimum Element Drifting

Our results show that isometric scaling in long bones is achieved by coordination between the growth balance mechanism and element drift. However, the ability of the drifting mechanism to compensate for changes in the relative positions of elements raises the question of the necessity of a growth balance mechanism for the scaling process, as an alternative to symmetric growth in all bones. The process of element drift involves deposition and absorption of mineral by osteoblasts and osteoclasts, respectively [20]. Therefore, setting the growth balance to form a FP in high proximity to elements would reduce the required level of drifting and is thus likely to facilitate bone scaling. In light of this notion, we hypothesized that the growth balance is optimized for minimizing the drifting activity of symmetry-breaking elements in each bone.

To examine this hypothesis, we first inspected the tendency of the FP to be located in high proximity to symmetry-breaking elements during growth in different bones. As can be seen in Fig 9, in each bone and at almost any time point during development, there is at least one element whose relative position is protected by the FP. Moreover, in bones where the FP is relocated, it shifts from one element to another. These results provide initial evidence for the bias of the growth balance mechanism towards minimizing drifting activity.

**Fig 10:**
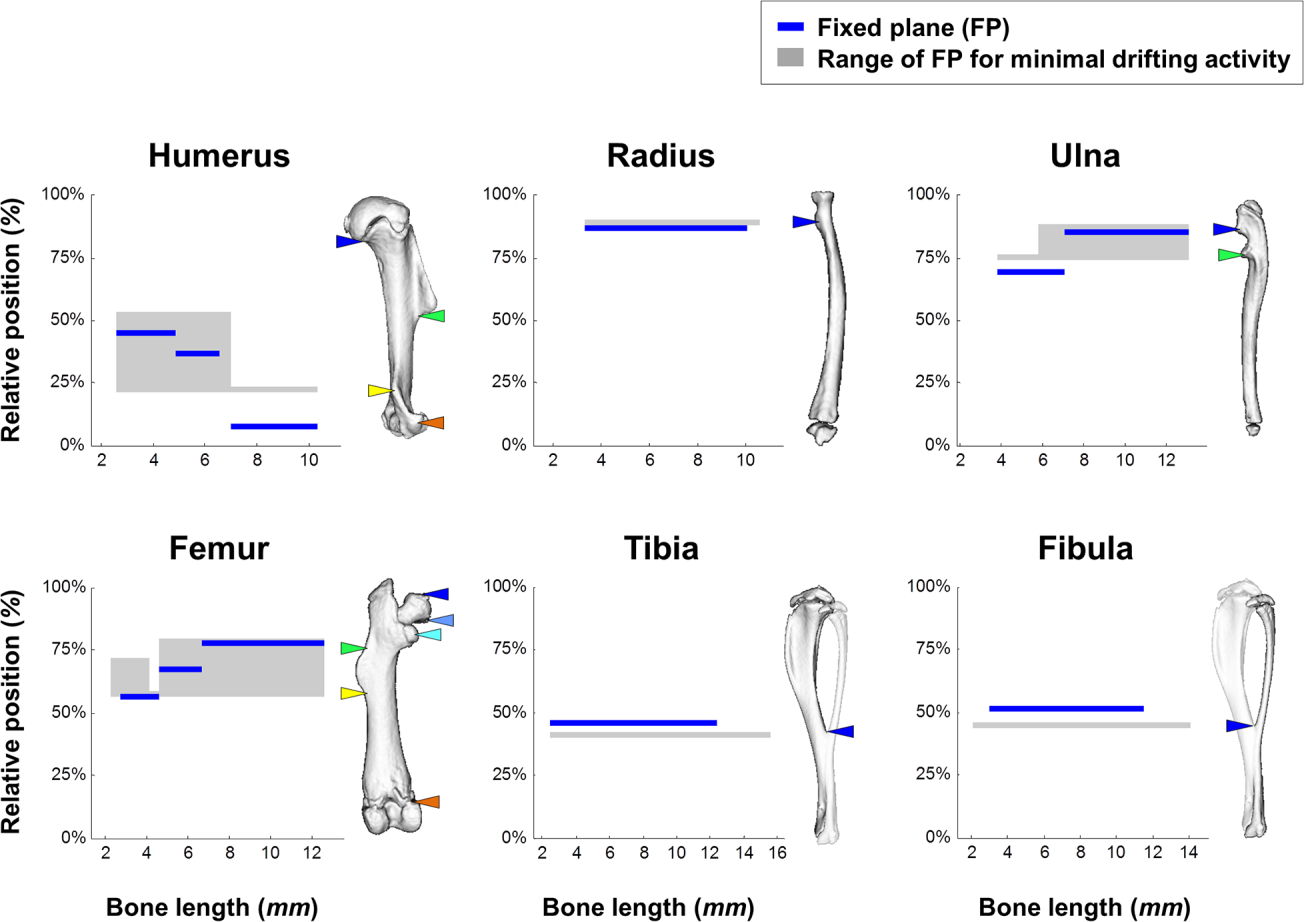
Growth balance is optimized for minimum element drifting activity. Graphs showing the range of relative positions of the FP in which the total drifting activity of symmetry-breaking elements is minimal and the actual relative position of the FP in each bone as a function of total bone length. On the right of each graph is a 3D representation of an adult bone with colored marks of element locations. Throughout the development of all bones, the FP either overlaps or is in high proximity to the range of values that leads to minimal element drifting activity. Data for this figure are provided in S4 Data.

In order to test our hypothesis quantitatively, we designed a “drifting cost” assay for calculating the growth balance that minimizes the total distance (in mm) drifted by all elements of a bone at each time interval during development (Fig 10; see Materials and Methods). Results showed that throughout the entire development of all long bones, FP position either overlaps or is in high proximity to the range that minimizes drifting activity. To evaluate statistically the likelihood of this proximity, we performed a permutation test under the null hypothesis that the position of the FP is not affected by the position of symmetry-breaking elements (see Materials and Methods). The results (*p*-value = 0.0013) clearly rejected the null hypothesis. Taken together, these results provide strong evidence that the growth balance mechanism is optimized for minimizing the drifting activity of symmetry-breaking elements.

## Discussion

In this work, we show that long bones are scaled isometrically throughout development. From the cellular level to the entire organism, growth in size typically necessitates changes in the physical proportions between the constituent parts in order for the system to remain functional [28,29]. Therefore, allometry is the prevailing mode of growth in both development and evolution [30–36]. During mouse development and growth, long bones elongate on average by more than five times, while the body mass increases by more than 20 times. Given the magnitude of these changes, one would expect that bones undergo allometric growth. However, our striking results show that apart from a minor deviation of the tibiofibular junction, longitudinal scaling of all long bones is clearly isometric throughout elongation. This persistent preservation implies that maintaining physical proportions of elongating bones has been highly significant in the evolutionary success of vertebrates.

To the best of our knowledge, no comprehensive investigation of the implications of isometry during skeletal growth has ever been published. Yet, it can be speculated that isometry has important biomechanical and/or locomotor advantages. One of the main challenges that developing bones face during growth is the necessity to withstand increasing mechanical forces applied both by the body mass of the organism and by muscle activity [37,38]. Recently published results of finite element analysis on bones of xenarthrans [16,31] suggest that the relative position of bony superstructures, such as the femoral third trochanter, is regulated in order to mitigate the biomechanical consequences of a 100-fold increase in body mass during phylogenesis. This suggests that maintenance of the relative position of bony superstructures may have been selected to optimize the ability of bones to withstand the increase in bending loads during development as well. In this aspect it is interesting to note that the shape and relative proportions of skeletal elements, which are key determinants of the mechanical integrity of bones, scale close to isometrically when considered over nearly the entire size range of mammalians [39].

Another hypothesis is that isometry allows the maintenance of complex muscle firing patterns involving many muscles by preserving the positions of muscle attachment sites during growth. This strategy was proposed for the swallowing action in humans [40], in which despite extraordinary growth, the relative positions of muscle insertions in the neck remains constant during ontogeny to prevent aspirating food and liquids. In the context of the axial skeleton, maintaining firing patterns directly relates to the evolutionary importance of highly coordinated motility activities such as running and climbing, which are essential for survival.

A third possibility relates to the connection between skeletal proportions and the unique gait of each animal [41–43]. Alteration in skeletal proportions has long been known as a major driving force in the generation of new and diverse phyla, each optimized to a different motoric repertoire [42,44]. In view of this, it would be reasonable to interpret the isometric nature of developing long bone scaling as a means to maintain certain locomotor properties throughout the lifetime of the organism. Alterations in the relative position of such elements would most likely result in different force/speed ratios, which would in turn modify motoric function and environmental fitness.

Thus far, the lack of reliable methods for quantitative recovery of the morphogenetic changes bones undergo during development has hampered the study of mechanisms that regulate long bones scaling. Our newly developed algorithm for rigid registration of multiple images enables accurate assessment of the morphogenetic sequence of each growing bone, allowing us to directly address this question. One mechanism that has been implicated in positional regulation of elements is drifting by mineral deposition and/or absorption at opposite surfaces of the element [20]. Utilizing our image registration algorithm, we provide here empirical evidence for the specific contribution of drifting to isometric scaling in each long bone.

Notwithstanding the advantages of this mechanism, drifting of an ossified element requires massive and continuous activity of osteoblasts and osteoclasts, taking a high energetic toll on the morphogenetic process. Moreover, when the element serves as an anchoring point for tendons and ligaments, these tissues must be repositioned too [45–47]. This toll can be reduced by maintaining relative position of elements during elongation, thereby reducing the need for drifting. For that to happen, the fixed plane in each bone needs to be in close proximity to symmetry-breaking elements. Indeed, our data clearly demonstrate that in all bones and throughout elongation, the location of the FP protects specific elements from the need to drift. We therefore conclude that the ratio between proximal and distal longitudinal growth rates, which is reflected by the location of the FP, is a key mechanism in bone scaling. Moreover, we show that the distance from the FP determines which elements are to remain stationary at each time interval, as well as the rate and direction of drift of non-stationary elements. Altogether, these findings establish growth balance between the plates as the mechanism that orchestrates long bone scaling by synchronizing between drifting and passive protection of stationary elements.

As mentioned, the rationale behind the existence of stationary elements is postulated to be energy saving during scaling. Indeed, our drifting cost assay shows that in all bones and throughout development, the location of the FP is either optimal or near-optimal for minimizing drifting activity. This implies that the optimization of the scaling process involves an accurate and dynamic balance between proximal and distal growth rates, which requires tight regulation of the activity of each growth plate throughout the elongation process.

Generic mechanisms that regulate growth plate activity by synchronizing chondrocyte proliferation and differentiation, such as the PTHrP–IHH (Gene ID: 19227, 16147, respectively) feedback loop, have been extensively studied [1,2,48–52]. However, our analysis demonstrates that each growth plate exhibits a distinctive activity pattern, resulting in a unique ratio between elongation rates at the two ends of each bone. Along the same line, previous reports have shown differential growth plate activity, which leads to asymmetric longitudinal growth [8,13,53]. Typically, forelimb bones tend to grow away from the elbow joint, whereas bones in hind limbs tend to grow toward the knee joint. These findings and ours clearly imply the existence of additional mechanisms that control the specific activity of each growth plate. Interestingly, some of these works were performed on other model animals such as rat [54], pig [11], rabbit [55–60], chick [61], and humans [10], suggesting that asymmetric growth of long bones is evolutionarily conserved across species.

To date, little is known about mechanisms underlying differential activity between individual growth plates [13,53,62,63]. Our results indicate that the balance between proximal and distal growth rates of each bone is constantly optimized to minimize bone modeling. This suggests the existence of a feedback mechanism that incorporates data relating to the relative positions of superstructures into the molecular and cellular mechanisms that control growth plate activity. The regulatory role of the perichondrium/periosteum in growth plate activity has long been recognized and has been the subject of considerable research [2,62–75]. Damage to the integrity of the periosteum, as a result of bone fracture [64] or circumferential division with or without stripping of the periosteum [65–67,74], was shown to lead to temporal acceleration of bone growth. This acceleration is typically accompanied by alteration in the balance between proximal and distal growth rates. Because the periosteal sheath is stretched over the entire external surface of the bone, including both the superstructures and the growth plates, it can pass to the growth plates signals concerning the relative position of superstructures.

Interestingly, Crilly [64] previously hypothesized that periosteal tension down-regulates growth plate activity, as the higher the tension level, the more inhibited growth plate activity is. Moreover, he postulated that the damaged periosteum forms a scar tissue at the site of destruction. This scar tissue, which anchors the periosteum into the bone, creates an independent tension level near each growth plate. As a result, a new growth balance is formed, which equals the ratio between the distances from the site of the scar to the two ends of the bone, therefore maintaining the relative position of the scar site. This hypothesis can be generalized to account for normal growth conditions as well. Superstructures can be considered as natural anchoring points for the periosteum into the ossified bone, either due to the insertion of tendons through them into the bone cortex, or by means of steric interference, such as in the tibiofibular junction. This results in a regulatory loop whereby the superstructures determine the tension levels of the two periosteal segments, which control the ratio of growth rates by inhibiting growth plate activity, which in turn maintains the relative position of the superstructure.

Since the perichondrium/periosteum is well known to express many signaling molecules that regulate growth plate activity [75–77], it is plausible to hypothesize that these molecules are involved in mediating the mechanical signals. For instance, mice homozygous for a targeted disruption of fibroblast growth factor 18 (FGF18; Gene ID: 14172), synthesized by perichondrial cells, exhibit increased chondrocyte proliferation, differentiation to hypertrophic chondrocytes and IHH signaling [76]. Other signaling molecules synthesized in the perichondrium, such as FGF9 (Gene ID: 14180) [76], multiple bone morphogenetic proteins (BMPs) and WNTs [77] have been shown to operate on receptors expressed on chondrocytes, further demonstrating the regulatory role of the perichondrium/periosteum on growth plate activity.

In this work, we uncover the isometric nature of longitudinal scaling of long bones during growth. Using a newly developed algorithm, we recover for the first time, to our knowledge, the morphogenetic sequence of developing long bones from early embryonic stages to maturity. These data enabled us to provide accurate assessments of both the specific activity of the different growth plates and the drifting patterns of symmetry-breaking elements along the bone shaft. Based on these analyses, we conclude that longitudinal growth patterns in each bone are adjusted to preserve isometry. The constant tendency of the growth balance to protect element positions strongly suggest that symmetry-breaking elements are involved in the mechanism that regulates the differential activity of growth plates.

## Materials and Methods

### Ethics Statement

For harvesting of embryos, timed-pregnant females were sacrificed by CO2 intoxication or cervical dislocation. Embryos and postnatal mice were sacrificed by decapitation with surgical scissors. All experiments were approved by the Institutional Animal Care and Use Committee (IACUC) at the Weizmann Institute of Science. For in vivo micro-CT imaging, mice were anesthetized with isoflurane (2-chloro-2-[difluoromethoxy]-1,1,1-trifluoro-ethane).

### Animals

C57/Bl6 mice (Harlan Laboratories, Jerusalem, Israel) were used for all analyses. For the generation of statistical maps and for validation of the automated registration algorithm only males were analyzed. Sex was determined in utero by PCR [78] and by external examination postnatally. At every developmental stage until P4, six to eight mice from at least three different litters were evaluated. At P6, two to four mice from two different litters were examined. Plug date was defined as E0.5. The gravid uterus was dissected out and suspended in a bath of cold phosphate-buffered saline (PBS), and the embryos were harvested after amnionectomy and removal of the placenta.

### Preparation of Soft Tissue for Micro-CT Scanning

Before scanning, harvested limbs were fixated overnight in 4% PFA/PBS and gradually dehydrated to 100% ethanol (dehydration sequence: 25%, 50%, 70%, and twice in 100% for 30 min each). Then, according to [79] tissue samples were soaked in 2% iodine/ethanol solution (Sigma) for 48–72 h, depending on limb volume, at 4°C. Lastly, tissue samples were washed twice in 100% ethanol for 30 min and kept until scan at -20°C.

### Micro-CT Imaging

For assessment of the distribution of symmetry-breaking elements along the shaft, bones at stages between E16.5 and P10 were scanned ex vivo using iodine contrast agent to allow visualization of cartilaginous tissue (in each age group, *n* ≥ 3). The rest of the images were obtained by sequential in vivo scans of four mice. The tibia and fibula were measured separately although they fuse early during development because their growth plates remain separated.

For the generation of statistical maps and for validation of the registration algorithm, samples were scanned ex vivo in PBS solution by an eXplore Locus SP micro-CT scanner (GE Healthcare, London, Ontario, Canada) at 45kVp and 120μA. For all scans, 900 projections over 360 degrees, with 4 frames averaged for each projection at an exposure time of 2,850 ms per frame, resulted in an isotropic voxel size of 7.139 μm. Voxel intensity was represented by data type int16. Calibration hydroxyapatite phantoms (GE Medical) were used to facilitate conversion of the linear attenuation of a given voxel to mgHA/cm³.

For measurements of relative and physical positions of element (described in the following) and of epiphyseal growth rates at E16.5 to P10, bone samples from three to six mice were scanned ex vivo in 100% ethanol by a Zeiss Xradia Micro XCT-400 x-ray microscope (Pleasanton, CA, United States) at 40kV and 200μA with a linear magnification of x0.5 or x4. Between 350 and 1,650 projection images were taken over 180 degrees at an exposure time of 500–7,500 ms per projection, providing a final isotropic voxel size of 4–18 μm. Optimal beam hardening was used in every reconstruction. Voxel intensity was represented by data type uint8. Following reconstruction, voxel size of all images was standardized to 7.139 μm using trilinear image interpolation.

For the same measurements in P20–P40 bones, bones of four mice were scanned in vivo by TomoScope 30S Duo scanner (CT Imaging, Germany) equipped with two source-detector systems in air medium at 41kV and 1mA. For all scan, 720 projections over 360 degrees at an exposure time of 125 ms per projection resulted in an isotropic voxel size of 36 μm. Voxel intensity was represented by data type int16. Calibration to HU was performed using a factory default module.

### Calculation of Relative and Physical Position of Elements

To calculate element position relative to the bone ends, all bones were reoriented such that the longitudinal axis of the bone was parallel to the vertical axis (*z*) of the image grid. Then, the vertical coordinate of the documented element (*E*_*z*_) and of both the proximal (*P*_*z*_) and distal (*D*_*z*_) ends of the bone were measured in voxel units. Finally, the relative position of the element (*E*_*RelativePosition*_) was calculated as:

$$E_{RelativePosition}\;=\frac{E_{z}-D_{z}}{P_{z}-D_{z}}\times 100$$

Calculation of the physical position of elements was done after image registration and determination of the origin of the vertical axis (*O*_*z*_). The vertical coordinate of the documented element (*E*_*z*_) was measured in voxel units. Finally, the physical position of the element (*E*_*PhysicalPosition*_; in *mm*) was calculated as:

$$E_{PhysicalPosition}\;=\left(E_{z}-O_{z}\right)\times dV_{z}$$

where *dV*_*z*_ is the physical size (*mm*) of the voxel in the vertical dimension.

### Histology

Mineral deposition was evaluated by intraperitoneal injections of calcein (Sigma # C0875; 2.5 mg/kg body weight) and alizarin complexone (Sigma # a3882; 7.5 mg/kg) into pregnant females and, postnatally, into cubs. Two mice were used for each injection regime. Prenatally harvested limbs were fixated overnight in 4% PFA/PBS, dehydrated to 100% ethanol, embedded in paraffin and sectioned at a thickness of 7 μm. Postnatally harvested limbs were fixated 24 h in 4% PFA/PBS and gradually dehydrated from 70% ethanol to 100% ethanol twice for 48 h each time. Then, samples were infiltrated and embedded in JB-4 Embedding Kit (Electron Microscopy Science #14270–00) and sectioned longitudinally at a thickness of 7 μm. Fluorescence was visualized by confocal microscopy.

Confocal imaging was performed using a Zeiss LSM 510 upright confocal microscope (Carl Zeiss, Jena, Germany) with an EC Plan-Neofluar 10x/0.3 objective, NA 1.0. Calcein fluorochrome was excited with a 488 nm argon laser and alizarin with 561 nm argon laser. Following imaging, all images of the same section were stitched using Microsoft Image Composite Editor (version 1.4.4.0). Contrast was increased by using the “auto contrast” tool of Google Picasa (version 3.9.137) and Matlab’s “imadjust” function.

### Micro-CT Image Segmentation and Binarization

To segment bone from background voxels, we calculated for each image a private global threshold using Otsu’s method [80] and binarized it to bone (1) and background (0). Threshold values, measured in milligrams hydroxyapatite per cubic centimeter, ranged between 128.5 mg HA/cm^3^ in early developing bones and 317.1 mg HA/cm^3^ in older bones. Then, to filter out background voxels that exceeded the threshold, all voxels that did not belong to the largest connected component (i.e. the bone) were zeroed out. Lastly, each image was manually inspected to assure the quality of the binarization.

### Generation of Statistical Maps for Morphological Preservation

First, micro-CT bone images were manually registered by matching between the cortical cores. Then, each image was segmented and binarized as described above. To allow application of arithmetic operations between binarized images, the data type of all images was casted from Boolean to floating point. To generate statistical maps of morphological preservation over all bones belonging to the same age group, all binarized images of the following age group were averaged: E16.5 (*n* = 7), E17.5 (*n* = 8), E18.5 (*n* = 8), P1.5 (*n* = 7), P2.5 (*n* = 7), P4.5 (*n* = 8) and P6.5 (*n* = 3). To generate statistical maps of morphological preservation over all bones belonging to pairs (e.g. E16.5, E17.5) or triples (e.g. E16.5, E17.5, E18.5) of consecutive days, all binarized images of each group were averaged. Lastly, statistical maps were color-coded such that highly preserved regions are shown red and little preserved regions in blue.

### The Registration of Multiple Images

We start by addressing the particular case of pairwise image registration. To avoid the potential interference of non-overlapping image segments while matching the cortical cores, we zero-out the trabecular bone segments (see S2 File) in both source (static) and target (transformed) images using an in-house software, as well as all background segments by applying Otsu’s global thresholding method [80]. Although the remaining segments include cortical regions other than the core, this approximation provides registration results of comparable quality to those estimated using the cortical core alone.

In order to estimate the spatial transformation that anatomically aligns the target and source images, we apply a volume based registration approach that utilizes normalized cross correlation (NCC) as a similarity measure and downhill descent as the optimization method [24–26]. Since the shape of both registered objects is cylinder-like, initiating the registration using an arbitrary transformation might result in local optima, such as matching the proximal end of one bone to the distal end of the other. Thus, in order to initiate the optimization in the vicinity of the global NCC optimum, we compute a coarse estimate of the registration using a cylindrical shape descriptor of the bone cortex. The descriptor, referred to as the *circumferential profile* (CP): *ψ* ∊ ℝ_+_^9,360^, is a polar representation of the external contour of the cortex at nine equidistant transverse slices along its length (i.e. at 10%, 20%, …, 90% length between the proximal and distal ends of the cortex), at 360 equi-angular sample points along the contour of each slice. The radii are measured from the geometric center of that section. We first apply principal component analysis (PCA) on the point cloud consisting of all nonzero voxels, to allow direct extraction of the descriptor from transverse slices. Thus, the longitudinal dimension of the bone (PC1) is aligned with the *x*-axis, and all transverse slices are aligned with YZ planes.

A descriptor of a target image (*ψ* ^*t*^) is matched to a descriptor of a source image (*ψ* ^*s*^) using an affinity function based on the *L* ^*2*^ *-norm*:

$$\Psi^{s,t}\left(\theta \right)=-\frac{1}{9}\sum_{l=1}^{9}{\left\|\psi^{t}\left[l,\left(1,\ldots ,360\right)+\theta \right]-\psi^{s}\left[l,\left(1,\ldots ,360\right)\right]\right\|}_{2}$$

where for a given angle *θ*, the affinity value is the average Euclidian distance between the circumferential profile of *I* ^*t*^ rotated by *θ* about PC1, and that of *I* ^*s*^. Therefore, the set of local optima values of Ψ^*s*,*t*^ is likely to include the vicinity of the global NCC optimum as well.

We also consider right/left inversions (mirroring), and proximal/distal inversions of *I* ^*t*^, to identify all local optima of Ψ^*s*,*t*^. In order to improve the localization of the vicinity of the global NCC optimum, we apply several volume-based registration steps, initialized at each identified point of local affinity optimum. The path resulting in the highest NCC score is further optimized by additional volume-based registration refinement steps, until convergence is reached. A pair of images that fails to achieve an NCC score of 0.7 or higher is marked as an erroneous match and undergoes manual validation and, possibly, correction by the operator.

To generalize the registration procedure from two images to a larger dataset, we utilize a graph theoretic approach whereby we first compute all pairwise registrations between pairs of bones that differ in length by 20% at most, while storing the obtained NCC score of each pair in a distance matrix *D*_*N*×*N*_. We compute the maximum spanning tree (MST) of the graph encoded by *D* with a preselected image serving as the root. To guarantee that the graph is connected for the extraction of the MST, we register all length-wise consecutive pairs of bones, regardless of their length differences. Last, we align all of the images to the root MST image, by aggregating the transformations along the path and connecting them over the MST graph.

### Determination of the Origin of the Longitudinal Axis

The origin of the longitudinal axis of each bone (*Z* = 0) was set at the transverse section in which the diameter of the bone collar in the average E16.5 image was minimal. This section was presumed to be the location of initial chondrocyte hypertrophy at the primary ossification center from which longitudinal growth progresses bidirectionally and, therefore, it provided a natural choice for the longitudinal origin of the bone. Positive values represent elements located proximally to the origin, whereas negative values represent distal locations. Notably, although the existence of a longitudinal origin is necessary for all further calculations, its specific location along the axis does not influence any of the obtained results.

### Identification of Stationary Elements

By definition, a stationary element is one for which the physical position remains constant over time. However, to account for random fluctuations and noise, we define the speed of drift of an element between time points *t*_0_ and *t*_1_ as the absolute change in physical position divided by the change in total bone length:

$$E_{DriftSpeed}\left(t_{0},t_{1}\right)=\frac{\left|E_{PhysicalPosition}\left(t_{1}\right)-E_{PhysicalPosition}\left(t_{0}\right)\right|}{BoneLength\left(t_{1}\right)-BoneLength\left(t_{0}\right)}$$

We also define a level of tolerance (0 < *ε* < 1) to separate between stationary (*E*_*DriftSpeed*_(*t*_0_, *t*_1_) < *ε*) and drifting elements (*ε* ≤ *E*_*DriftSpeed*_(*t*_0_, *t*_1_)). In order to set an appropriate *ε* value, we sought to identify the minimal speed of an element that is guaranteed to be drifting. To this end, we relied on the identification of drifting elements reported in [20]. We then calculated the rate of drift of these elements and the rates of proximal and distal growth based on the data extracted from the image analyses (Fig 5 and Fig 7). This analysis showed that drifting speeds range between 0.1409 (the distal end of the femoral third trochanter between days P20 and P24) and 0.5546 (the distal end of the humeral deltoid tuberosity between days P24 and P28). These results suggests 0.1409 as the drifting speed below which changes in physical position of elements are likely to result from random fluctuations or noise and, therefore, the elements should be classified as stationary. In order to further minimize the possibility of false rejection of drifting elements, we used the lower value of *ε* = 0.1, i.e., when the change in physical position of an element is less than 10% the total elongation of the bone during the specified time interval.

### Mathematical Formulation of the FP Model

Assume a bone with a total length *l* ∊ ℝ_+_, in which the physical positions of the proximal and distal ends of the bone are represented as a function of *l*: *Dist*(*l*) < 0 < *Prox*(*l*), respectively, such that: *Prox*(*l*) − *Dist*(*l*) = *l*. Note that since *Dist*(*l*) is negative and monotonically decreasing, the growth curve of the distal end is: −*Dist*(*l*).

1. We will now show the feasibility of a FP under this model: We define a transverse plane *FP* along the bone with a physical position: *Dist*(*l*) ≤ *FP*_*Physical*_(*l*) ≤ *Prox*(*l*) and a fixed relative position: *FP*_*Relative*_. From the definition of the relative position we get: $FP_{Relative}=\frac{FP_{Physical}\left(l\right)-Dist\left(l\right)}{l}$, which allows us to isolate the physical position of *FP*: *FP*_*Physical*_(*l*) = *l* ⋅ *FP*_*Relative*_ + *Dist*(*l*). The fixed plane is defined as a plane that has both constant relative position and constant physical positions, which is obtained at zero velocity of the physical position: *FP*_*Physical*_′(*l*) = *FP*_*Relative*_ + *Dist*′(*l*) = 0, which leads to: *FP*_*Relative*_ = −*Dist*′(*l*). To show that the obtained *FP*_*Relative*_ is a valid plane it is necessary to show that it is located between the two ends of the bone: 0 ≤ *FP*_*Relative*_ = −*Dist*′(*l*) ≤ 1. Since: *Prox*(*l*) − *Dist*(*l*) = *l*, we get: *Prox*′(*l*) – *Dist*′(*l*) = *l*′ = 1. In addition, since both *Prox*(*l*) and −*Dist*(*l*) are monotonically increasing, *Prox*′(*l*), –*Dist*′(*l*) ≥ 0. Taken together, we get: 0 ≤ *FP*_*Relative*_ = −*Dist*′(*l*) ≤ 1, Q.E.D.
2. We will now show that the FP is found where the ratio between its distances to the proximal and distal ends of the bone equals the ratio of growth rates at the two ends: $\frac{Prox\left(l\right)-FP_{Physical}}{FP_{Physical}-Dist\left(l\right)}=\frac{Prox\prime \left(l\right)}{-Dist\prime \left(l\right)}$. Following section (1): *FP*_*Relative*_ = −*Dist*′(*l*). Since: $FP_{Relative}=\frac{FP_{Physical}-Dist\left(l\right)}{l}$ and since: *Prox*′(*l*) − *Dist*′(*l*) = *l*′ = 1, we get: $\frac{FP_{Physical}-Dist\left(l\right)}{l}=\frac{-Dist\prime \left(l\right)}{Prox\prime \left(l\right)-Dist\prime \left(l\right)}$. In addition, since: *l* = (*Prox*(*l*) − *FP*_*Physical*_) + (*FP*_*Physical*_ − *Dist*(*l*)), we get: $\frac{FP_{Physical}-Dist\left(l\right)}{\left(Prox\left(l\right)-FP_{Physical}\right)+\left(FP_{Physical}-Dist\left(l\right)\right)}=\frac{-Dist\prime \left(l\right)}{Prox\prime \left(l\right)-Dist\prime \left(l\right)}$. By inverting both expressions we get:

$$\frac{\left(Prox\left(l\right)-FP_{Physical}\right)+\left(FP_{Physical}-Dist\left(l\right)\right)}{FP_{Physical}-Dist\left(l\right)}=\frac{Prox\prime \left(l\right)-Dist\prime \left(l\right)}{-Dist\prime \left(l\right)}\;\Rightarrow$$

$$\frac{Prox\left(l\right)-FP_{Physical}}{FP_{Physical}-Dist\left(l\right)}+1=\frac{Prox\prime \left(l\right)}{-Dist\prime \left(l\right)}+1\;\Rightarrow$$

$$\frac{Prox\left(l\right)-FP_{Physical}}{FP_{Physical}-Dist\left(l\right)}=\frac{Prox\prime \left(l\right)}{-Dist\prime \left(l\right)}\;,\;\text{Q.E.D.}$$

3. We will now show that the farther an element is located from the FP the faster it will drift in order to maintain its relative position. Let *E*_*Physical*_(*l*) be the physical position of an element *E* with a constant relative position *E*_*Relative*_. Based on (1), the rate of drifting of *E* is: $E_{Physical}^{\prime }\left(l\right)=\left|E_{Relative}+Dist\prime \left(l\right)\right|=\left|E_{Relative}-FP_{Relative}+\left(FP_{Relative}+Dist\prime \left(l\right)\right)\right|$. According to (1): *FP*_*Relative*_ + *Dist*′(*l*) = 0, which leads to: $E_{Physical}^{\prime }\left(l\right)=\left|E_{Relative}-FP_{Relative}\right|$. We got that the rate of drifting of an element is equal to the distance between the relative positions of the element and the FP, and therefore the farther an element is located from the FP the faster it will drift, Q.E.D.
4. We will now show that elements that do not overlap with the FP drift away from the FP in order to compensate for the change in relative position. Let *E*_*Physical*_(*l*) be the physical position of an element *E* with a constant relative position *E*_*Relative*_. According to (1): *E*_*Physical*_(*l*) = *l* ⋅ *E*_*Relative*_ + *Dist*(*l*), and also: *FP*_*Physical*_ = *l* ⋅ *FP*_*Relative*_ + *Dist*(*l*), the distance between *E* and the FP is: |*E*_*Physical*_(*l*) − *FP*_*Physical*_| = |*l* ⋅ *E*_*Relative*_ + *Dist*(*l*) − *l* ⋅ *FP*_*Relative*_ − *Dist*(*l*)| = |*l*(*E*_*Relative*_ − *FP*_*Relative*_)|. As: *E*_*Relative*_ − *FP*_*Relative*_ is a constant expression, when the element and the FP do not overlap (*E*_*Relative*_ ≠ *FP*_*Relative*_) the physical distance between an element and the FP increases with the length of the bone (*l*), Q.E.D.
5. We will now show that preservation of the relative position of an element over time necessitates that the balance between proximal and distal growth remains constant. For an element with a fixed relative position: *E*_*Relative*_ = *FP*_*Relative*_, and according to (1): *E*_*Relative*_ = −*Dist*′(*l*). Since *E*_*Relative*_ is constant, we get that the growth function of the distal end (−*Dist*′(*l*)) must also be constant. Since based on (1): *Prox*′(*l*) = 1 + *Dist*′(*l*), we get that the growth function of the proximal end (*Prox*′(*l*)) must be constant as well, and thus the growth balance: $\frac{Prox\prime \left(l\right)}{-Dist\prime \left(l\right)}$ is constant. Q.E.D.

### Calculation of the Relative and Physical Positions of the Fixed Plane

According to our statistical regression analysis, during each time period in which a stationary element has been identified a linear line provided a good fit to the data points. Therefore, the total elongation of the distal end (*Dist*) as a function of the total length of the bone (*l*) can be expressed as: *Dist*(*l*) = *a* ⋅ *l* + *b*, where *a* and *b* are the slope and intercept of the line, respectively. Since growth is represented as a function of total bone length, the rate of elongation of the entire bone is constantly 1. In addition, the rate of elongation of the distal end is *a* and, therefore, the relative contribution of the distal end to the elongation of the bone is constantly $\frac{a}{1}=a$, which by definition is equal to the relative position of the FP: *FP*_*Relative*_ = *a*. Taken together, we get that: *Dist*(*l*) = *FP*_*Relative*_ ⋅ *l* + *b*, thus allowing us to extract the relative position of the FP from the parameters obtained by the regression.

By definition, the physical position of the FP is: *FP*_*Physical*_ = −*Dist*(*l*) + *FP*_*Relative*_ ⋅ *l* = −*b*, which leads to: *Dist*(*l*) = *FP*_*Relative*_ ⋅ *l* − *FP*_*Physical*_, thus allowing us to extract the physical position of the FP as well.

### Calculation of Optimal Growth Balance for Minimum Element Drifting Activity

Assume a bone with *n* symmetry-breaking elements located at constant relative positions: $E_{Relative}^{i}\;\left(i=1,\ldots ,n\right)$, such that $E_{Relative}^{i}\leq E_{Relative}^{i+1}\;\forall i\in \left\{1,\ldots ,n-1\right\}$, and a FP with a relative position *FP*_*Relative*_ ∊ [0, 1]. We define a loss function that quantifies the sum of the rates of drift over all *n* elements, and we search for the relative position of the FP that minimizes the loss: $\underset{FP_{Relative}\in \left[0,1\right]}{\text{arg}\;min}\sum_{i=1}^{n}\left|E_{Physical}^{i}\left(l\right)\prime \right|$. According to the FP model: $E_{Physical}^{i}\left(l\right)\prime =E_{Relative}^{i}-FP_{Relative}$ and therefore: $\sum_{i=1}^{n}\left|E_{Physical}^{i}\left(l\right)\prime \right|=\sum_{i=1}^{n}\left|E_{Relative}^{i}-FP_{Relative}\right|$, which is the sum of distances of all elements from the FP in terms of relative positions. It can be shown that for $n\in {\mathbb{N}}_{odd}:\;\underset{FP_{Relative}\in \left[0,1\right]}{\text{arg}\;min}=median\left\{E_{Relative}^{i}\right\}$ and for $n\in {\mathbb{N}}_{even}:\;\underset{FP_{Relative}\in \left[0,1\right]}{\text{arg}\;min}=\left\{x|E_{Relative}^{\frac{n}{2}}\leq x\leq E_{Relative}^{\frac{n}{2}+1}\right\}$. That is to say, when there are an odd number of elements, the optimal relative position of the FP overlaps that of the middle element along the bone, whereas when the number of elements is even, any relative position between the two middle elements is optimal.

Based on this mathematical term, to calculate the optimal relative position of the FP through the development of each bone we first calculated the average relative position of each element. Then, for each value of total bone length $\underset{FP_{Relative}\in \left[0,1\right]}{\text{arg}\;min}$ was calculated, while considering only ossified elements.

### Statistical Methods

Statistical analysis was performed using the R language for statistical computing [81]. Regression analysis for linearity of growth data was performed using ordinary least square and evaluated based on scatterplots, Pearson’s correlation and *p*-values of the t-scores.

The statistical significance of the proximity between FPs and symmetry-breaking elements was determined by permutation analysis. The null hypothesis was that the distribution of FP positions is not affected by the positions of elements and, therefore, the average distance of a FP from all elements would not have changed if it had been located on another bone. First, we measured for all FPs on all bones the distances to all elements, then calculated the average distance for each bone and, finally, the average distance for all bones. Then, the FPs and the elements of the bones were mixed and permutation distribution was calculated by a one- (left-) sided test. Significant difference (*p*-value < 0.01) would imply rejection of the null hypothesis.

### Hardware and External Software

All computations were conducted on an Intel Core i7-3930K CPU at 3.2 GHz with 16 GB of RAM, with Windows 7 64-bit operating system and on Matlab platform (MatLab and Image Processing toolbox R2014a (8.3.0.532) 64-bit, The MathWorks, Inc., Natick, Massachusetts, United States). The volume-based registration module for rigid image registration algorithm is from the freely available Image Registration Toolkit (IRTK), used under license from Ixico Ltd [25–27]. Manual image registration for the assessment of preservation of mineralized structures and for spatial standardization of all in vivo images was performed using the free and open-source software package 3DSlicer v. 3.6. Visualization of 3D isosurface of micro-CT images was performed using the free software MicroView 2.1.2 (GE Healthcare).

## Supporting Information

S1 Data Data for Fig 2. (XLSX)

S2 Data Data for Fig 6. (XLSX)

S3 Data Data for Fig 8. (XLSX)

S4 Data Data for Fig 9 and Fig 10. (XLSX)

S1 Fig Illustration of the relative position of each element during development as measured in 3D micro-CT images of the humerus (corresponds to the graph of the humerus shown in Fig 2). The length of all bones is standardized and the position of each element is shown relative to the distal (0%) and the proximal (100%) ends of the bone. Gray regions in early bone images represent cartilaginous tissue; hollow circles mark elements residing either proximally to the proximal growth plate or distally to the distal growth plate, whereas filled circles mark elements residing between the growth plates. Transition of an element from outside to between the growth plates, such as of the lateral epicondyle, indicates ossification of the element by the advancing growth plate.
(TIF)

S2 Fig Illustration of the longitudinal origin of the bone. The longitudinal origin of the bone is shown in a frontal slice of an E16.5 humerus micro-CT scan. The origin of the longitudinal axis of each bone (*Physical position* = 0) was set at the transverse section in which the diameter of the bone collar (highlighted in red) is minimal. This section is presumed to be the location of initial chondrocyte hypertrophy at the primary ossification center, from which longitudinal growth progresses bidirectionally. Positive values represent elements located proximally to the origin, whereas negative values represent distal locations.
(TIF)

S1 File Quantitative experimental evaluation of the proposed image registration scheme. To validate the fidelity of the algorithm, we analyze four fundamental attributes of its performance: (a) the accuracy of pairwise registrations, (b) the accuracy, precision and sensitivity of the NCC score as a binary classifier for the quality of pairwise registrations, (c) the accuracy of agglomerating pair alignments to multi-image alignments, and (d) the sensitivity of longitudinal position measurements to inaccuracies in image registration.
(DOCX)

S2 File Illustration of the masked regions of each bone type. For each type of long bone at each developmental day between E16.5 and P6, two orthogonal longitudinal slices and three transverse slices of a micro-CT scan are presented with the zeroed-out regions highlighted in red. Due to the minimal amount of trabecular bone and cortical thickening, fibulae were registered without prior masking.
(PDF)

## Abbreviations:

3D: three-dimensional
E: embryonic day
P: postnatal day
FP: fixed plane
CT: computed tomography
NCC: normalized cross correlation
MST: maximum spanning tree
FGF: fibroblast growth factor
IHH: Indian hedgehog
BMP: bone morphogenetic protein
PBS: phosphate-buffered saline
PCR: polymerase chain reaction
PFA: paraformaldehyde
HA: hydroxyapatite
Q.E.D: quod erat demonstrandum
