# Supplementary figures and images for: Isometric Scaling in Developing Long Bones Is Achieved by an Optimal Epiphyseal Growth Balance
Source: PLoS Biol. 2015 Aug 4;13(8):e1002212. doi: 10.1371/journal.pbio.1002212 (PMC4524611; doi:10.1371/journal.pbio.1002212)

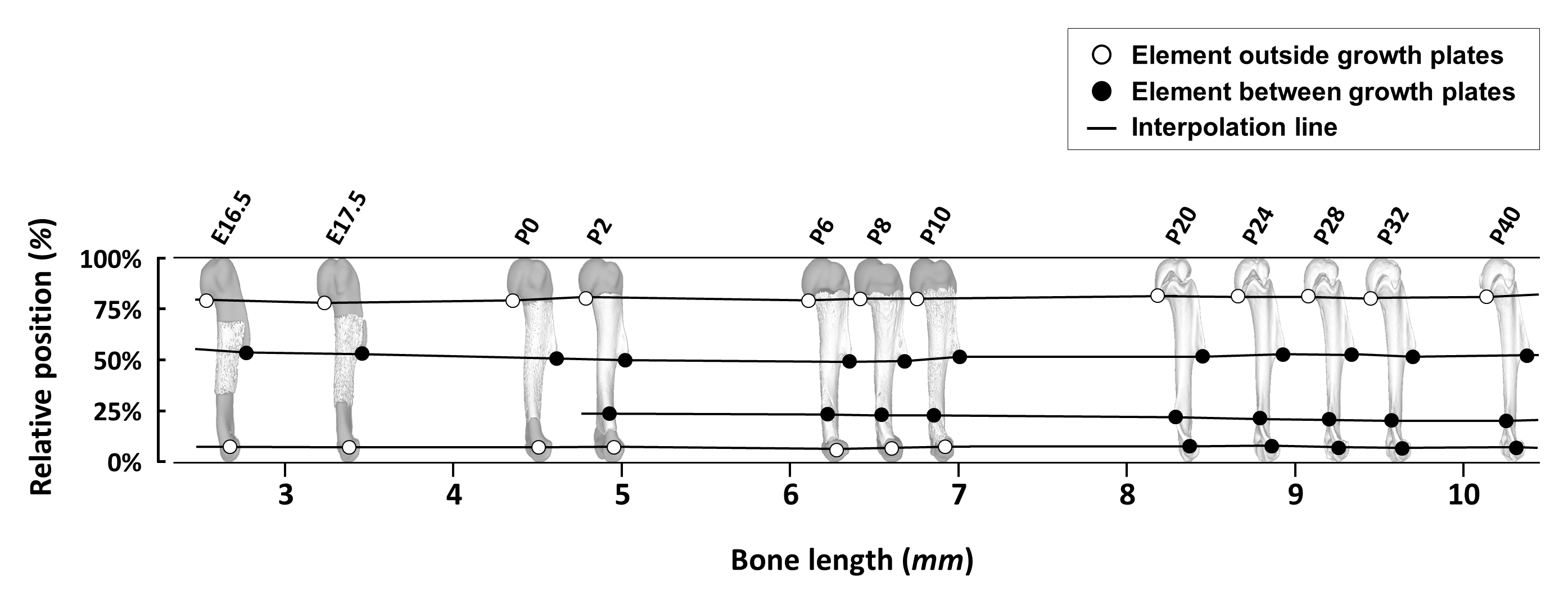

Supplement: S1 Fig — The length of all bones is standardized and the position of each element is shown relative to the distal (0%) and the proximal (100%) ends of the bone. Gray regions in early bone images represent cartilaginous tissue; hollow circles mark elements residing either proximally to the proximal growth plate or distally to the distal growth plate, whereas filled circles mark elements residing between the growth plates. Transition of an element from outside to between the growth plates, such as of the lateral epicondyle, indicates ossification of the element by the advancing growth plate. (TIF) [file pbio.1002212.s005.tif]

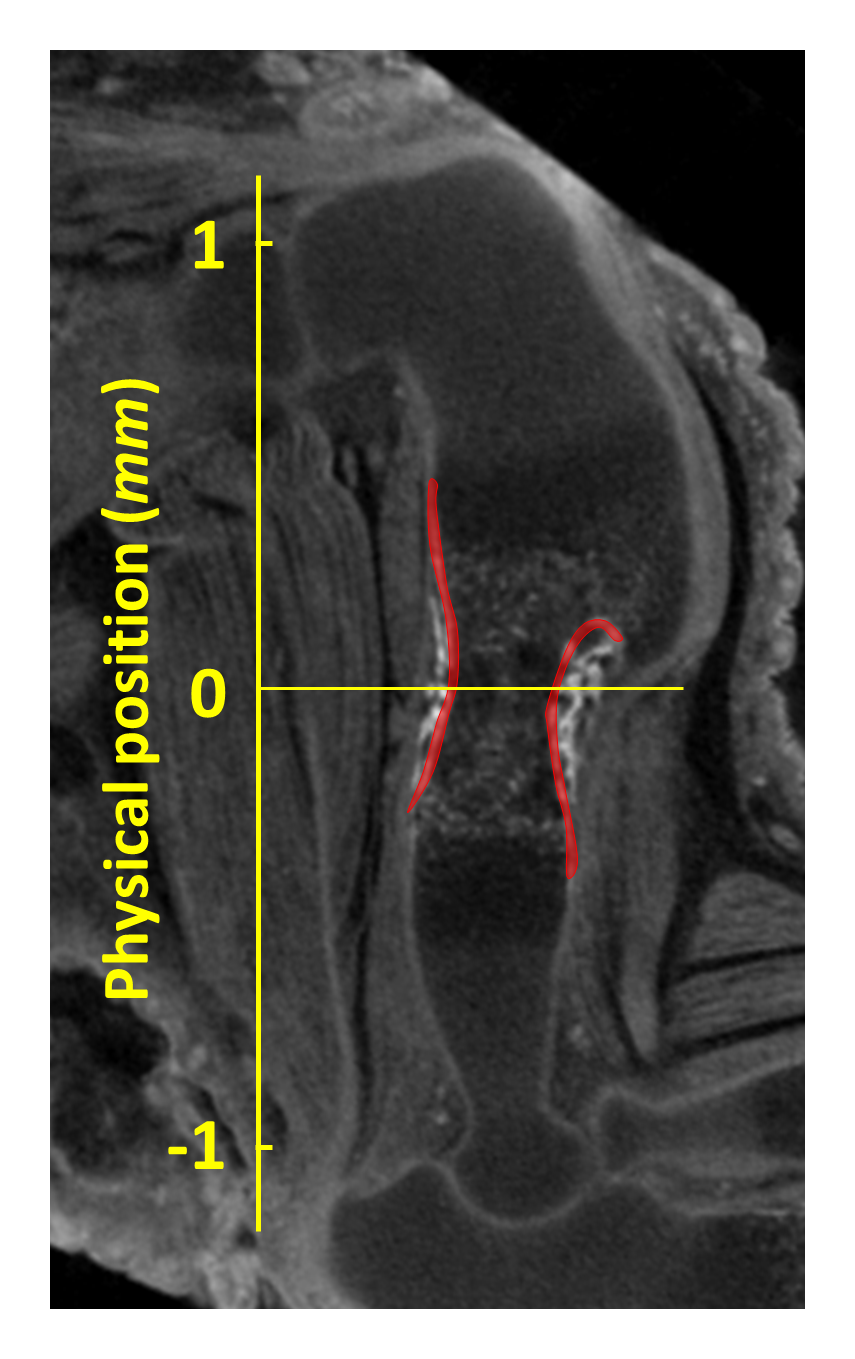

Supplement: S2 Fig — The longitudinal origin of the bone is shown in a frontal slice of an E16.5 humerus micro-CT scan. The origin of the longitudinal axis of each bone (Physical position = 0) was set at the transverse section in which the diameter of the bone collar (highlighted in red) is minimal. This section is presumed to be the location of initial chondrocyte hypertrophy at the primary ossification center, from which longitudinal growth progresses bidirectionally. Positive values represent elements located proximally to the origin, whereas negative values represent distal locations. (TIF) [file pbio.1002212.s006.tif]
