# Supplementary material for: Isometric Scaling in Developing Long Bones Is Achieved by an Optimal Epiphyseal Growth Balance
Source: PLoS Biol. 2015 Aug 4;13(8):e1002212. doi: 10.1371/journal.pbio.1002212 (PMC4524611; doi:10.1371/journal.pbio.1002212)
Supplement: S1 File — To validate the fidelity of the algorithm, we analyze four fundamental attributes of its performance: (a) the accuracy of pairwise registrations, (b) the accuracy, precision and sensitivity of the NCC score as a binary classifier for the quality of pairwise registrations, (c) the accuracy of agglomerating pair alignments to multi-image alignments, and (d) the sensitivity of longitudinal position measurements to inaccuracies in image registration. (DOCX) [file pbio.1002212.s007.docx]

**Quantitative experimental evaluation of the proposed image registration scheme**

**1. Evaluation of the registration scheme accuracy**

We analyzed a dataset consisting of 163 micro-CT images. Because the input of the algorithm comprised of bone images of different mice at potentially different developmental stages, there was no objective groundtruth for evaluating the matching accuracy. Therefore, a control group of 52 images, ranging over all ages, was registered manually by an experienced operator to compute the groundtruth for the algorithm's results. The remaining 111 images served as ‘error accumulators’ as a means to challenge the MST generalization scheme with large datasets.

The pairwise registration accuracy is quantified by computing the residual pairwise transformation $\mathbf{A}_{\mathbf{err}}^{\mathbf{s},\mathbf{t}}$ of matching a target image $\left( \mathbf{I}^{\mathbf{t}} \right)$ to a source image $\left( \mathbf{I}^{\mathbf{s}} \right)$:

$$\mathbf{A}_{\mathbf{err}}^{\mathbf{s},\mathbf{t}}=\left( \mathbf{A}_{\mathbf{algorithm}}^{\mathbf{s},\mathbf{t}} \right)^{-\mathbf{1}}\cdot\mathbf{A}_{\mathbf{manual}}^{\mathbf{s},\mathbf{t}}$$

where $\mathbf{A}_{\mathbf{algorithm}}^{\mathbf{s},\mathbf{t}}\mathbb{\in R}^{\mathbf{4}\times\mathbf{4}\mathrm{QUOTE}}$ is the pairwise transformation estimated by the proposed scheme, and:

$$\mathbf{A}_{\mathbf{manual}}^{\mathbf{s},\mathbf{t}}=\left( \mathbf{A}_{\mathbf{manual}}^{\mathbf{s}} \right)^{-\mathbf{1}}\cdot\mathbf{A}_{\mathbf{manual}}^{\mathbf{t}}$$

is the groundtruth pairwise transform computed manually. All transformations are represented in 3D homogenous coordinates in $\mathbb{R}^{\mathbf{4}\times\mathbf{4}\mathrm{QUOTE}}$.

The residual registration matrix $\mathbf{A}_{\mathbf{err}}^{\mathbf{s},\mathbf{t}}$ is used to compute registration accuracy attributes. Let $\mathbf{r}_{\mathbf{3}\times\mathbf{3}}$ and $\mathbf{t}_{\mathbf{3}\times\mathbf{1}}$ be the corresponding rotation and translation components of $\mathbf{A}_{\mathbf{err}}^{\mathbf{s},\mathbf{t}}$, respectively, and let $\mathbf{p}_{\mathbf{i}}=\left[ \mathbf{x},\mathbf{y},\mathbf{z},\mathbf{1} \right]^{\mathbf{T}}$ be the homogenous coordinates vector of a foreground voxel in $\mathbf{I}^{\mathbf{t}}$, i.e. within the used salient feature, we consider the following image registration accuracy attributes:

1. Euler angle error (EAE; in degrees): $\cos^{-1} \left( 0.5\cdot tr\left( r \right)^{-1} \right)$
2. Translation error (TE; in units of voxels): $\left\| \mathbf{t} \right\|$
3. Normalized TE (NormTE; in units of %): $\frac{\left\| \mathbf{t} \right\|}{\mathbf{Length}\left( \mathbf{I}^{\mathbf{t}} \right)}\times100$
4. Mean voxel displacement error (MVDE; in units of voxels): $\bar{\left\| \mathbf{p}_{\mathbf{i}}-\mathbf{A}_{\mathbf{err}}^{\mathbf{s},\mathbf{t}}{\cdot\mathbf{p}}_{\mathbf{i}} \right\|}$
5. Normalized MVDE (NormMVDE; in %): $\frac{\bar{\left\| \mathbf{p}_{\mathbf{i}}-\mathbf{A}_{\mathbf{err}}^{\mathbf{s},\mathbf{t}}{\cdot\mathbf{p}}_{\mathbf{i}} \right\|}}{\mathbf{Length}\left( \mathbf{I}^{\mathbf{t}} \right)}\times100$

For each parameter, we define a tolerance threshold for the error level above which a pairwise registration is considered erroneous. These values were set by manually detecting the success/failure of pairwise registrations, and extracting the threshold levels that best discriminate the two groups:

- Euler angle error (EAE) ≤ 5.36⁰
- Translation error (TE) ≤ 5.76 µm
- Normalized translation error (NormTE) ≤ 1.8
- Voxel displacement error (VDE) ≤ 6.2 µm
- Normalized voxel displacement error (NormVDE) ≤ 1.9

**1.1 Evaluation of pairwise registration accuracy**

Following the execution of the algorithm on all 163 images, we evaluated the accuracy of the pairwise registrations. The control group provided 52 pairs in which the groundtruth was available for both images. Out of the 52 pairs, 4 pairs were classified as misaligned by the algorithm for having an NCC score of less than 0.7 and were therefore excluded from this analysis. To evaluate the quality of each pairwise registration of the 48 remaining pairs, we calculated all five parameters. The results are summarized in the following table:

|  | **EAE** | **TE** | **NormTE** | **VDE** | **NormVDE** |
| --- | --- | --- | --- | --- | --- |
| **Target and source bones from the same age**  **(mean±SD; *n*=26)** | 2.09±1.54 | 2.92±1.32 | 0.84±0.55 | 3.35±1.28 | 0.96±0.56 |
| **Target and source bones from different ages**  **(mean±SD; *n*=22)** | 2.55±2.21 | 3.04±1.41 | 0.79±0.39 | 3.49±1.34 | 0.91±0.41 |

In order to evaluate the success rate implied by these results, we computed for each parameter the fraction of registrations that were lower than the corresponding tolerance threshold. The results are summarized in the following table:

|  | **EAE** | **TE** | **NormTE** | **VDE** | **NormVDE** |
| --- | --- | --- | --- | --- | --- |
| **Target and source bones from the same age (*n*=26)** | 26/26=1 | 26/26=1 | 24/26=0.92 | 26/26=1 | 24/26=0.92 |
| **Target and source bones from different ages (*n*=22)** | 21/22=0.95 | 21/22=0.95 | 22/22=1 | 21/22=0.95 | 22/22=0.95 |

It follows that the accuracy of pairwise registration of bones from the same developmental day and from different developmental days (i.e., with significantly different morphologies) is well within a tolerable range.

**1.2. Evaluation of the registration agglomeration**

We next evaluated the error accumulated by the MST registration agglomeration scheme. For that, we compared the final, agglomerated transformation of each image belonging to the control group with the corresponding groundtruth transformation and calculated all five quality parameters. The results are summarized in the following table:

|  | **EAE** | **TE** | **NormTE** | **VDE** | **NormVDE** |
| --- | --- | --- | --- | --- | --- |
| **E16.5 (mean±SD); *n*=4** | 2.03±1.00 | 2.92±1.78 | 1.64±0.96 | 3.33±1.71 | 1.85±0.92 |
| **E17.5 (mean±SD); *n*=8** | 5.21±1.98 | 2.40±0.84 | 0.97±0.46 | 3.34±0.71 | 1.32±0.48 |
| **E18.5 (mean±SD); *n*=7** | 4.10±2.22 | 4.61±3.30 | 1.32±1.03 | 5.31±3.03 | 1.53±0.95 |
| **P1 (mean±SD); *n*=7** | 5.27±1.91 | 4.12±1.62 | 1.07±0.43 | 5.37±0.94 | 1.42±0.30 |
| **P2 (mean±SD); *n*=7** | 5.00±2.99 | 6.32±2.13 | 1.51±0.56 | 7.41±1.93 | 1.77±0.48 |
| **P4 (mean±SD); *n*=8** | 4.53±2.89 | 6.51±2.43 | 1.24±0.44 | 8.19±2.06 | 1.56±0.35 |
| **P6 (mean±SD); *n*=7** | 6.41±4.60 | 7.95±1.51 | 1.29±0.26 | 10.30±2.55 | 1.66±0.40 |

It follows that the EAE, TE and VDE error levels increase with the age of the mouse. This can be explained by the fact that the root of the MST is always the shortest E16.5 bone. Therefore, the older a bone is, the longer its path on the MST to the root and, correspondingly, the number of agglomerated pairwise transformations. However, it is also clear that when these values are normalized by the length of the bone, namely in NormTE and NormVDE, the error levels become relatively similar over the different ages, and the values are well within a tolerable range. This result indicates the low significance of accumulated errors to longitudinal position measurements over all ages and, therefore, the high reliability of the algorithm.

**2. Evaluation of the NCC score as a binary classifier for the accuracy of pairwise registrations**

The registration process can be performed fully automatically and without supervision by applying the registration algorithm. As the MST-based generalization to multiple images is applied by concatenation (i.e. matrix multiplication) of pairwise transformations, misalignments between pairs of images might be transferred to other pairs, and the accumulated errors might distort the overall results obtained by downstream analyses. Therefore, accuracy control of pairwise registrations is of high importance.

As the NCC score reflects the degree of similarity between the registered objects, we used the NCC score obtained from each pairwise registration as a classifier for the quality of the match. We found that the NCC score that best separates between success and failure with respect to all five parameters is 0.7 (i.e. NCC ≥ 0.7 indicates an accurate alignment and NCC < 0.7 is failure). To evaluate the quality of the proposed NCC classifier, we computed its accuracy, precision and sensitivity with respect to each of the five parameters. Out of 52 automated pairwise registrations, 4 were classified by the algorithm as failure and 48 as success. The results are shown in the following table:

|  | **EAE** | **TE** | **NormTE** | **VDE** | **NormVDE** |
| --- | --- | --- | --- | --- | --- |
| **Accuracy** | 0.94 | 0.96 | 0.94 | 0.96 | 0.94 |
| **Precision** | 0.98 | 0.98 | 0.96 | 0.98 | 0.96 |
| **Sensitivity** | 0.96 | 0.98 | 0.98 | 0.98 | 0.98 |

It follows that the NCC score provides a highly accurate, precise, and sensitive classifier for the performance of the pairwise registration algorithm.

**3. Sensitivity of longitudinal position measurements to image registration accuracy**

To assess the accuracy of the registration procedure in recovering the correct longitudinal position of loci along the bone, we calculated the error encountered by the algorithm as compared with manual rigid registration as the gold standard. For that, we documented the longitudinal positions of all symmetry breaking elements and the two ends of all 48 aforementioned control images (total number of documented sites=235). Then, we calculated the error in longitudinal position of each site by applying the error transformation of that bone on the coordinates vector of the documented site: $\left[ x,y,z,1 \right]\times\mathbf{A}_{\mathbf{err}}^{\mathbf{s},\mathbf{t}}=[x^{'},y^{'},z^{'},1]$. In this formulation, the absolute error in longitudinal position of this site is: $\left| {z-z}^{'} \right|$.

According to our analysis, the average absolute error in longitudinal position over all documented sites was 22.7 (±16.0) µm, with a maximum absolute error of 83.2 µm. Normalization of the measured error in longitudinal position of each site with the total length of the bone showed that the average absolute error was 0.55% (±0.44) of the total length of the bone, with a maximum error of 2.57%. Taken together, these results demonstrate the high fidelity of the registration algorithm in recovering the longitudinal position of sites along the bone. Moreover, as the registration agglomeration (pairs to multiple images) involves the concatenation of several pairwise transformations, these results also exemplify the low level of accumulated errors in longitudinal position.
