# Supplementary material for: Isometric Scaling in Developing Long Bones Is Achieved by an Optimal Epiphyseal Growth Balance
Source: PLoS Biol. 2015 Aug 4;13(8):e1002212. doi: 10.1371/journal.pbio.1002212 (PMC4524611; doi:10.1371/journal.pbio.1002212)
Supplement: S2 File — For each type of long bone at each developmental day between E16.5 and P6, two orthogonal longitudinal slices and three transverse slices of a micro-CT scan are presented with the zeroed-out regions highlighted in red. Due to the minimal amount of trabecular bone and cortical thickening, fibulae were registered without prior masking. (PDF) [file pbio.1002212.s008.pdf]

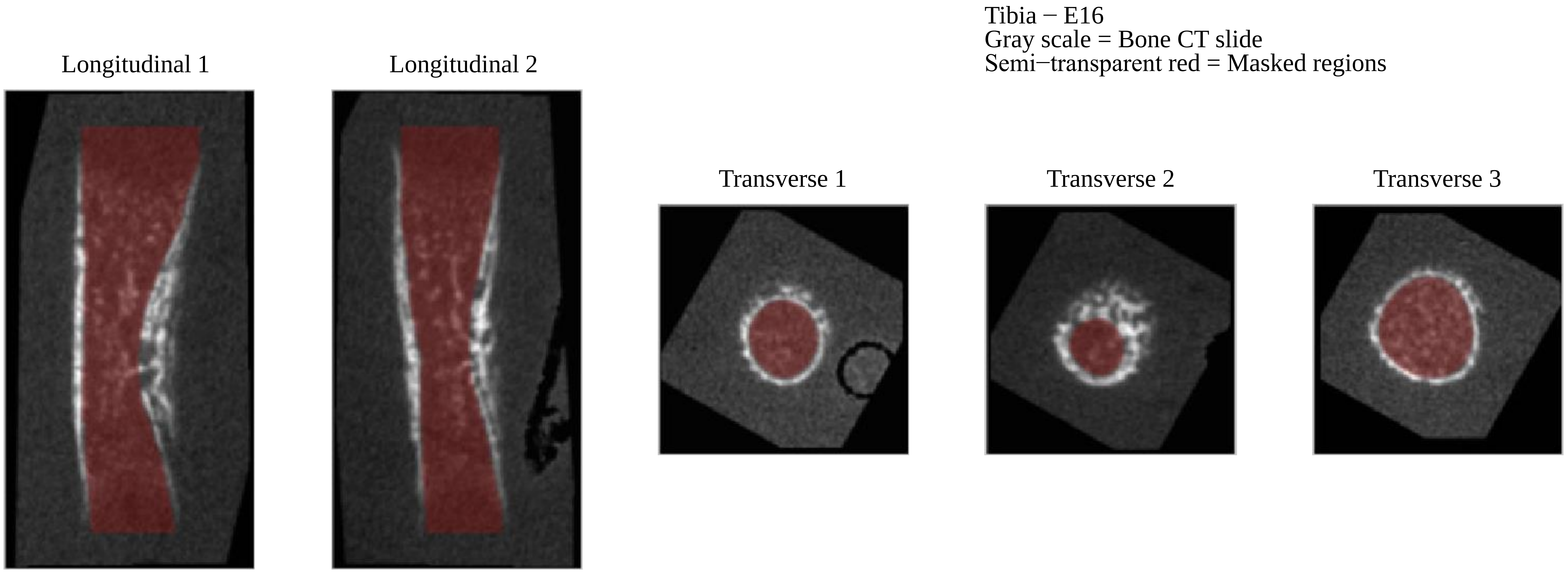
Tibia − E16
Gray scale = Bone CT slide
Semi−transparent red = Masked regions
Longitudinal 1
Longitudinal 2
Transverse 1
Transverse 2
Transverse 3

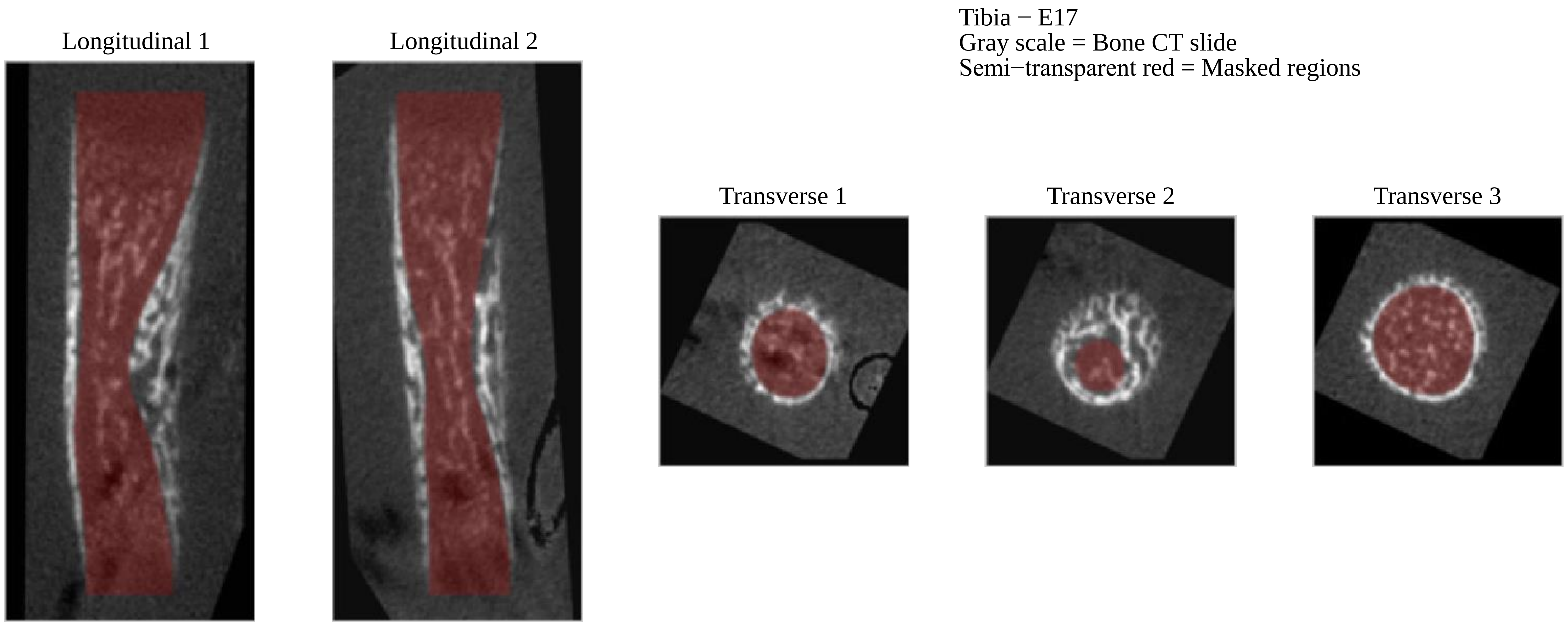
Tibia − E17
Gray scale = Bone CT slide
Semi−transparent red = Masked regions
Longitudinal 1
Longitudinal 2
Transverse 1
Transverse 2
Transverse 3

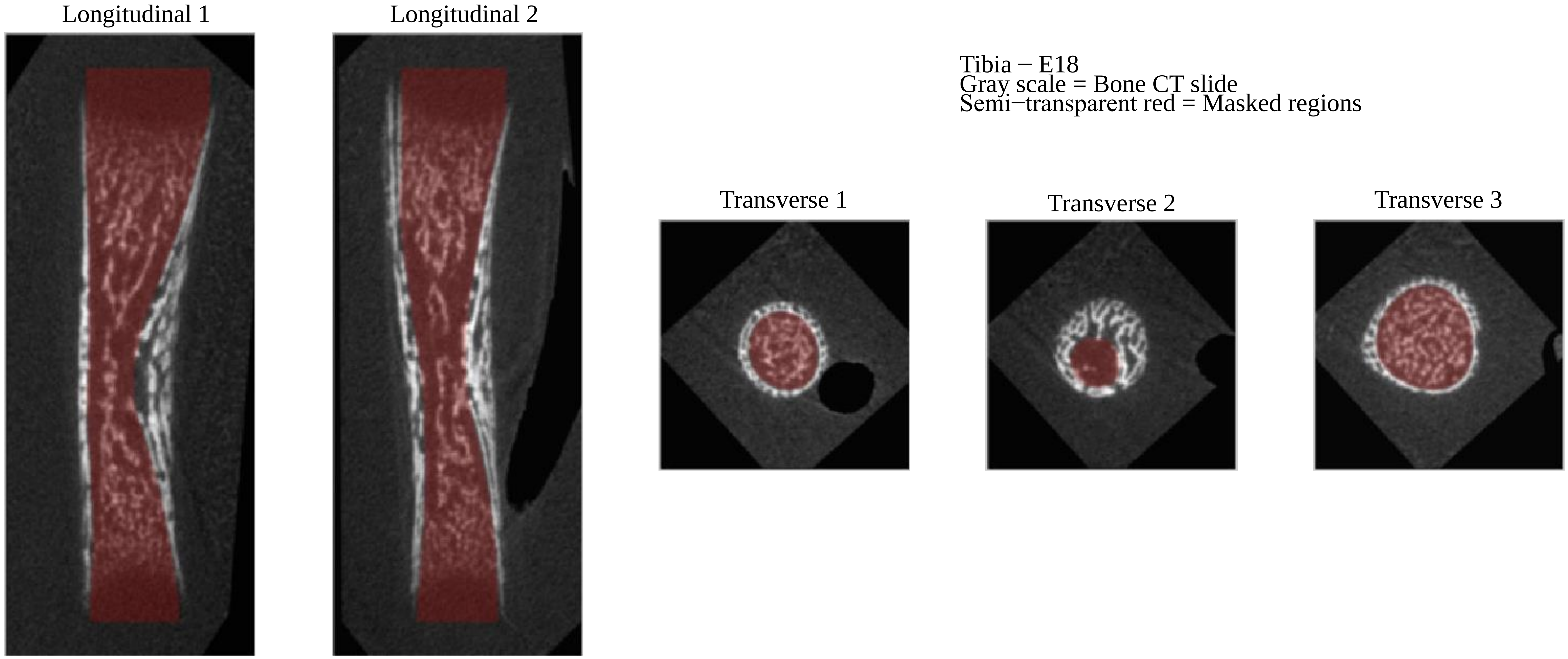
Longitudinal 1
Longitudinal 2
Tibia − E18
Gray scale = Bone CT slide
Semi−transparent red = Masked regions
Transverse 1
Transverse 2
Transverse 3

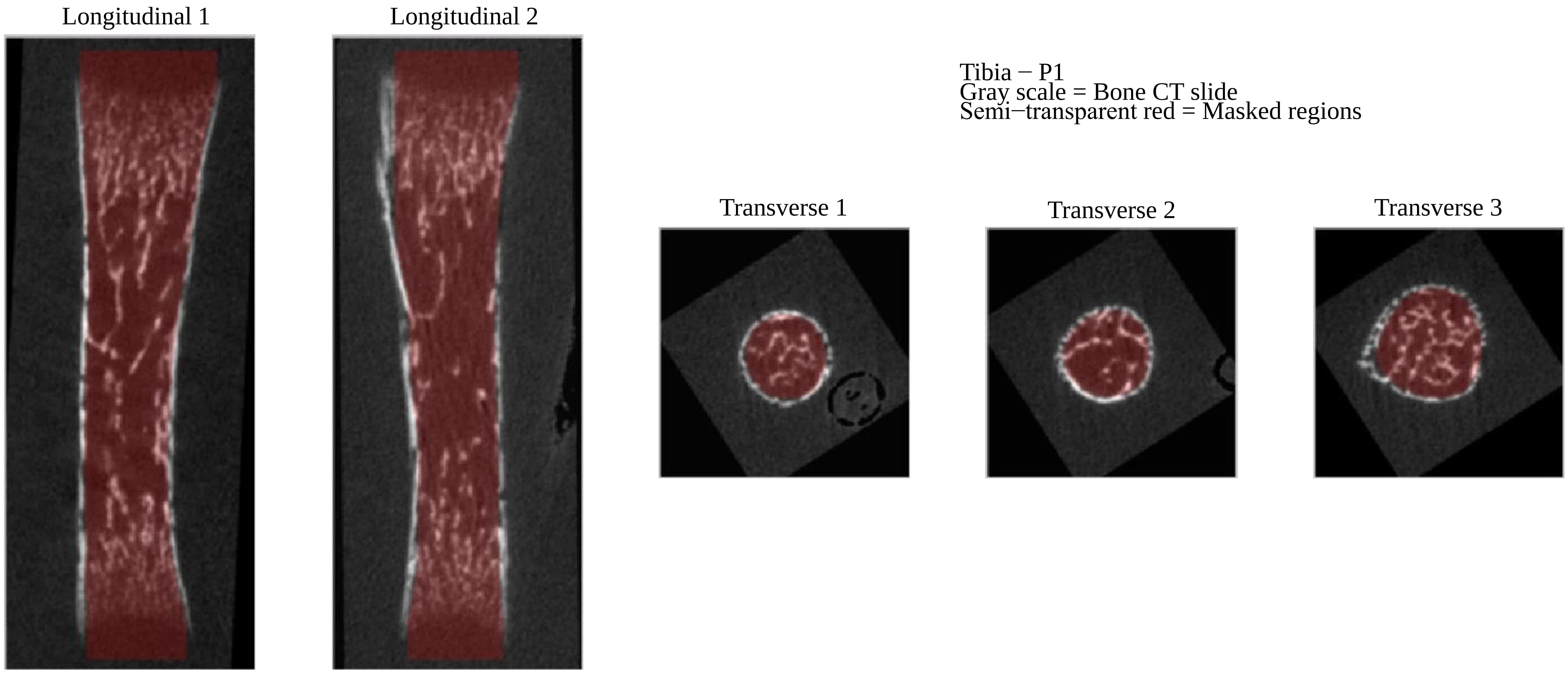
Longitudinal 1
Longitudinal 2
Tibia − P1
Gray scale = Bone CT slide
Semi−transparent red = Masked regions
Transverse 1
Transverse 2
Transverse 3

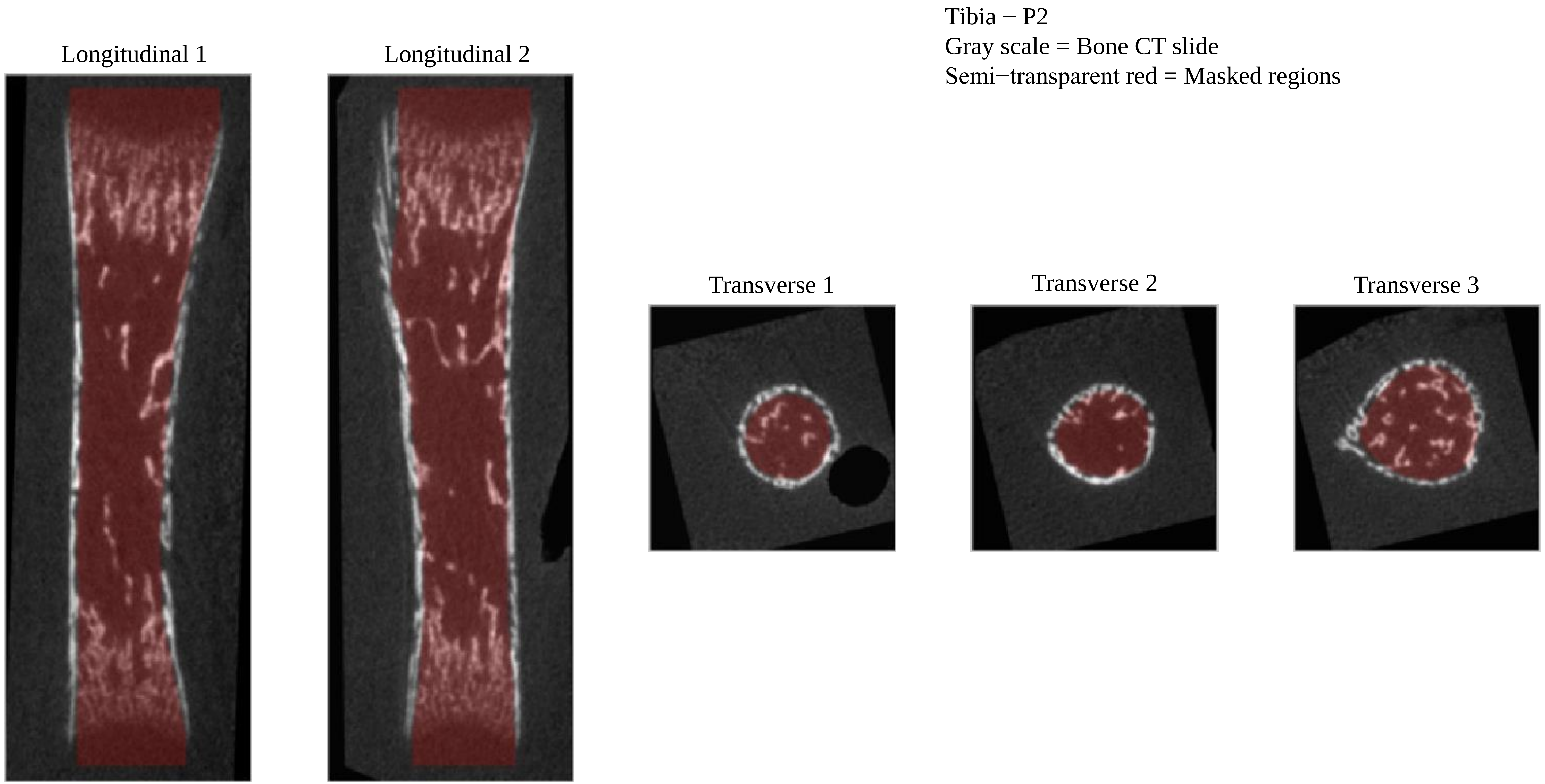
Tibia − P2
Gray scale = Bone CT slide
Semi−transparent red = Masked regions
Longitudinal 1
Longitudinal 2
Transverse 1
Transverse 2
Transverse 3

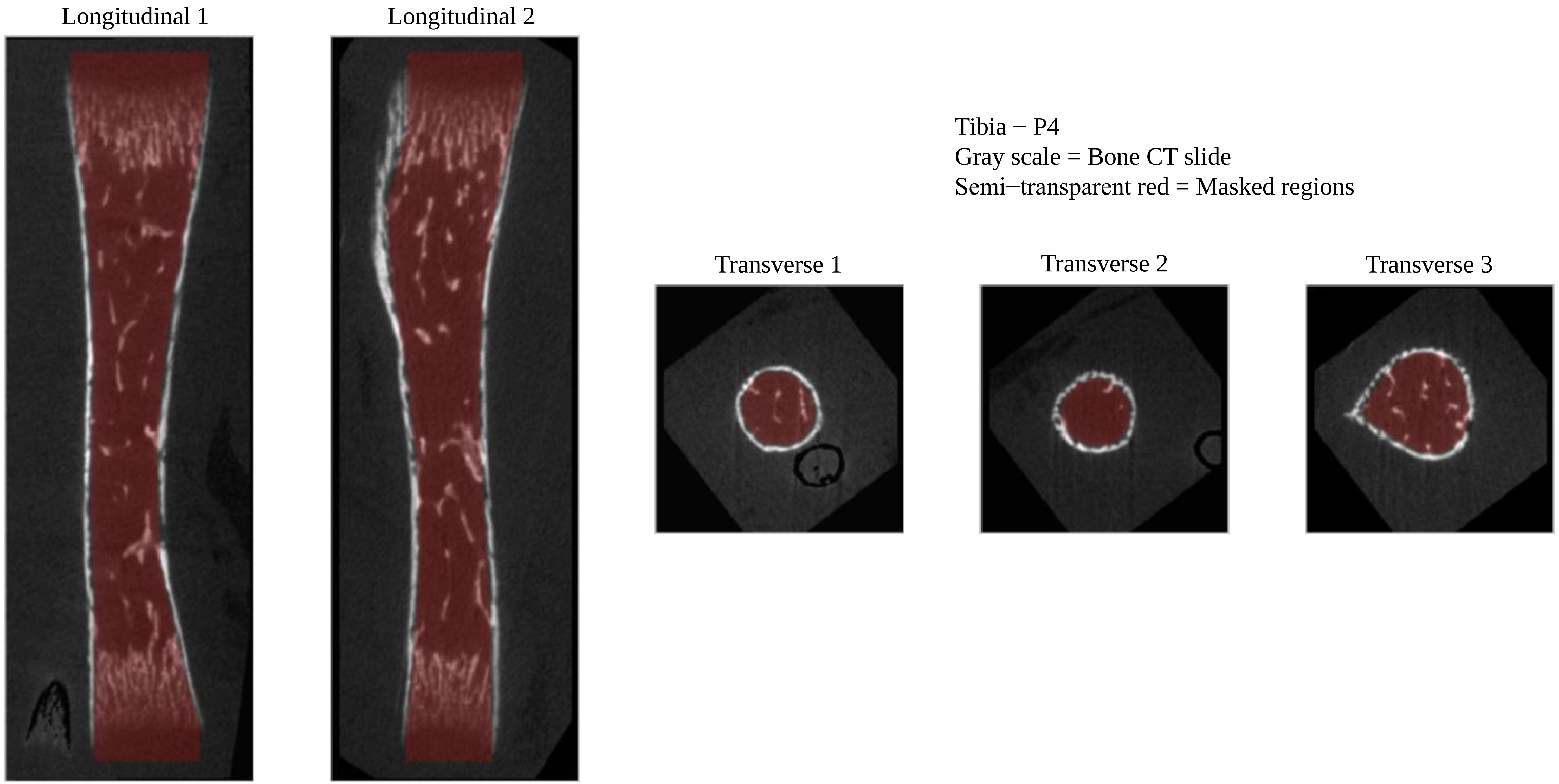
Longitudinal 1
Longitudinal 2
Tibia − P4
Gray scale = Bone CT slide
Semi−transparent red = Masked regions
Transverse 1
Transverse 2
Transverse 3

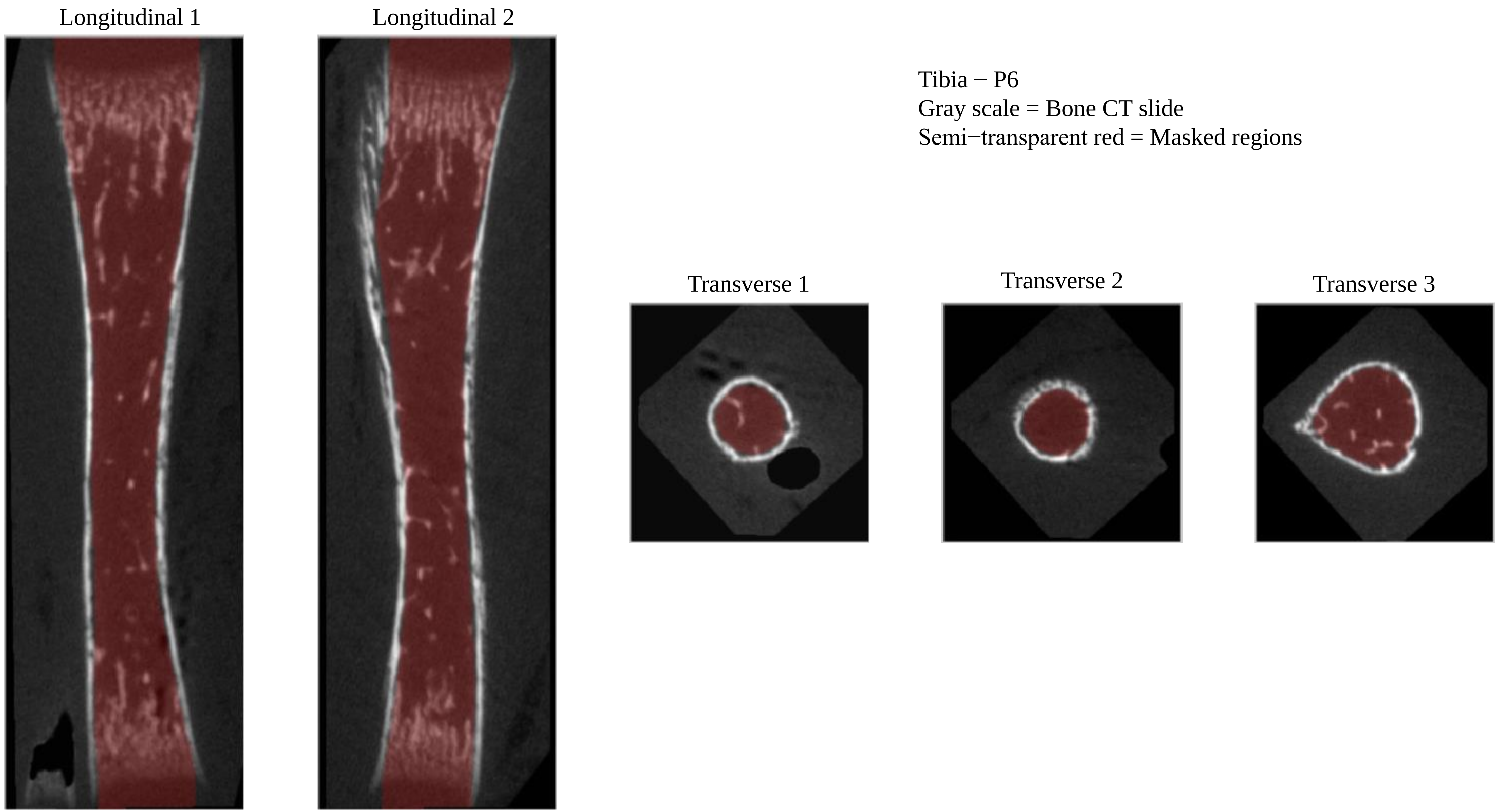
Longitudinal 1
Longitudinal 2
Tibia − P6
Gray scale = Bone CT slide
Semi−transparent red = Masked regions
Transverse 1
Transverse 2
Transverse 3

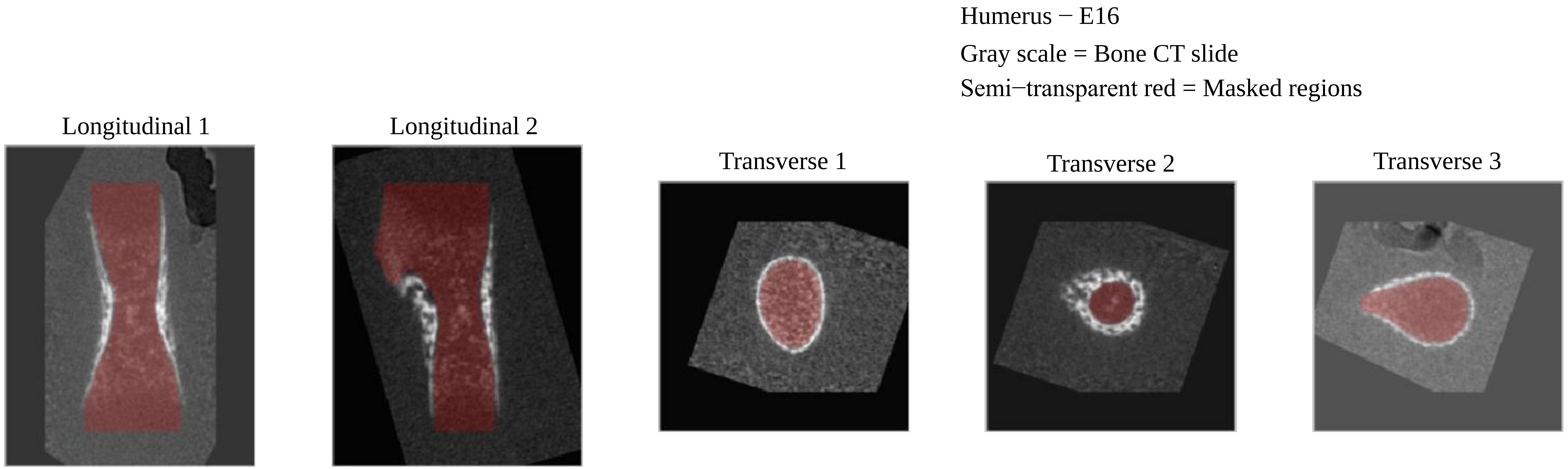
Humerus − E16
Gray scale = Bone CT slide
Semi−transparent red = Masked regions
Longitudinal 1
Longitudinal 2
Transverse 1
Transverse 2
Transverse 3

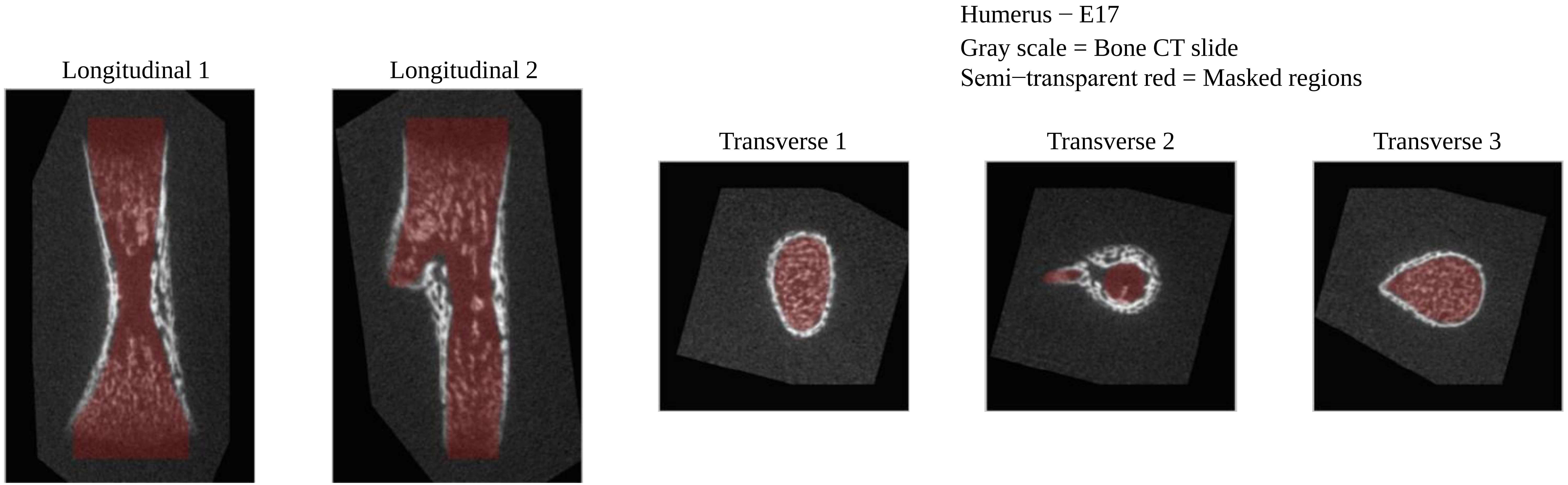
Humerus − E17
Gray scale = Bone CT slide
Semi−transparent red = Masked regions
Longitudinal 1
Longitudinal 2
Transverse 1
Transverse 2
Transverse 3

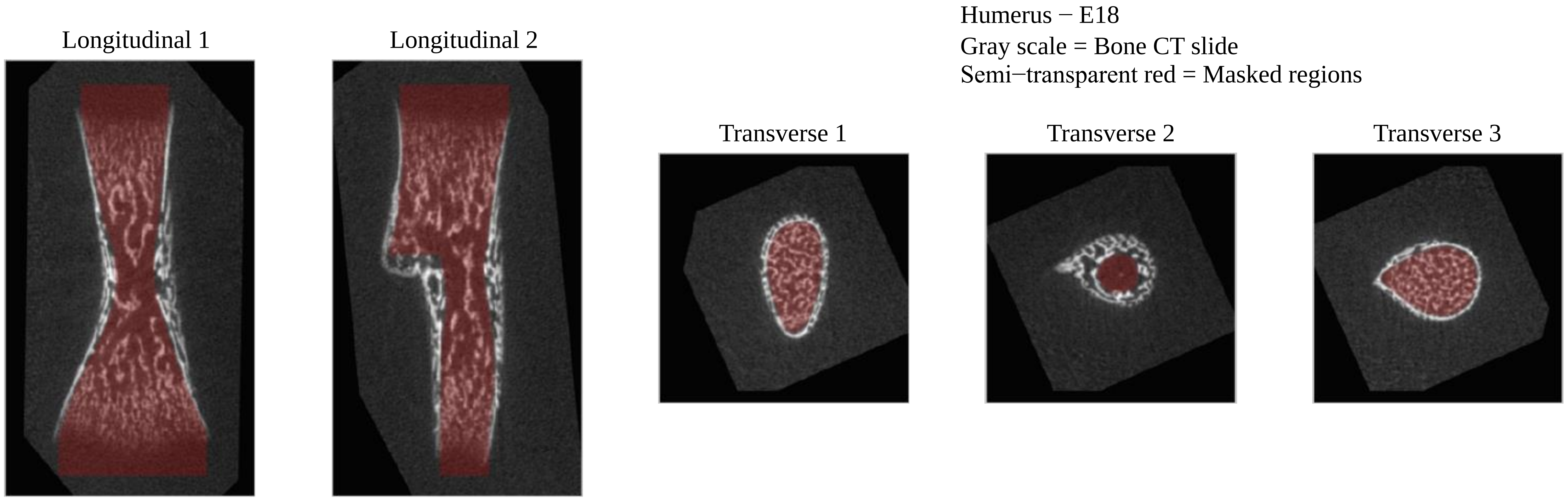
Humerus − E18
Gray scale = Bone CT slide
Semi−transparent red = Masked regions
Longitudinal 1
Longitudinal 2
Transverse 1
Transverse 2
Transverse 3

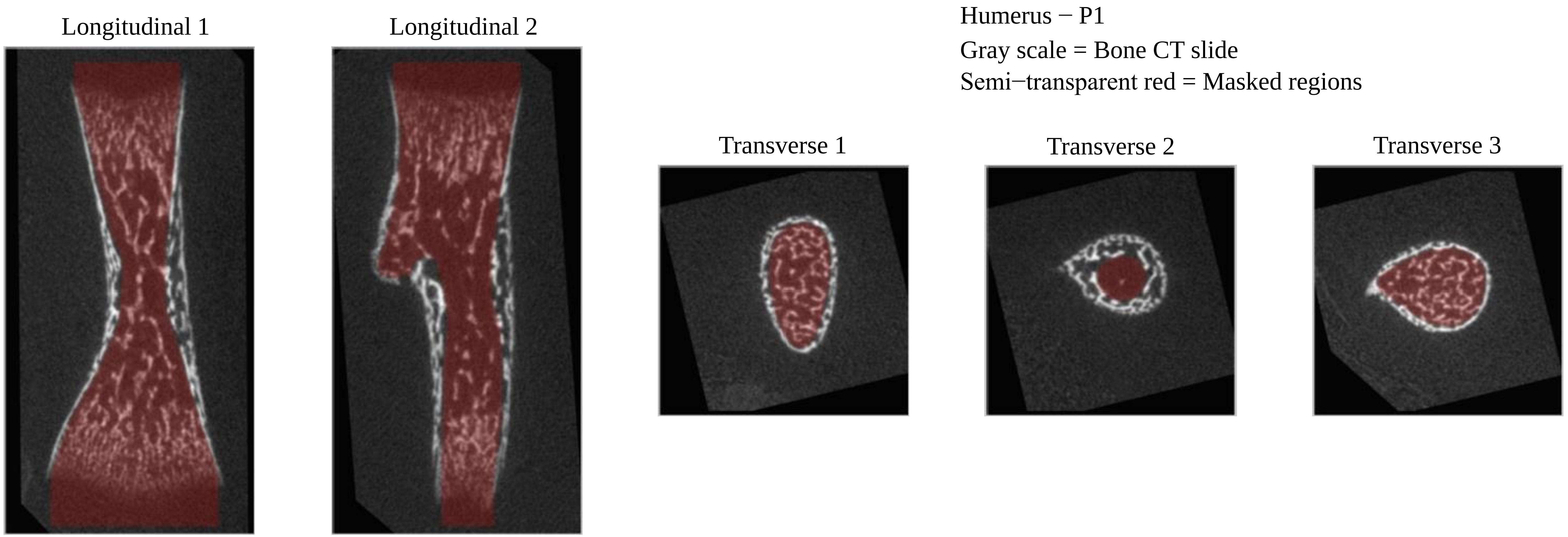

Humerus − P1
Gray scale = Bone CT slide
Semi−transparent red = Masked regions
Longitudinal 1
Longitudinal 2
Transverse 1
Transverse 2
Transverse 3

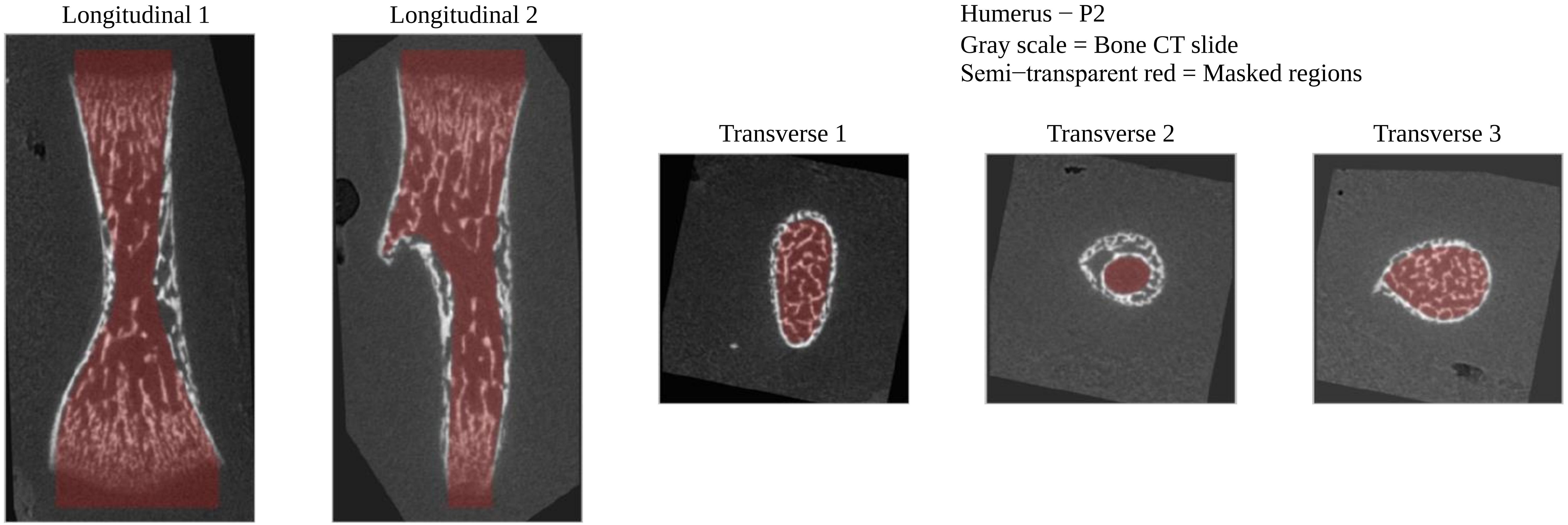
Humerus − P2
Gray scale = Bone CT slide
Semi−transparent red = Masked regions
Longitudinal 1
Longitudinal 2
Transverse 1
Transverse 2
Transverse 3

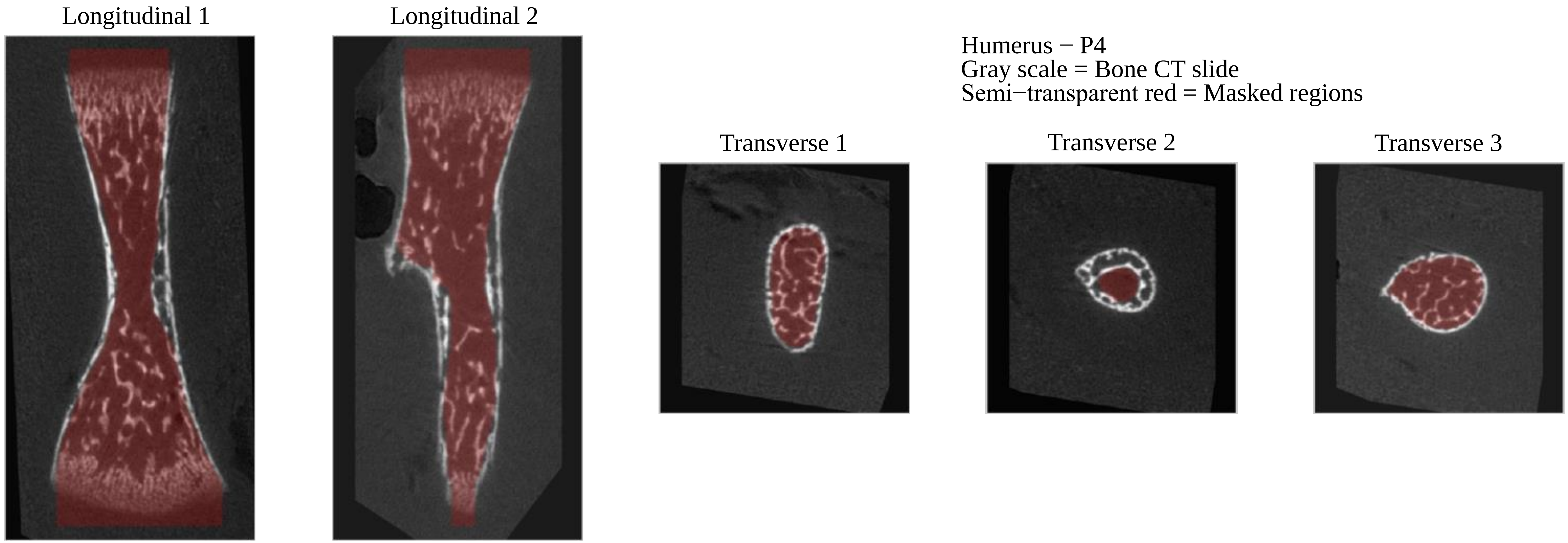
Longitudinal 1
Longitudinal 2
Humerus − P4
Gray scale = Bone CT slide
Semi−transparent red = Masked regions
Transverse 1
Transverse 2
Transverse 3

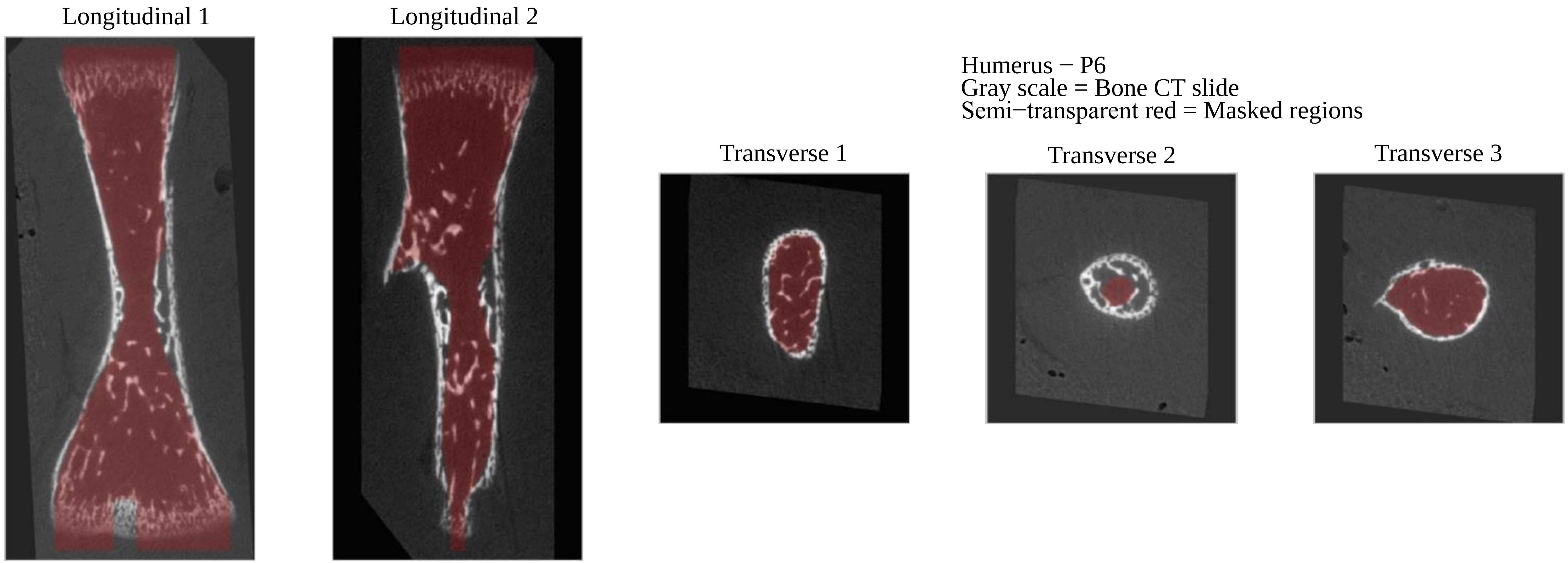
Longitudinal 1
Longitudinal 2
Humerus − P6
Gray scale = Bone CT slide
Semi−transparent red = Masked regions
Transverse 1
Transverse 2
Transverse 3

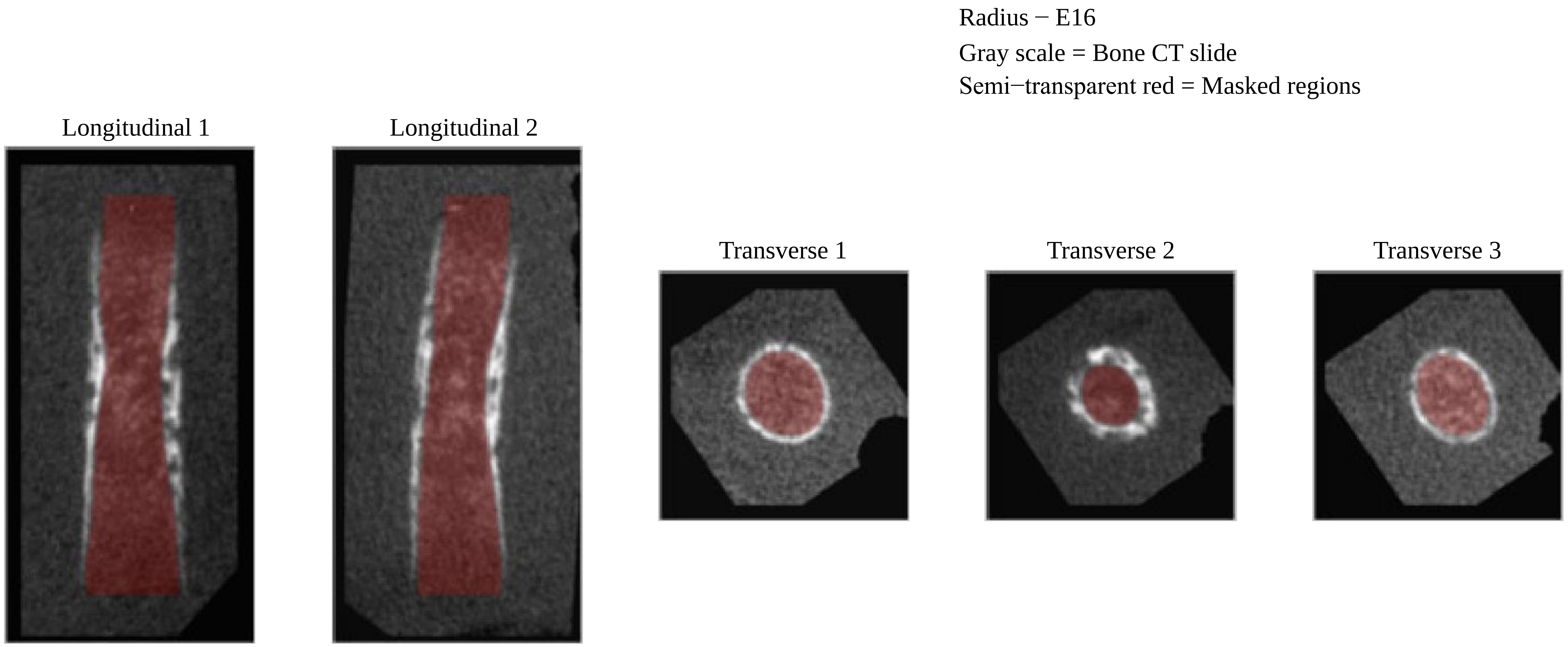
Radius − E16
Gray scale = Bone CT slide
Semi−transparent red = Masked regions
Longitudinal 1
Longitudinal 2
Transverse 1
Transverse 2
Transverse 3

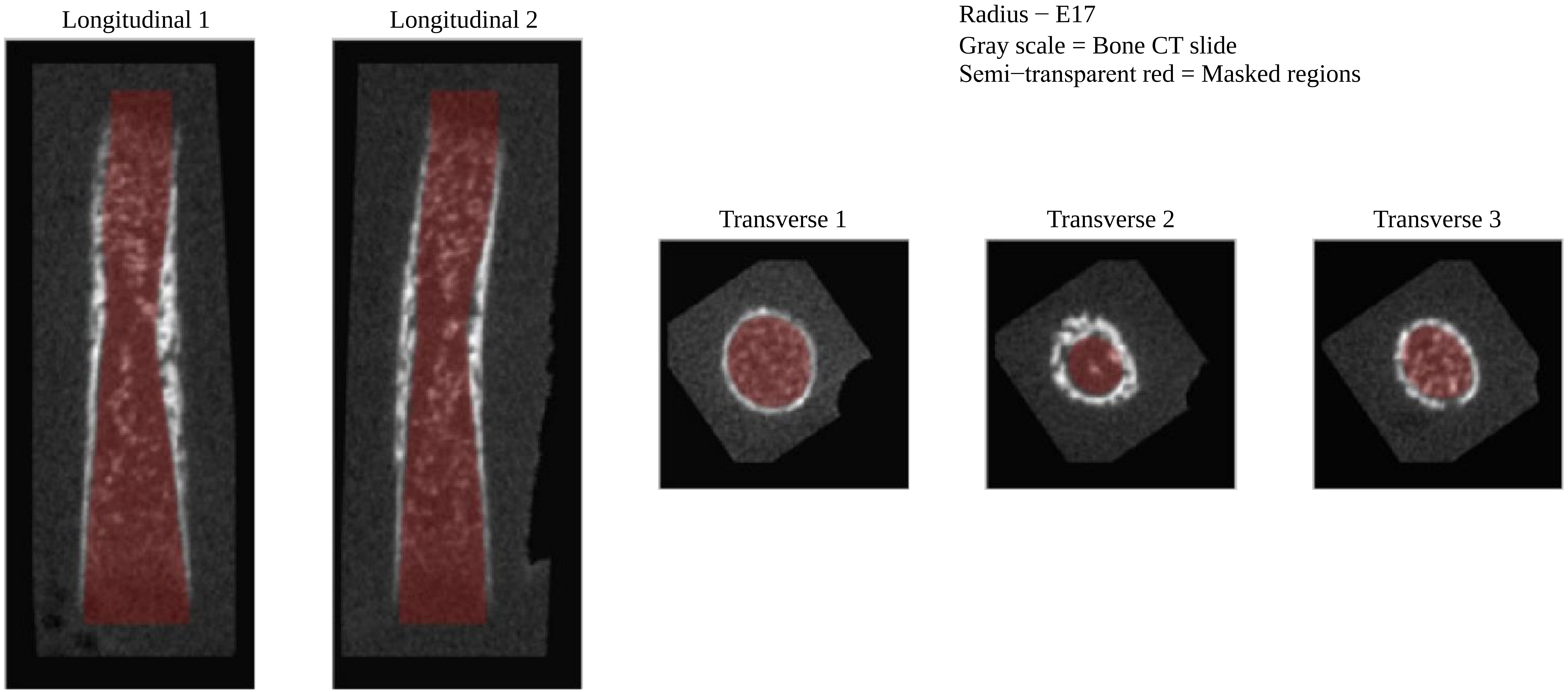
Longitudinal 1
Longitudinal 2
Radius − E17
Gray scale = Bone CT slide
Semi−transparent red = Masked regions
Transverse 1
Transverse 2
Transverse 3

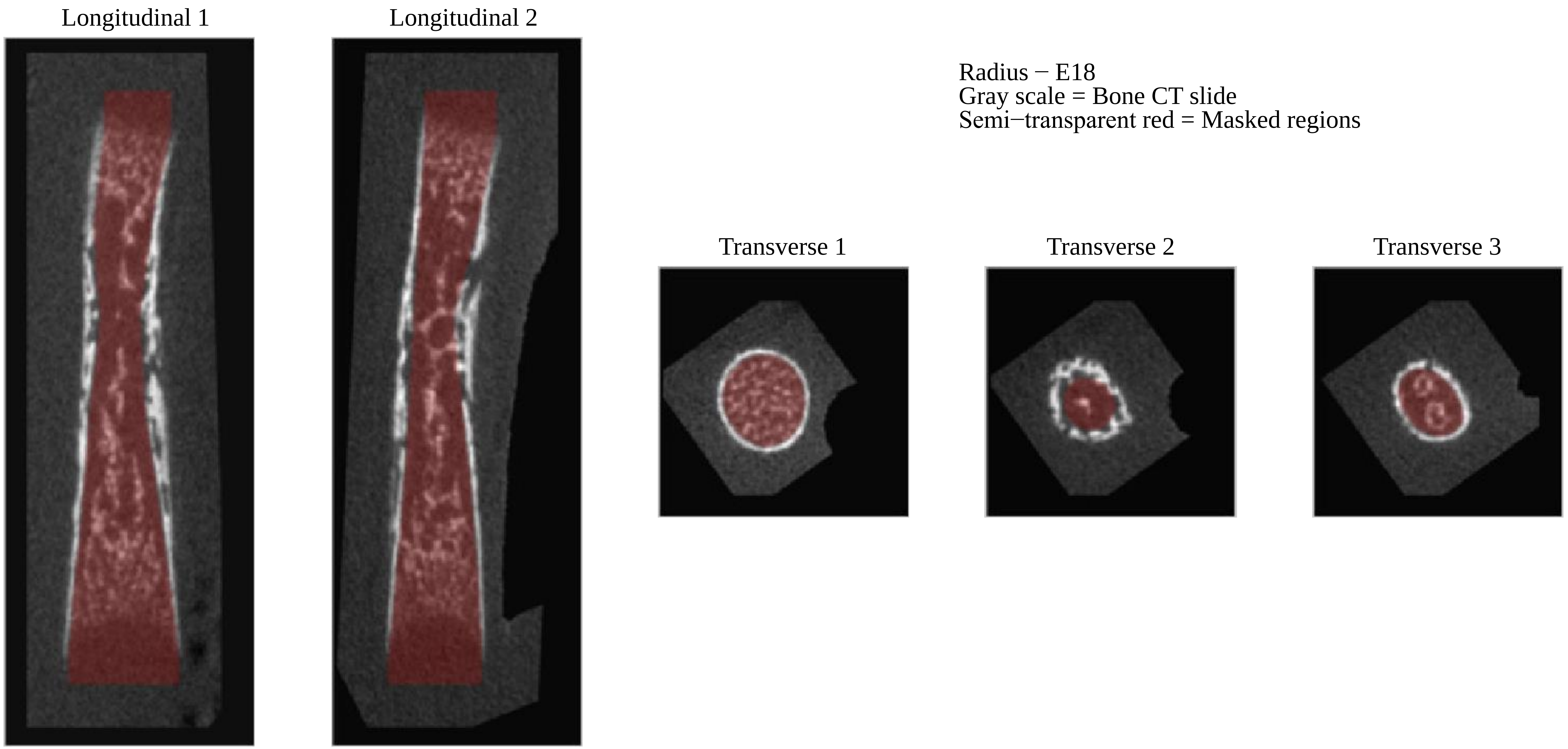
Longitudinal 1
Longitudinal 2
Radius − E18
Gray scale = Bone CT slide
Semi−transparent red = Masked regions
Transverse 1
Transverse 2
Transverse 3

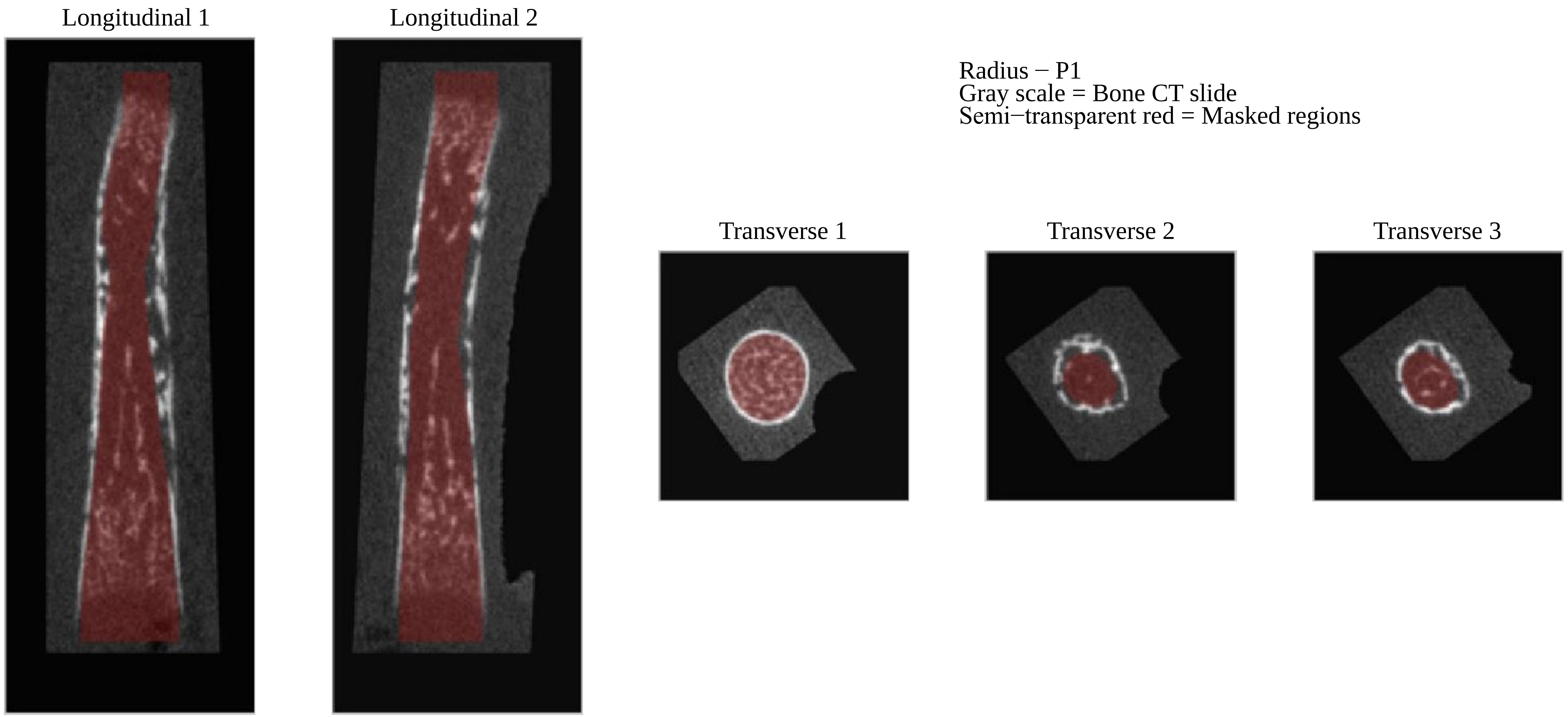
Longitudinal 1
Longitudinal 2
Radius − P1
Gray scale = Bone CT slide
Semi−transparent red = Masked regions
Transverse 1
Transverse 2
Transverse 3

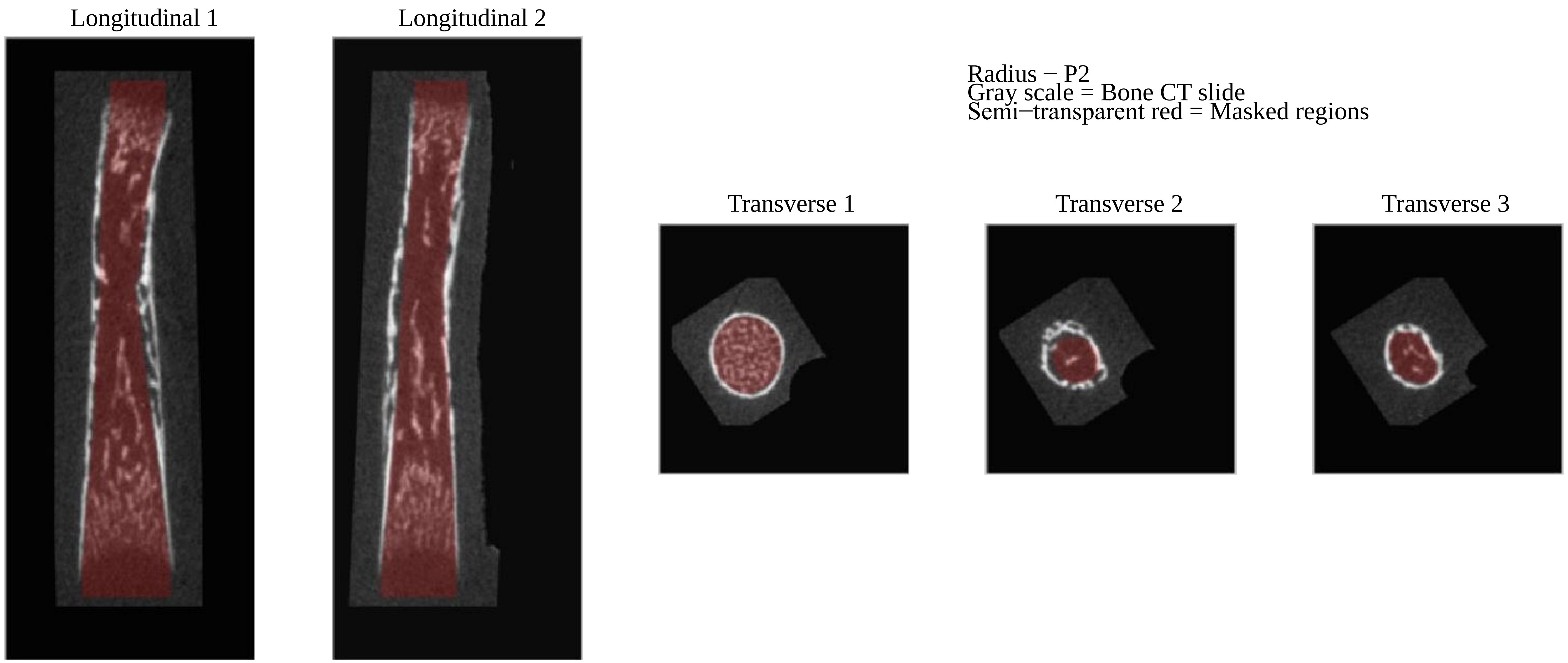
Longitudinal 1
Longitudinal 2
Radius − P2
Gray scale = Bone CT slide
Semi−transparent red = Masked regions
Transverse 1
Transverse 2
Transverse 3

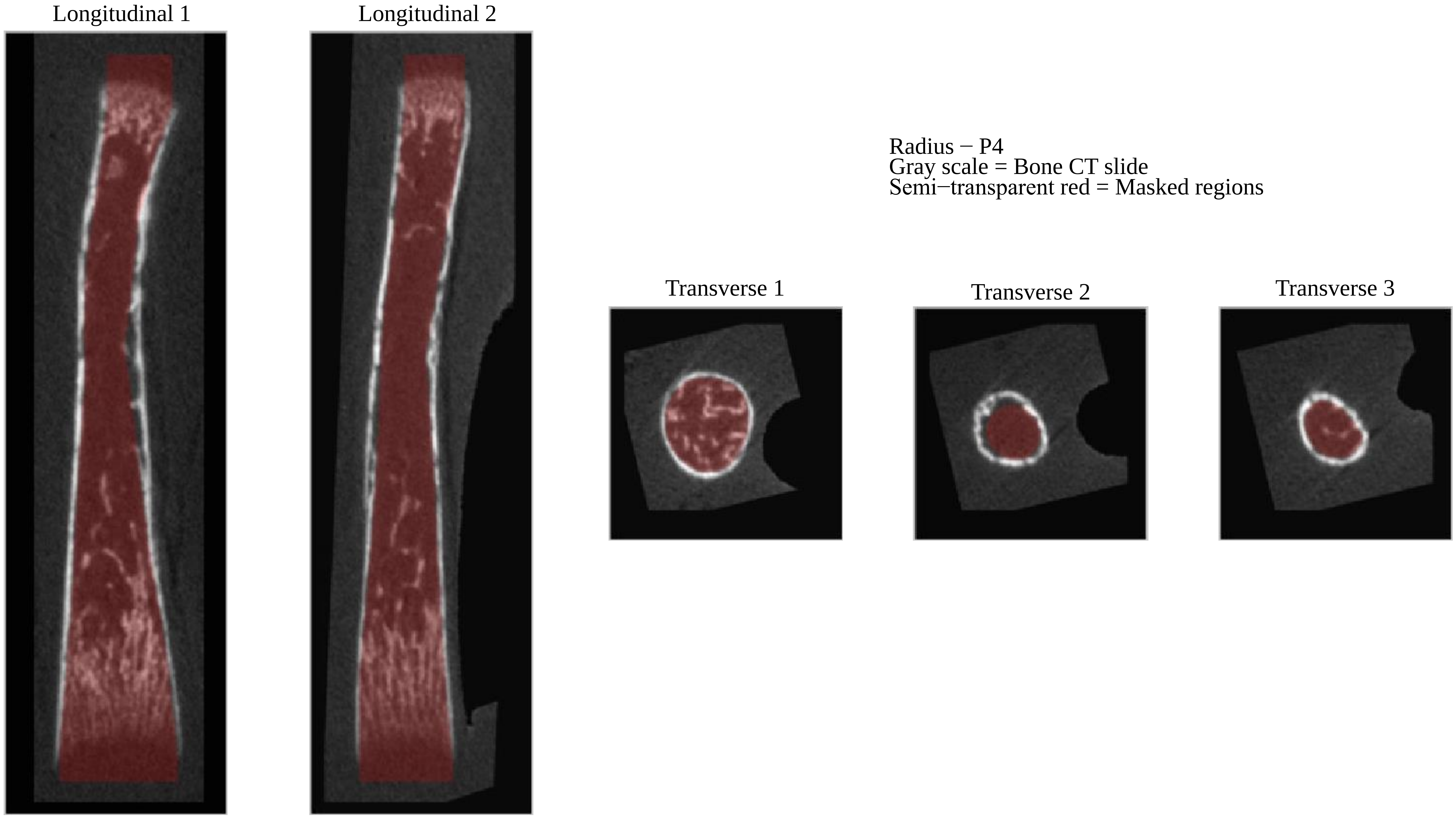
Longitudinal 1
Longitudinal 2
Radius − P4
Gray scale = Bone CT slide
Semi−transparent red = Masked regions
Transverse 1
Transverse 2
Transverse 3

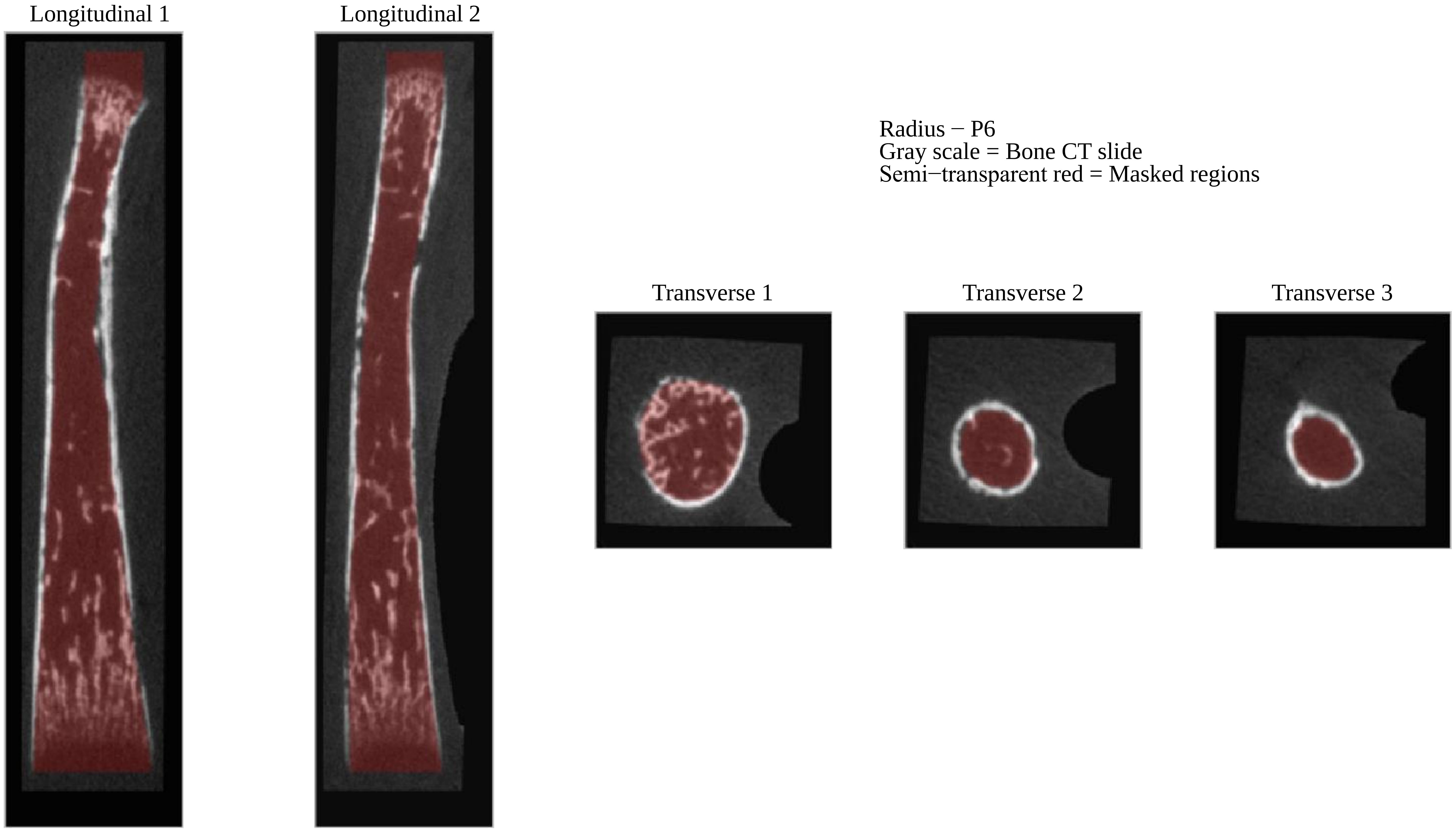
Longitudinal 1
Longitudinal 2
Radius − P6
Gray scale = Bone CT slide
Semi−transparent red = Masked regions
Transverse 1
Transverse 2
Transverse 3

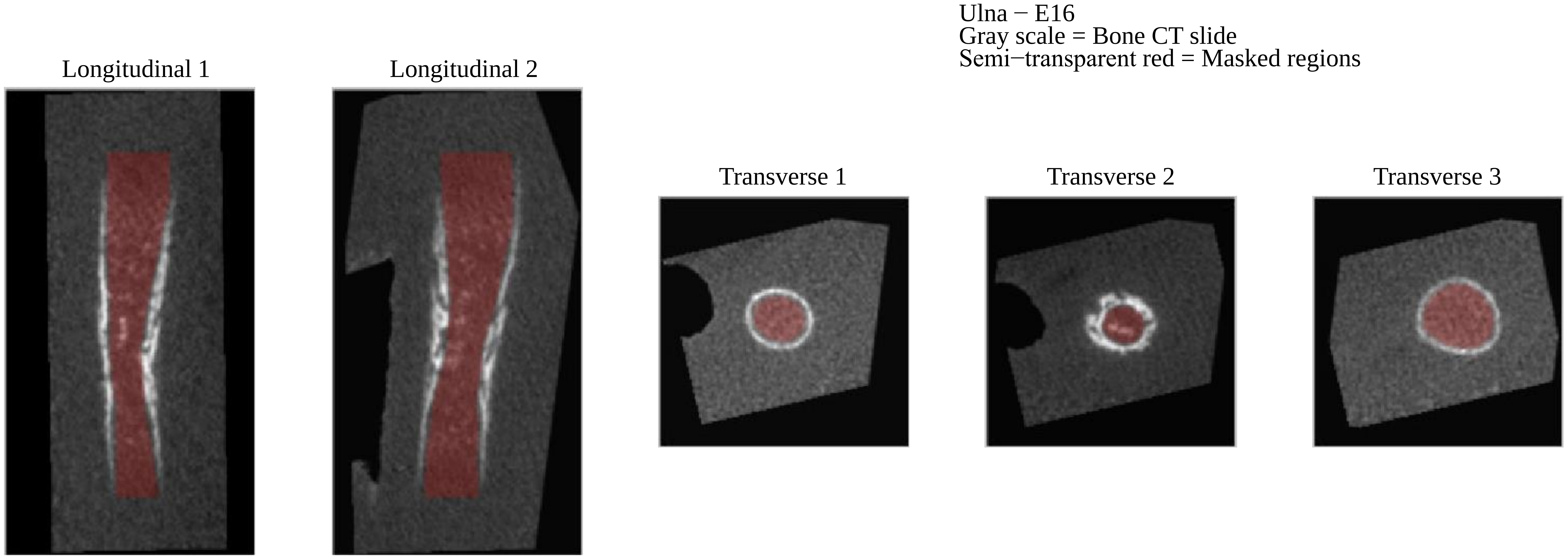
Ulna − E16
Gray scale = Bone CT slide
Semi−transparent red = Masked regions
Longitudinal 1
Longitudinal 2
Transverse 1
Transverse 2
Transverse 3

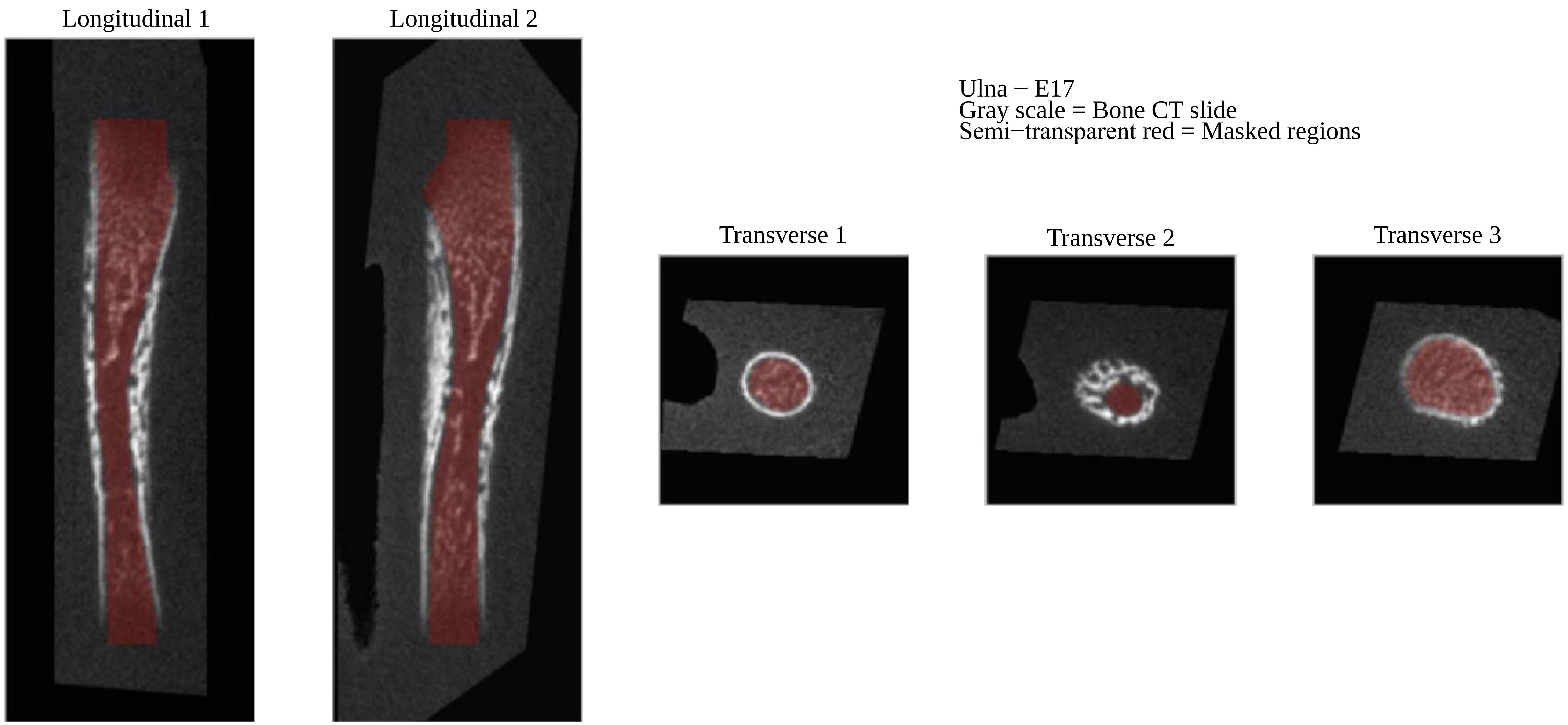
Longitudinal 1
Longitudinal 2
Ulna − E17
Gray scale = Bone CT slide
Semi−transparent red = Masked regions
Transverse 1
Transverse 2
Transverse 3

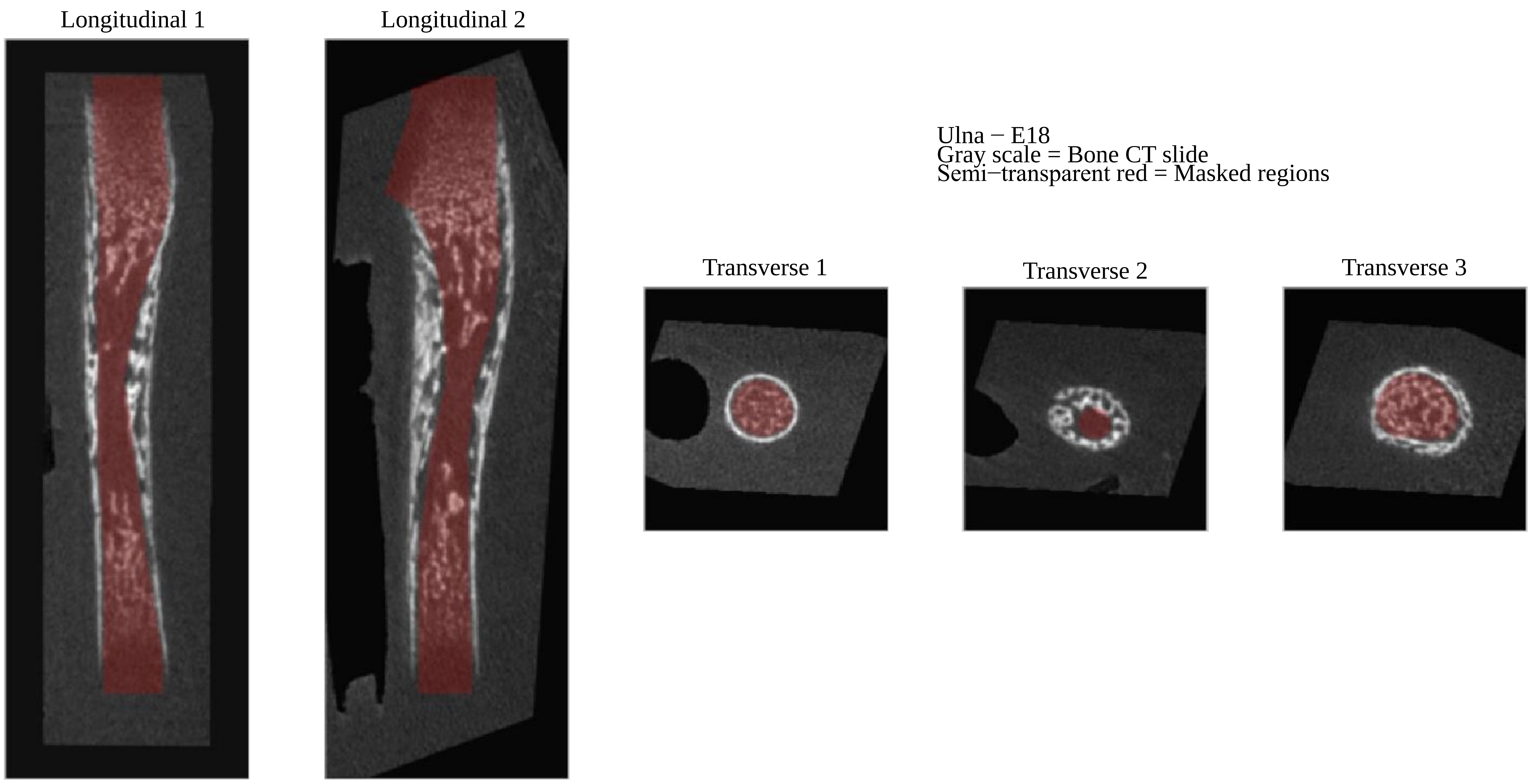
Longitudinal 1
Longitudinal 2
Ulna − E18
Gray scale = Bone CT slide
Semi−transparent red = Masked regions
Transverse 1
Transverse 2
Transverse 3

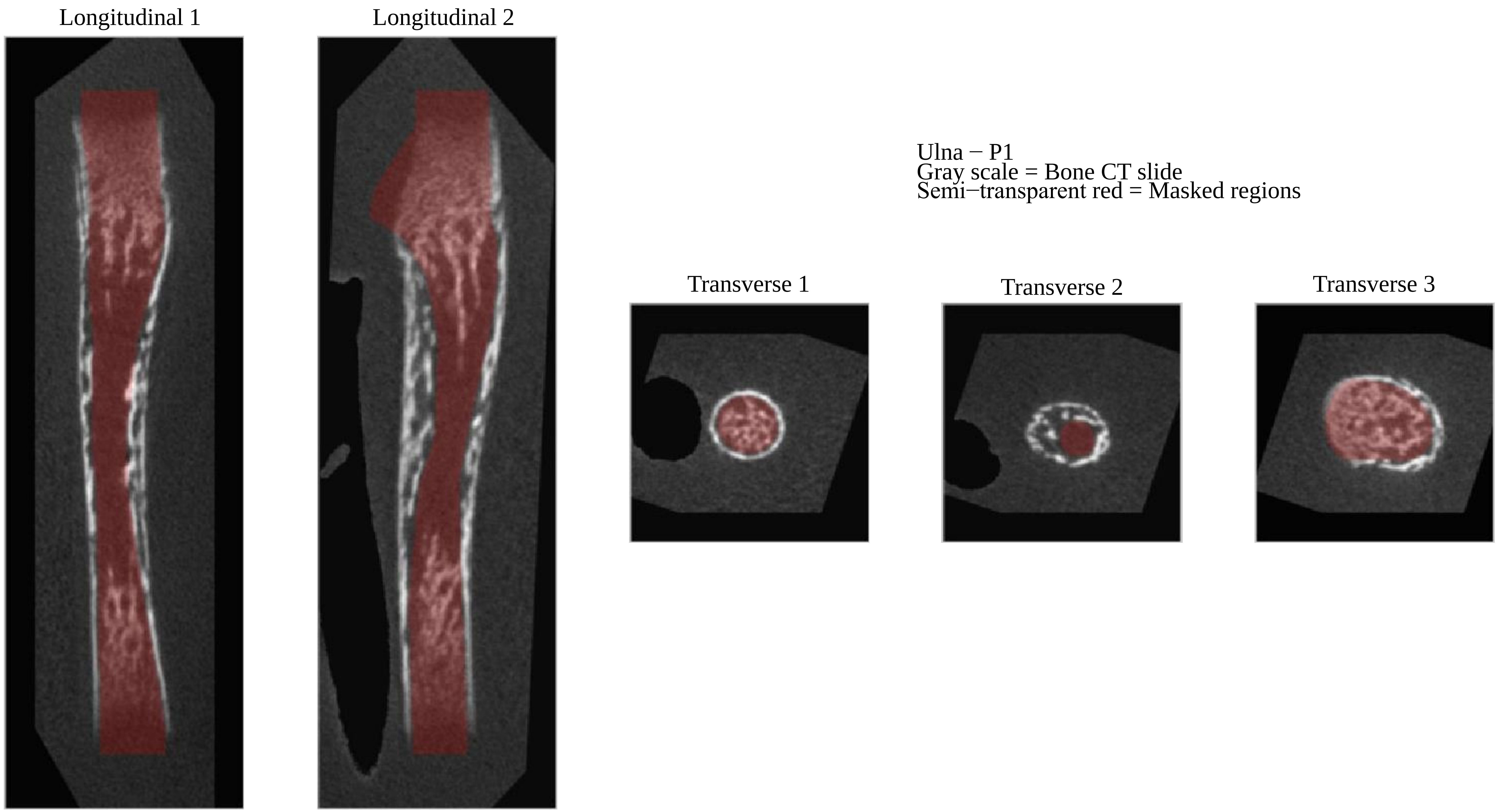
Longitudinal 1
Longitudinal 2
Ulna − P1
Gray scale = Bone CT slide
Semi−transparent red = Masked regions
Transverse 1
Transverse 2
Transverse 3

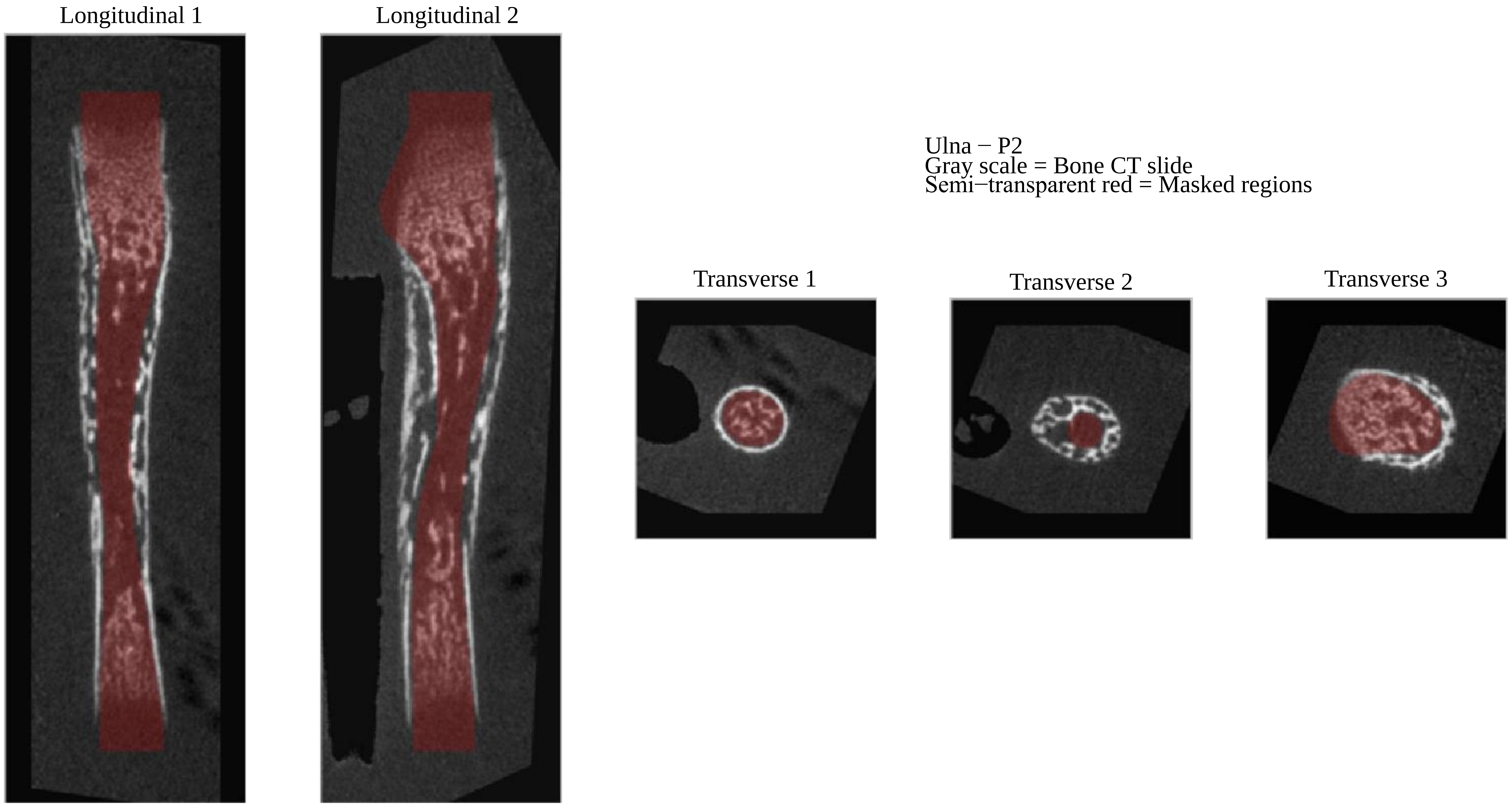
Longitudinal 1
Longitudinal 2
Ulna − P2
Gray scale = Bone CT slide
Semi−transparent red = Masked regions
Transverse 1
Transverse 2
Transverse 3

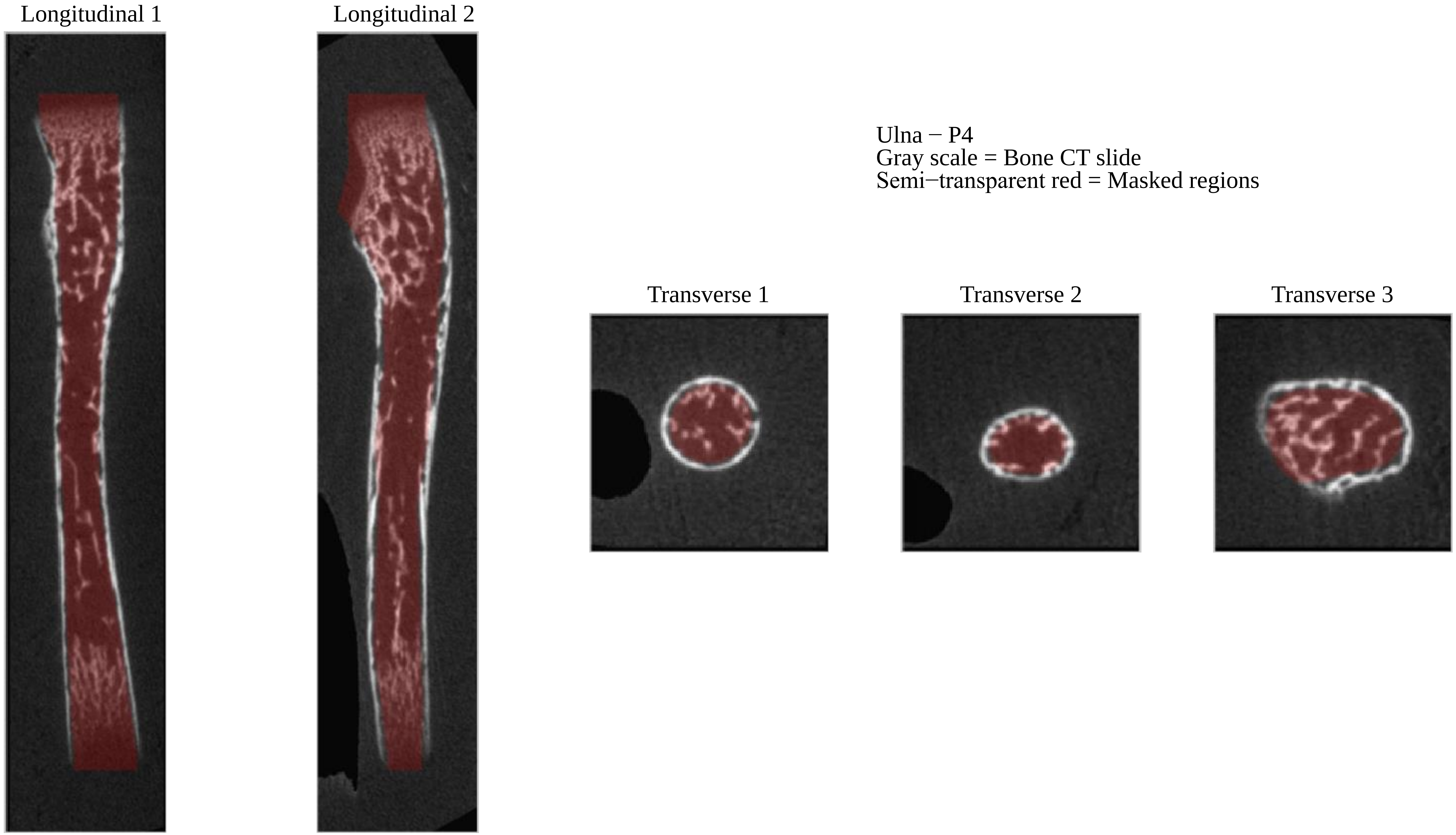
Longitudinal 1
Longitudinal 2
Ulna − P4
Gray scale = Bone CT slide
Semi−transparent red = Masked regions
Transverse 1
Transverse 2
Transverse 3

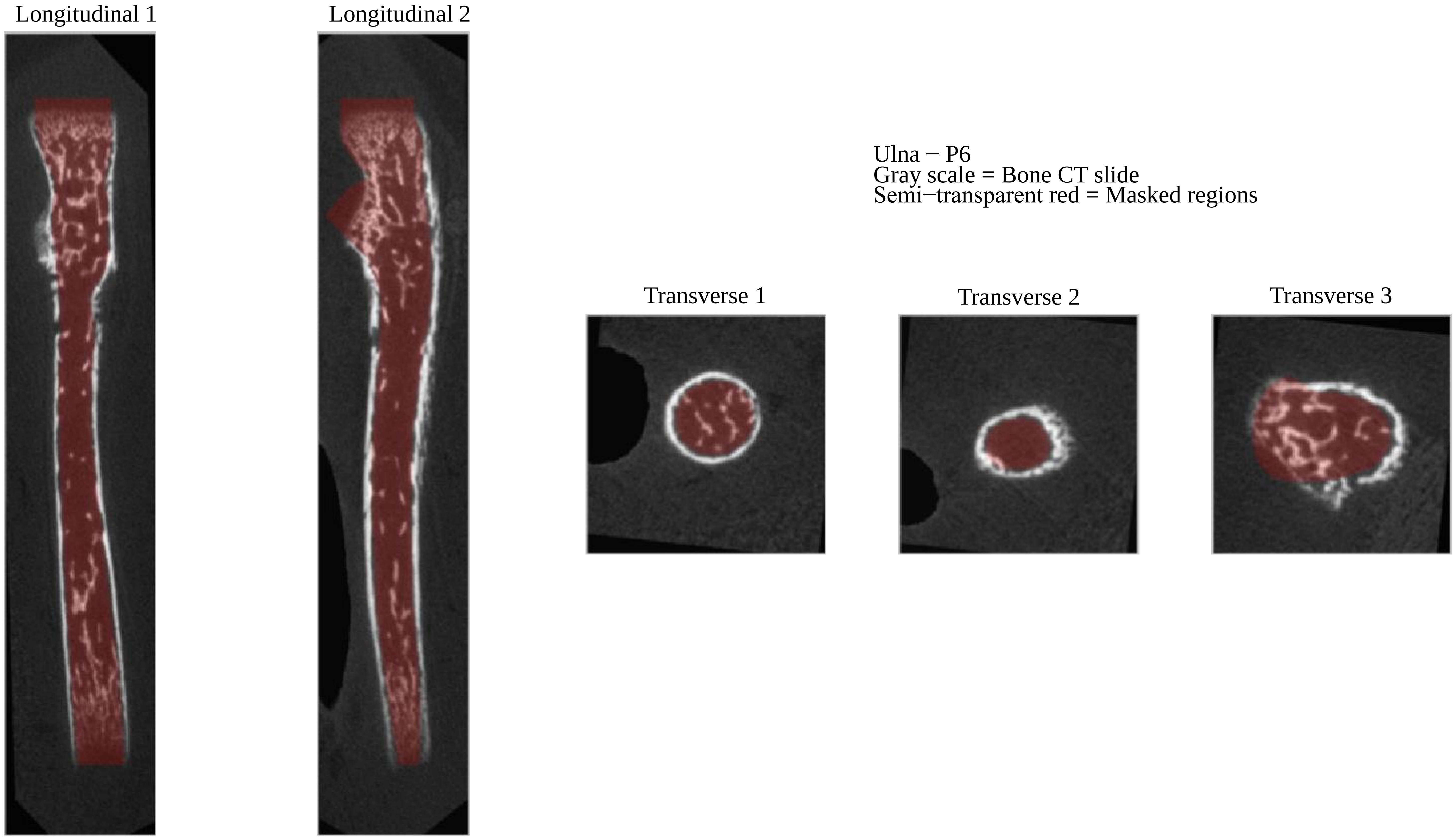
Longitudinal 1
Longitudinal 2
Ulna − P6
Gray scale = Bone CT slide
Semi−transparent red = Masked regions
Transverse 1
Transverse 2
Transverse 3

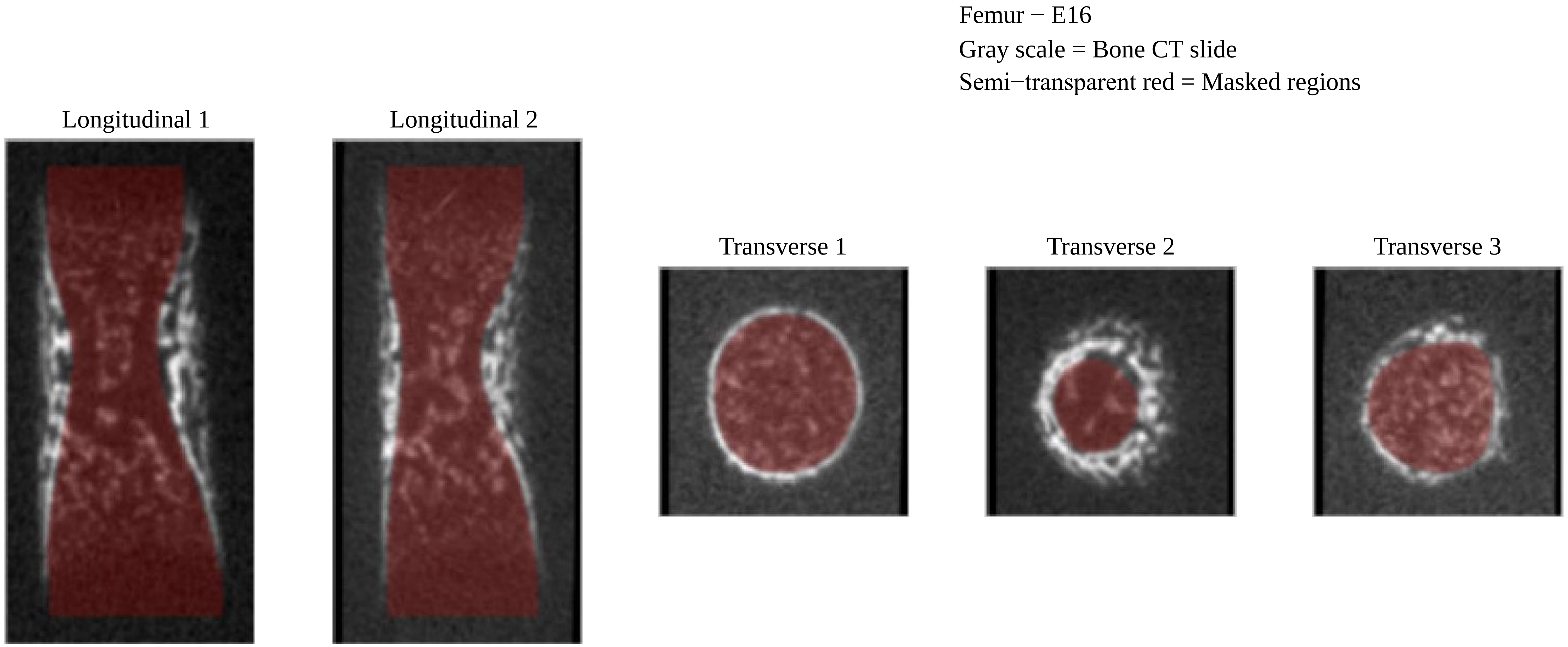

Femur − E16
Gray scale = Bone CT slide
Semi−transparent red = Masked regions
Longitudinal 1
Longitudinal 2
Transverse 1
Transverse 2
Transverse 3

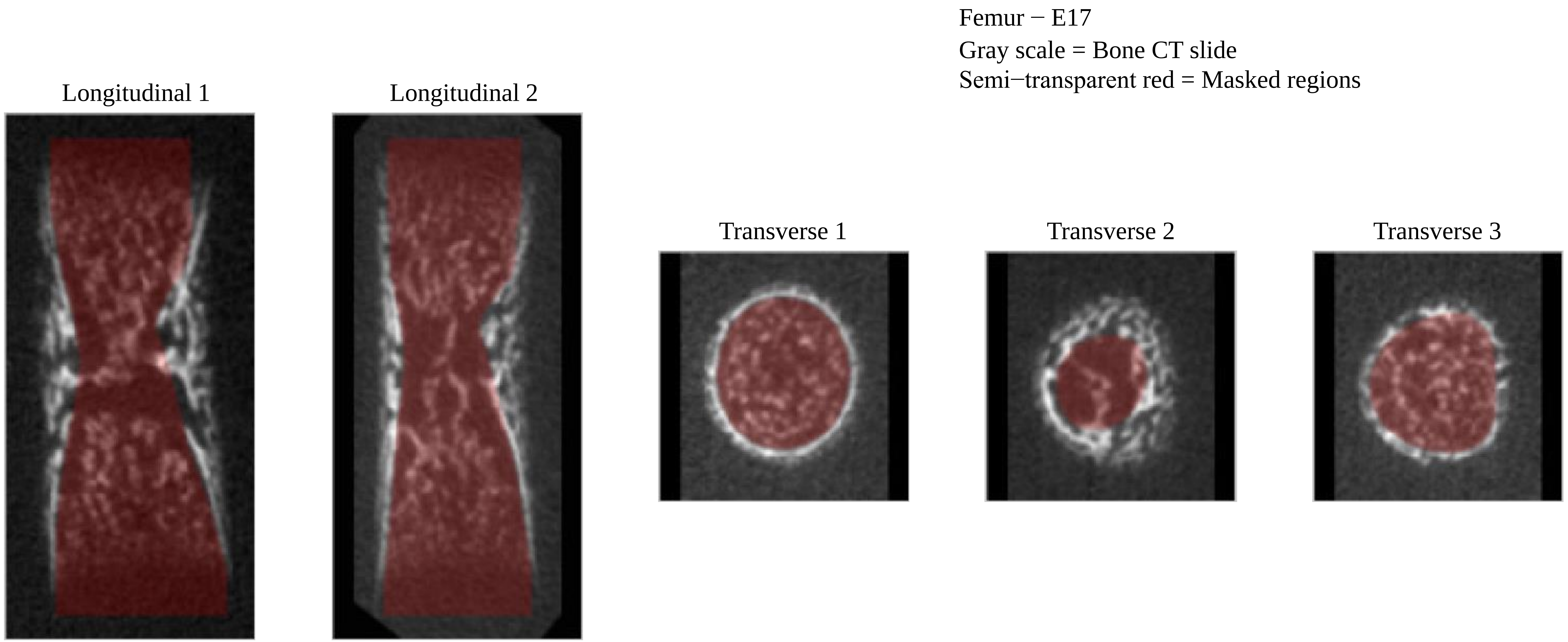
Femur − E17
Gray scale = Bone CT slide
Semi−transparent red = Masked regions
Longitudinal 1
Longitudinal 2
Transverse 1
Transverse 2
Transverse 3

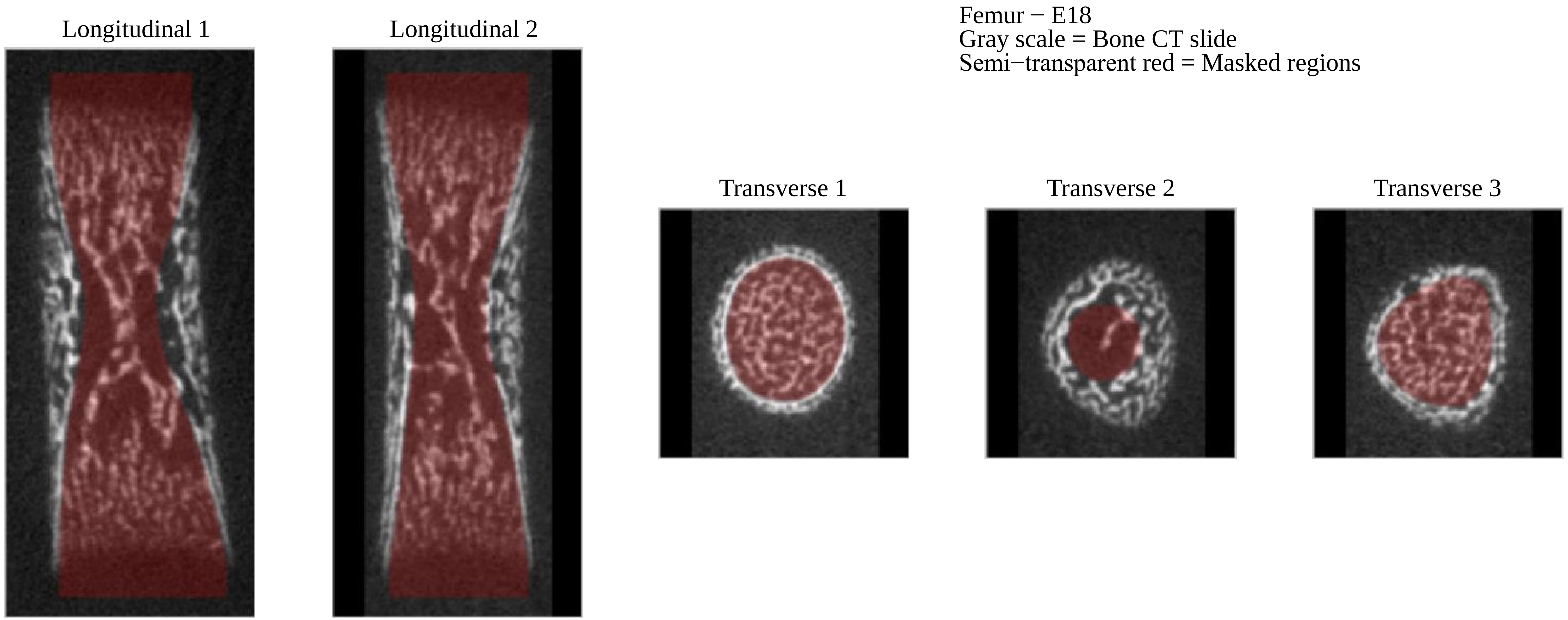
Femur − E18
Gray scale = Bone CT slide
Semi−transparent red = Masked regions
Longitudinal 1
Longitudinal 2
Transverse 1
Transverse 2
Transverse 3

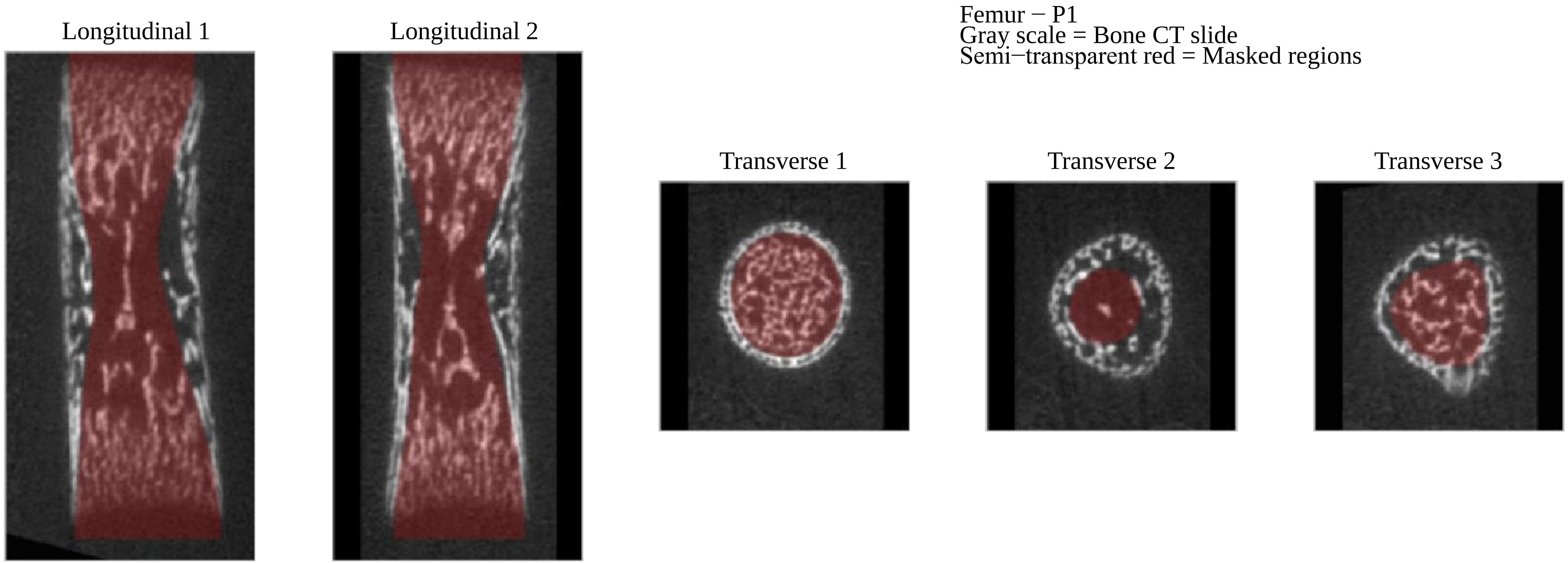
Femur − P1
Gray scale = Bone CT slide
Semi−transparent red = Masked regions
Longitudinal 1
Longitudinal 2
Transverse 1
Transverse 2
Transverse 3

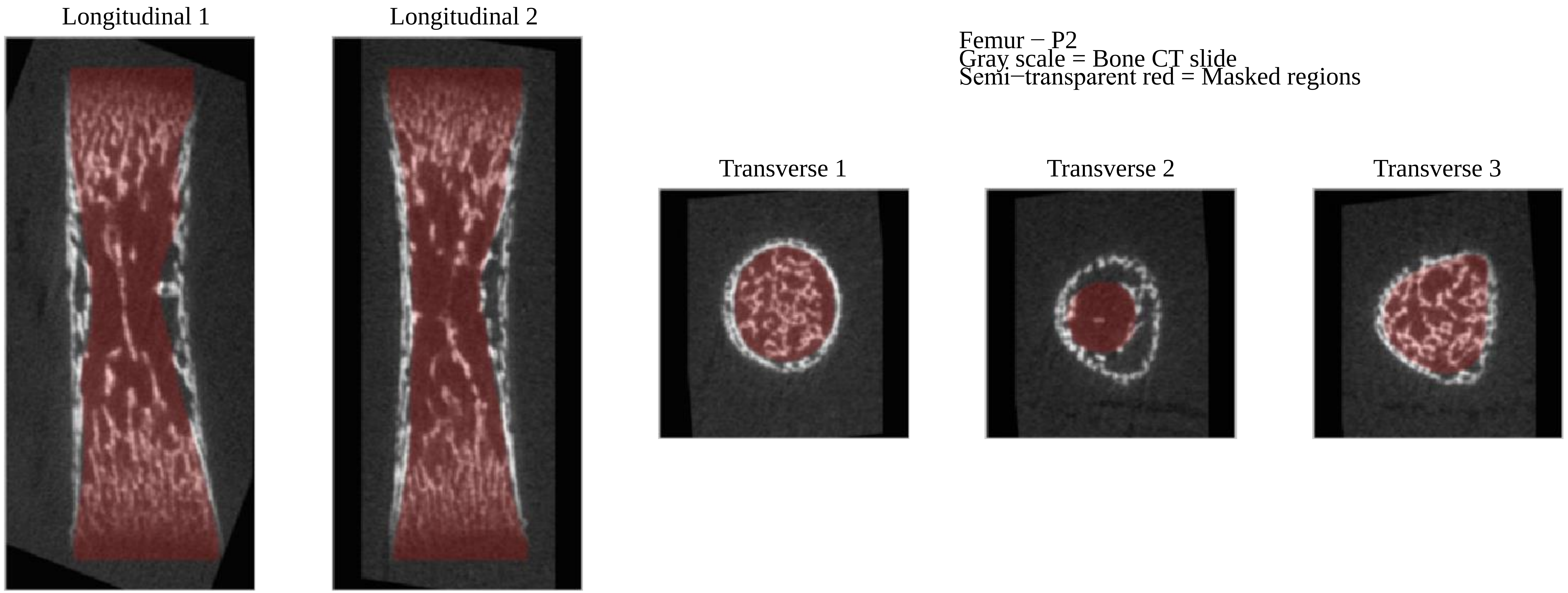
Longitudinal 1
Longitudinal 2
Femur − P2
Gray scale = Bone CT slide
Semi−transparent red = Masked regions
Transverse 1
Transverse 2
Transverse 3

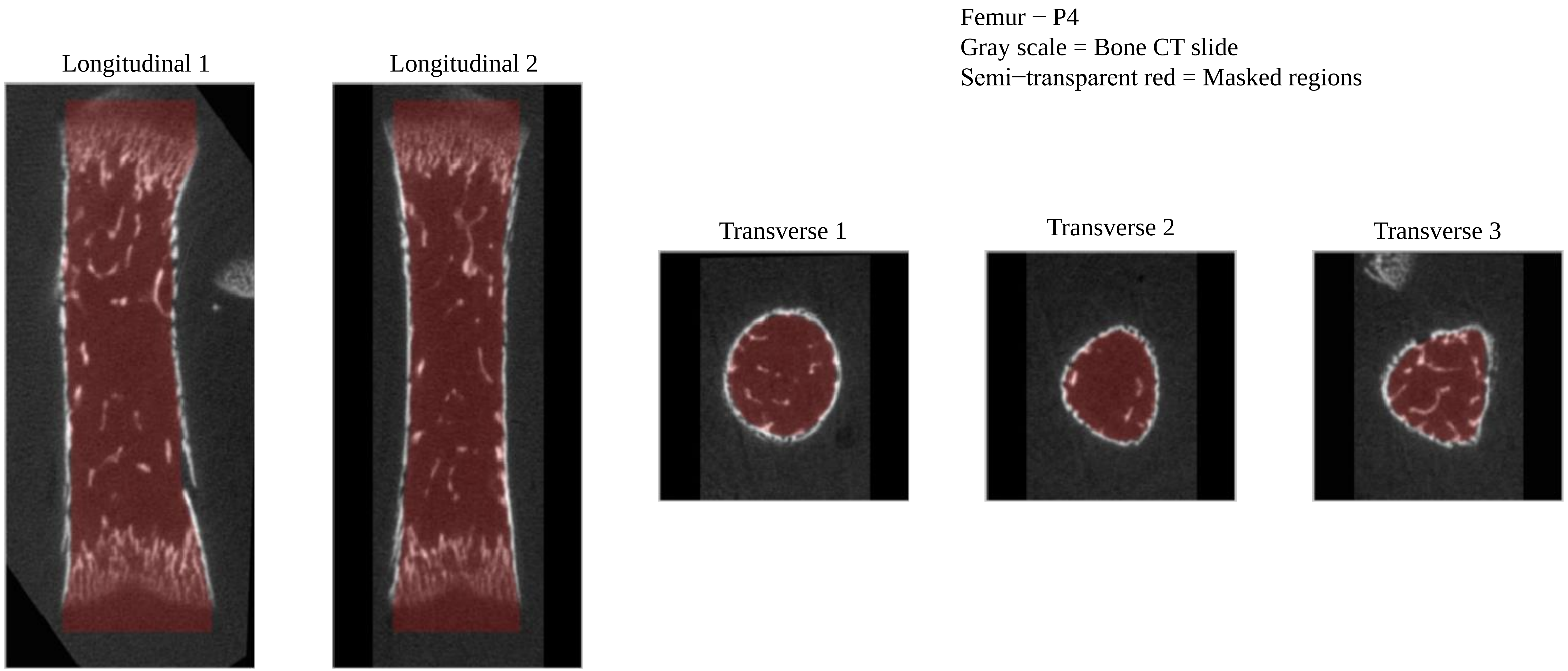

Femur − P4
Gray scale = Bone CT slide
Semi−transparent red = Masked regions
Longitudinal 1
Longitudinal 2
Transverse 1
Transverse 2
Transverse 3

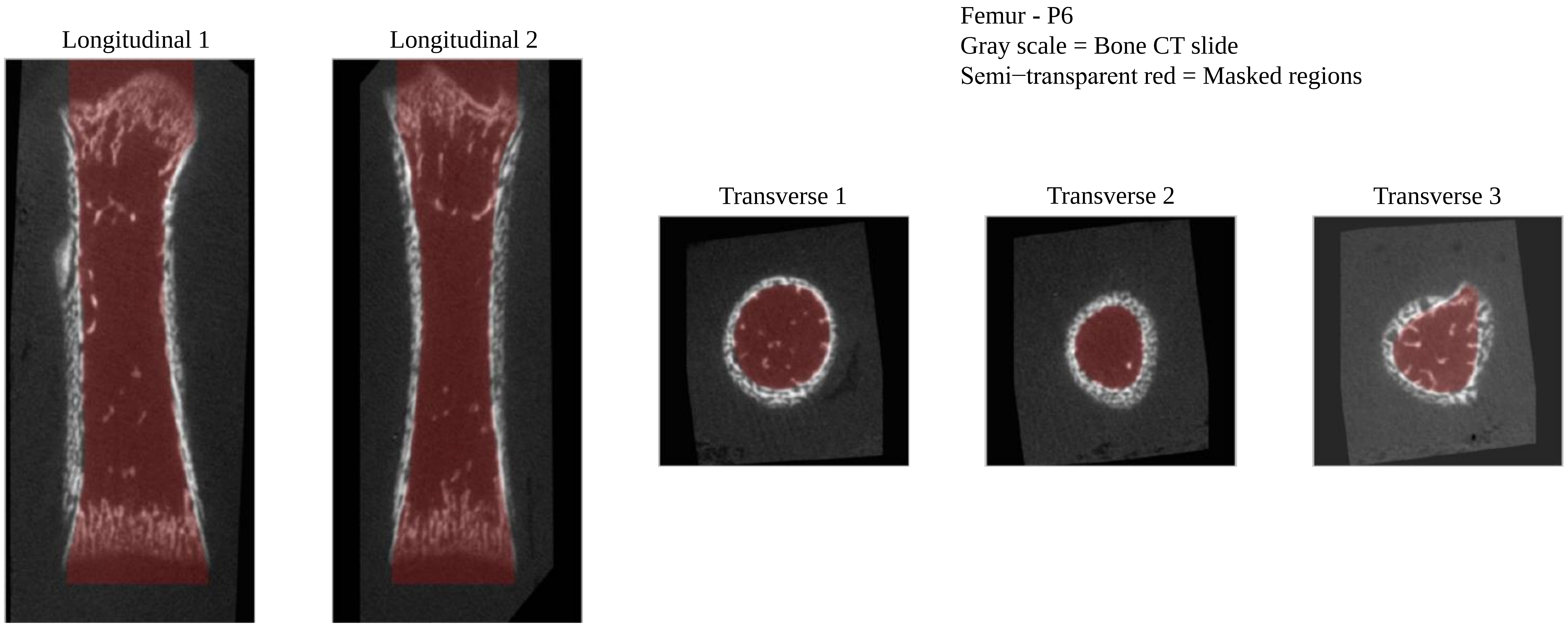
Femur - P6
Gray scale = Bone CT slide
Semi−transparent red = Masked regions
Longitudinal 1
Longitudinal 2
Transverse 1
Transverse 2
Transverse 3
